# Short-Term and Late-Term Effects of Psilocybin on Symptoms in Major Depression: A Randomized Clinical Trial

**DOI:** 10.1001/jamanetworkopen.2026.12589

**Published:** 2026-05-15

**Authors:** Hampus Yngwe, Pontus Plavén-Sigray, Carl Johan Ekman, Eva Henje, Anders Berglund, Mikael Tiger, Maria Beckman, Johan Lundberg

**Affiliations:** 1 Centre for Psychiatry Research, Department of Clinical Neuroscience, Karolinska Institutet, and Stockholm Health Care Services, Region Stockholm, Stockholm, Sweden; 2 Neurobiology Research Unit, Copenhagen University Hospital, Rigshospitalet, Copenhagen, Denmark; 3 Department of Clinical Sciences, Umeå University, Umeå, Sweden; 4 Epistat AB, Uppsala, Sweden

## Abstract

**Importance:**

Psilocybin has been proposed as a rapid-acting antidepressant (onset <2 weeks) with sustained effects (>6 weeks), but evidence from randomized clinical trials remains limited, particularly in the broader major depressive disorder (MDD) population.

**Objective:**

To assess short-term and long-term antidepressant effects of psilocybin therapy in patients with MDD.

**Design, Setting, and Participants:**

This double-blind, placebo-controlled randomized clinical trial of participants diagnosed with moderate to severe recurrent MDD was conducted at the Northern Stockholm Psychiatric Clinic between January 26, 2021, and February 19, 2024. Statistical analysis was performed from February 20, 2024, to June 20, 2025.

**Interventions:**

Participants received a single dose of psilocybin (25 mg) or active placebo (niacin, 100 mg) and 5 psychotherapeutic support sessions during 17 days.

**Main Outcomes and Measures:**

The primary end point was between-group difference in change in Montgomery-Åsberg Depression Rating Scale (MADRS) score from baseline to day 8. Secondary end points included MADRS scores on days 15, 42, and 365, as well as monthly self-reports (MADRS-S) of depressive symptoms, disability, quality of life, and anxiety throughout the 365-day follow-up.

**Results:**

The study included 35 participants (21 [60%] female; mean [SD] age, 41.0 [10.1] years) diagnosed with moderate to severe recurrent MDD, with 17 randomized to the psilocybin group and 18 to the niacin group. The study met its primary end point with a significant mean between-group difference (model estimated) in change in MADRS score on day 8 (−7.27; 95% CI, −12.89 to −1.65; *P* = .01) in favor of psilocybin. The between-group difference was significant also on days 15 (mean difference, −11.03; 95% CI, −16.65 to −5.42; *P* < .001) and 42 (mean difference, −8.33; −13.94 to −2.71; *P* = .004) but no longer on day 365 (mean difference, −3.68; −9.30 to 1.94; *P* = .20). For MADRS-S, the psilocybin group had a significantly greater reduction beginning at day 2 (mean difference, −9.58; 95% CI, −16.05 to −3.11; *P* = .004), with group differences persisting through day 102 (mean difference, −6.60; 95% CI, −13.01 to −0.19; *P* = .04) and then isolated effects at days 283 and 343. Most reported treatment-emergent adverse events were transient and of mild to moderate severity. No drug-related serious adverse events were reported. Two participants in the psilocybin group reported persistent, severe anxiety that required medical attention.

**Conclusions and Relevance:**

In this randomized clinical trial of MDD, a single dose of psilocybin was associated with rapid antidepressant effects, observed by day 2 and persisting for more than 3 months on secondary outcomes; psilocybin was generally well tolerated, but some individuals required additional support after dosing due to anxiety. These results suggest that psilocybin may provide a rapid and relatively long-lasting antidepressant effect on major depressive disorder, warranting further investigation into repeated dosing or adjunctive treatment strategies.

**Trial Registration:**

ClinicalTrials.gov Identifier: NCT04630964

## Key Points

**Question:**

Does a single dose of psilocybin have short- and long-term antidepressant effects on patients with recurrent major depressive disorder?

**Findings:**

In this randomized clinical trial of 35 participants, a single 25-mg dose of psilocybin produced a significantly greater reduction in depressive symptoms compared with 100 mg of niacin according to the Montgomery-Åsberg Depression Rating Scale score on day 8. The duration of effect was more than 3 months.

**Meaning:**

This study’s findings suggest that psilocybin may provide a rapid and relatively long-lasting antidepressant effect on major depressive disorder, warranting further investigation into repeated dosing or adjunctive treatment strategies.

## Teaser

This randomized clinical trial assesses rapid, short-term, and long-term antidepressant effects of psilocybin therapy in patients with major depressive disorder.

## Introduction

Major depressive disorder (MDD) is a debilitating condition and an increasing health issue worldwide.^1^ Two-thirds of the patients do not remit after first-line treatment with serotonin reuptake inhibitors (SSRIs), and a substantial fraction do not remit after multiple treatment attempts.^2^ Response time is typically 2 to 6 weeks, and daily administration is often continued for years to reduce risk of relapse.^3^ Because antidepressants commonly have adverse effects, adherence and quality of life are often impaired.^4,5^ Major objectives in MDD research have thus been increasing remission rates, shortening response time, providing better relapse protection, and increasing tolerability.

Rapid-acting agents, such as classical psychedelics (eg, psilocybin) and ketamine, show promise in this regard.^6,7,8^ Ketamine’s effect is short-lived,^8^ but emerging data indicate that a single dose of psilocybin has antidepressant effects extending to 6 weeks and beyond.^9^ Although many psilocybin trials targeted subgroups with treatment resistance and cancer-related depression, the overall MDD population (up to 90% of all cases)^10^ may equally benefit from the rapid effects. Should a single dose prevent relapse in the long term also in this population, the adverse effects associated with daily medication could be avoided.

Primary efficacy outcomes in psilocybin trials have been measured at 2 to 6 weeks,^11^ but secondary outcomes indicate a more rapid response,^12^ which is yet to be demonstrated for the primary end point. Another advantage commonly attributed to psilocybin is its potential for long-term efficacy. The supporting evidence, however, remains limited because prior randomized clinical trials^13,14,15,16^ (RCTs) often used crossover designs or experienced selection bias. Although follow-up studies report within-group effects for 6 to 12 months, without a control group and antidepressant monitoring, the natural course of the disorder and treatment confounds cannot be controlled for.^17,18,19^

Lastly, the subjective experiences of psychedelics raise concerns about functional unblinding and expectancy bias.^20,21,22^ Double-blind RCTs are typically considered to have high internal validity for estimating treatment effects^23,24^; however, the strength of the evidence depends on successful blinding. Active placebos, such as niacin or low-dose comparators, may introduce uncertainty about allocation, but the blinding integrity has rarely been reported in clinical trials of psychedelics and never in studies of psilocybin for MDD.^12,25,26^ Many previous trials also included past psychedelic users, with an inherent increased risk of functional unblinding.^11^

Although previous findings are promising, evidence of rapid effects remain inconclusive, and long-term, placebo-controlled follow-up data are lacking. The blinding issue also casts some doubt on the reliability of the findings because reported effect sizes may be inflated by placebo effects. This study aims to address these issues. The primary objective was assessing the short-term antidepressant effects of a single, oral, 25-mg dose of psilocybin in MDD already on day 8. Secondary objectives included a unique 12-month placebo-controlled follow-up of efficacy and tolerability. We also monitored antidepressant prescription in the follow-up period and tested blinding integrity.

## Methods

### Study Design Overview and Oversight

This phase 2 randomized clinical trial was designed to evaluate the efficacy and tolerability of psilocybin in the treatment of MDD. Participants were randomized 1:1 to a single oral dose of psilocybin, 25 mg, or to the active placebo niacin, 100 mg. Both groups received 1 preparatory, 1 dosing, and 3 integration psychotherapeutic support sessions during 17 days. The trial was conducted at the Northern Stockholm Psychiatric Clinic in Sweden between January 26, 2021, and February 19, 2024. The complete protocol also included magnetic resonance imaging, positron emission tomography, and sampling of cerebrospinal fluid and blood, to be reported later. The protocol (Supplement 1) was approved by the Swedish Ethical Review Authority and the Swedish Medical Products Agency, adhering to the Declaration of Helsinki^27^ and standards of Good Clinical Practice. All participants provided written informed consent. The study followed the Consolidated Standards of Reporting Trials (CONSORT) reporting guideline.

### Participants

**Figure 1.**
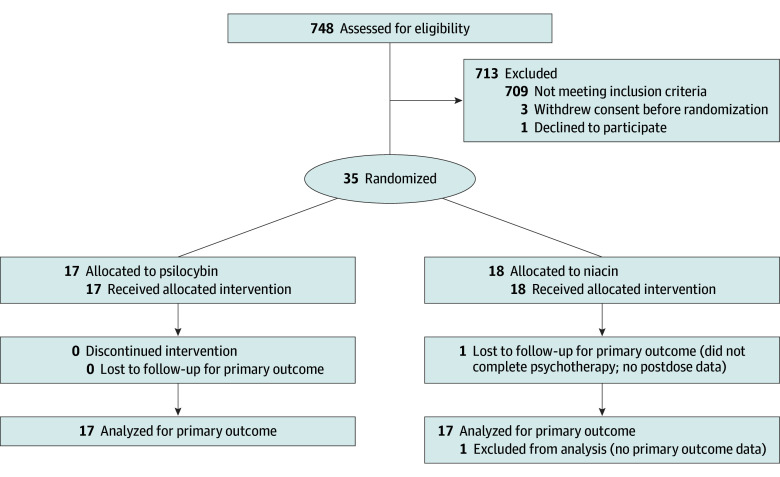
CONSORT Flow Diagram Sixty-three patients were screened.

A total of 748 Swedish-speaking candidates were recruited via social media and Google Ads and completed an initial digital and telephone prescreening, of whom 63 proceeded to in-person screening. No study-related procedures took place before informed consent had been obtained. Four participants withdrew before randomization. Seven participants needed to discontinue their antidepressants before inclusion in consultation with the attending physician. Thirty-five participants were randomized, 17 to psilocybin and 18 to niacin (Figure 1).

The primary inclusion criterion was meeting the *International Statistical Classification of Diseases and Related Health Problems, Tenth Revision (ICD-10)*^28^ criteria of an ongoing episode of recurrent MDD with a duration of 30 days or longer but less than 5 years that scored 22 points or higher on the Montgomery-Åsberg Depression Rating Scale (MADRS).^29^ Main exclusion criteria were previous psychedelics use, various medical conditions, psychotic or bipolar disorder or first-degree relatives with psychotic disorder, current substance use disorder, ongoing antidepressant therapy or psychotherapy, pregnancy, and suicidality (for complete inclusion and exclusion criteria, see the trial protocol in Supplement 1).

Screening was performed 7 to 35 days before randomization, allowing for psychiatric assessment and medical examination, including the Mini International Neuropsychiatric Interview, version 7,^30^ MADRS interview, electrocardiography, urine samples for toxicology, and pregnancy testing. Participants also underwent blood tests and magnetic resonance imaging of the brain.

### Study Procedures

Study visits were conducted at the Northern Stockholm Psychiatric Clinic and Karolinska Institutet. The treatment protocol included 5 sessions: preparation (day −1), dosing (day 0), and 3 integrations (days 1, 8, and 15) (eAppendix 1 in Supplement 2).

Participants were randomized 1:1 (single block), using a computer-generated randomization sequence to receive psilocybin, 25 mg, or niacin, 100 mg, in identical capsules (provided by Usona Institute). To account for dropouts or protocol deviations, participants 31 to 36 were assigned to match the allocation pattern of the initial 30, ensuring balanced group sizes. Medication containers, labeled by the investigational product provider according to the randomization list, were stored in a locked cabinet and dispensed in chronological order. Only the statistician (A.B.) had access to the allocation key until study completion.

On the dosing day (7-10 hours), participants were confirmed eligible and then underwent baseline assessments, including MADRS and self-reports. The study medication was administered together with 200 mL of water. Participants were encouraged to lie down and focus inwards and offered eyeshades, music, and a light snack. Psychologists monitored safety and provided support. If required, the study physician could be called for medical assessment. Participants were monitored for 7 hours after dosing before discharge.

Study physicians conducted clinical assessments on days 8, 15, 42, and 365 after dosing, including adverse events (AEs), concomitant medication, and treatment outcomes. Participants were instructed not to disclose their assumptions about allocation. Due to the COVID-19 pandemic, follow-ups on days 42 and 365 were offered either in person or on video call, in accordance with national public health guidelines. Participants who were still clinically depressed on day 42 were referred for standard treatment. From day 42 onward, participants reported symptoms monthly online. The last study visit was scheduled for day 365.

### Efficacy Assessments

The primary outcome measure was the between-group difference in change in blinded-rater MADRS total score from baseline to day 8. Secondary outcomes were between-group differences in MADRS change from baseline to days 15, 42, and 365 and in the self-report version of MADRS (MADRS-S) from baseline to days 1 to 8, 15, and 42 and monthly through day 365, including response and remission rates. Additional secondary outcomes included changes in Clinical Global Impression^31^ scores from baseline to days 8, 15, 42, and 365 and functional disability using the Sheehan Disability Scale.^32^ Time to initiation of antidepressants was recorded on day 365. Exploratory outcomes included changes in health-related quality of life (EuroQoL 5-dimension 5-level [EQ-5D-5L] questionnaire)^33^ and anxiety using General Anxiety Disorder 7 (GAD-7)^34^ from baseline to days 8, 15, and 42 and monthly until day 365.

### Blinding, Expectancy, and Psychedelic Intensity Assessments

Blinding integrity was assessed by asking the rater on days 8 and 365 to guess treatment allocation and (on day 365 only) to rate confidence on a scale from 0 to 10, with 0 indicating not at all confident and 10 indicating very confident. Participants were asked to guess allocation and rate their confidence on day 365 only. For expectancy assessment, participants rated treatment expectation on a visual analog scale before dosing by placing a cross on a horizontal line in response to the question, “On a scale from 0 to 100, how much do you think the treatment will reduce your depression?” For psychedelic intensity assessment, participants rated the subjective intensity of their experience on a scale from 0 (not intense at all) to 10 (very intense) at the estimated peak of subjective psychedelic effects (90 minutes after dose).

### Safety Assessments

Safety was monitored by study staff from enrollment until last visit. On each visit, participants were asked if they had experienced any AE. AEs were classified for causality, severity, and seriousness. A SAE was defined as resulting in one of the following outcomes: death, inpatient hospitalization, significant or persistent incapacity, or congenital birth defect or abnormality. A treatment-emergent AE (TEAE) was any AE that occurred after administration of the study drugs; a related TEAE was a TEAE with a reasonable possibility of having been caused by the study drugs.

### Statistical Analysis

Based on prior studies, a mean decrease of 23.3 MADRS points was assumed for the psilocybin group at day 8^35^ and an improvement of 30% in the niacin group.^12^ Assuming an SD of 10, a sample size of 15 participants per group provides 80% power to detect an 11-point difference in means using a 2-group *t* test with a 2-sided *P* < .05 indicating statistical significance.

Primary, secondary, and exploratory analyses were conducted using a mixed-model repeated measures approach, including the baseline measurement as a covariate, with fixed effects for treatment, time point, and the treatment × time point interaction. An unstructured covariance matrix modeled within-subject variation, and denominator degrees of freedom were estimated using Kenward-Roger approximation. Between- and within-group differences at each time point were tested using paired *t *tests. Response (≥50% or >10-point reduction) and remission (total score <10) were assessed for MADRS and MADRS-S and compared using Fisher exact tests and univariable logistic regression. Time to initiation of antidepressants was analyzed using Kaplan-Meier estimates and log-rank tests, censoring at study discontinuation or completion. Analyses were conducted in the intention-to-treat (ITT) population, with sensitivity analyses censoring participants after antidepressant initiation or reported recreational psychedelic use. Blinding integrity, treatment expectation, and acute psychedelic intensity were summarized descriptively.

All analyses were performed using SAS, version 9.4.1 (SAS Institute Inc) or R, version 3.6.1 (R Foundation for Statistical Computing) from February 20, 2024, to June 20, 2025. For secondary and exploratory outcomes, *P* values were not adjusted for multiple comparisons. All reported group differences are based on model estimates unless otherwise specified.

## Results

### Participants

The study included 35 participants (mean [SD] age, 41.0 [10.1] years; 21 [60%] female and 14 [40%] male) diagnosed with moderate to severe recurrent MDD, with 17 randomized to the psilocybin group and 18 to the niacin group. Baseline characteristics are reported in the Table. Self-report completion remained high in both arms for 12 months. At the final MADRS-S assessment, data were available for 16 of 17 patients in the psilocybin group and 17 of 18 in the niacin group, suggesting minimal attrition bias.

**Table.** Baseline Demographic and Clinical Characteristics by Treatment Group

Characteristic	No. (%) of patients^a^	
	Psilocybin (n = 17)	Niacin (n = 18)
Demographics		
Sex		
Male	7 (41.2)	7 (38.9)
Female	10 (58.8)	11 (61.1)
Age, mean (SD), y	39.7 (10.5)	42.2 (9.9)
University degree	9 (56.2)	8 (57.1)
Employed or student	16 (100)	18 (100)
Sick leave (full or part time)	2 (11.8)	3 (16.6)
Clinical characteristics		
MADRS score, mean (SD)	21.1 (5.6)	24.6 (5.6)
Duration of current episode, mean (SD) [range], mo^b^	12.7 (10.1) [1.5-36]	13.2 (12.4) [1-48]
Lifetime depressive episodes, mean (SD) [range]^b^	8.2 (6.5) [2-25]	6.7 (5.6) [2-25]
Psychiatric inpatient admission^c^	1 (6.2)	4 (22.2)
Suicide attempt, lifetime^c^	1 (5.9)	4 (22.2)
Previous treatment		
Psychotherapy, lifetime	13 (81.2)	16 (88.9)
SSRI use, lifetime	9 (52.9)	17 (94.4)
Tapered medication before inclusion	3 (17.6)	4 (22.2)
Comorbidities		
Anxiety disorders	7 (41.2)	7 (38.9)
Neuropsychiatric disorders	1 (5.9)	3 (16.7)
Eating disorders	2 (11.8)	1 (5.6)
Stress- or trauma-related disorders	3 (17.6)	0
Chronic pain	0	3 (16.7)

Abbreviations: MADRS, Montgomery-Asberg Depression Rating Scale; SSRI, selective serotonin reuptake inhibitor.

^a^ Percentages were calculated based on available data. Data were missing for 5 patients for university degree, 1 patient for employment status, and 1 patient for psychotherapy.

^b^ Skewed distributions; ranges reported in addition to means.

^c^ Inpatient care and suicide attempts based on lifetime history.

### Efficacy

**Figure 2.**
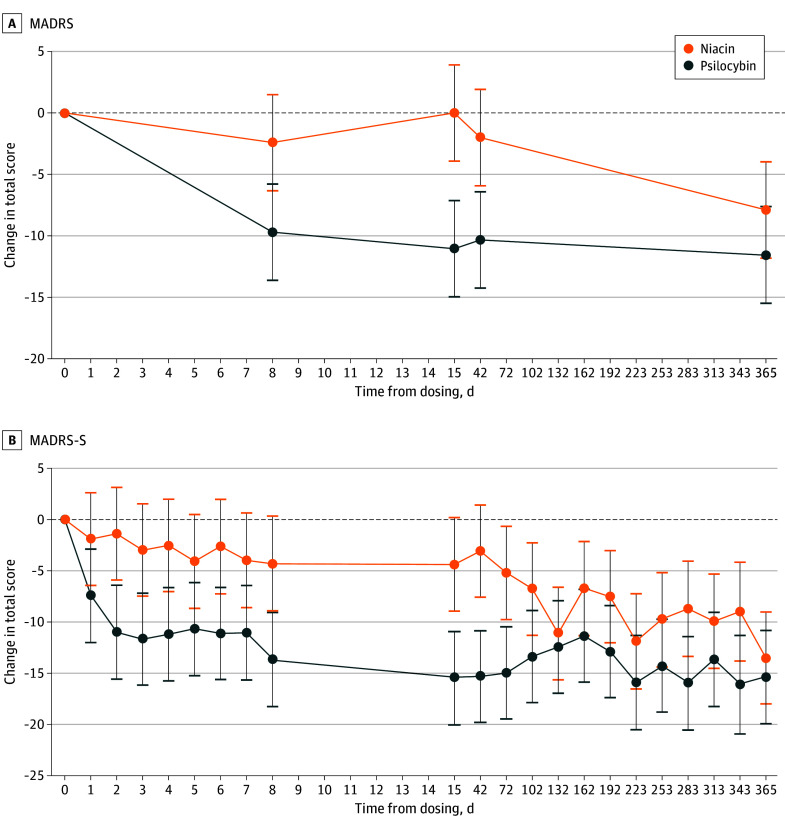
Line Graphs of Yearly Follow-Up of Depression Severity Scores A, Change in blinded rater–rated Montgomery-Åsberg Depression Rating Scale (MADRS) total score from dosing to all time points. B, Change in self-reported Montgomery-Åsberg Depression Rating Scale–Self-Report (MADRS-S) total score from dosing to all time points. Points indicate estimated marginal means; error bars indicate 95% CIs.

The study met its primary end point. On day 8, the MADRS score decreased by a mean of 7.27 points more in the psilocybin group compared with the niacin group (95% CI, −12.89 to −1.65; *P* = .01) (Figure 2A). For the secondary outcomes, the between-group difference remained significant at day 15 (mean difference, −11.03; 95% CI, −16.65 to −5.42; *P* < .001) and at day 42 (mean difference, −8.33; 95% CI, −13.94 to −2.71; *P* = .004) but no longer at day 365 (mean difference, −3.68; 95% CI, −9.30 to 1.94; *P* = .20) (Figure 2A). The psilocybin group had a significantly greater reduction in MADRS-S scores beginning on day 2 (mean difference, −9.58; 95% CI, −16.05 to −3.11; *P* = .004), with group differences persisting through day 102 (mean difference, −6.60; 95% CI, −13.01 to −0.19; *P* = .04) and then isolated effects on days 283 and 343 (Figure 2B).

**Figure 3.**
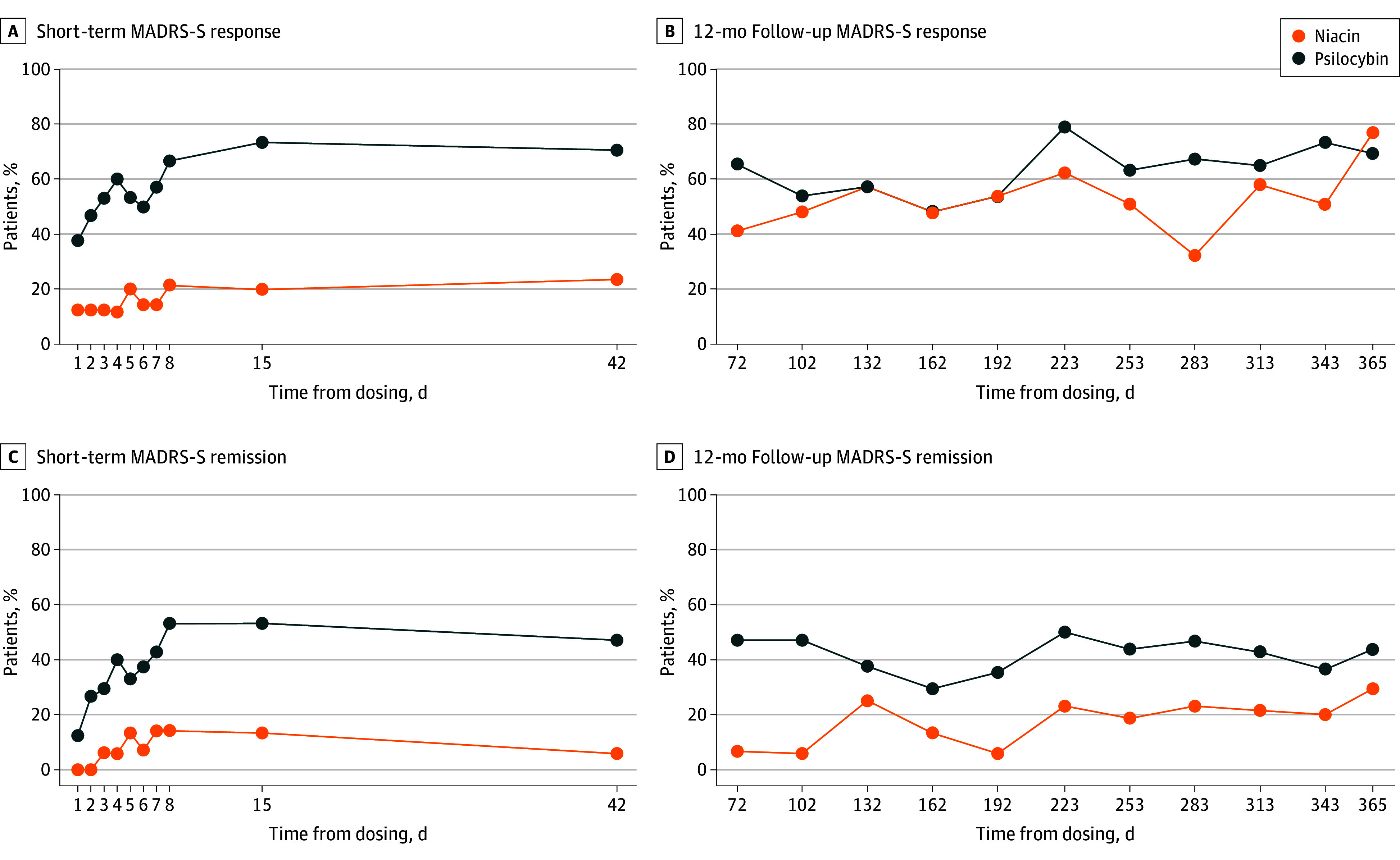
Montgomery-Åsberg Depression Rating Scale–Self-Rated MADRS-S Response and Remission: Short Term vs 12-Month Follow-up A and B, Proportion of patients meeting criteria for response on the Montgomery-Åsberg Depression Rating Scale–Self-Report (MADRS-S) during the acute phase and at 12-month follow-up, respectively. C and D, Corresponding proportions meeting criteria for remission during the short-term phase and at 12-month follow-up, respectively.

For remission at day 8, the psilocybin group (n = 8) had an odds ratio (OR) of 4.00 (95% CI, 0.85-29.17) relative to the niacin group (n = 2), increasing to 10.00 (95% CI, 1.64-194.00) at day 15 (n = 10 vs 2) and 9.00 (95% CI, 1.45-175.40) at day 42 (n = 9 vs 1); the OR was 1.29 (95% CI, 0.39-4.36) at day 365 (n = 9 vs 7). At day 42, the remission rates were 52.9% (9 of 17) for psilocybin vs 5.9% (1 of 17) for niacin. We found a 365-day remission rate (MADRS) for psilocybin of 52.9% (9 of 17) for the ITT population, which was not significantly different from the 41.2% (7 of 17) remission rate observed for niacin. Response and remission rates based on MADRS-S at each time point are presented in Figure 3A-D.

**Figure 4.**
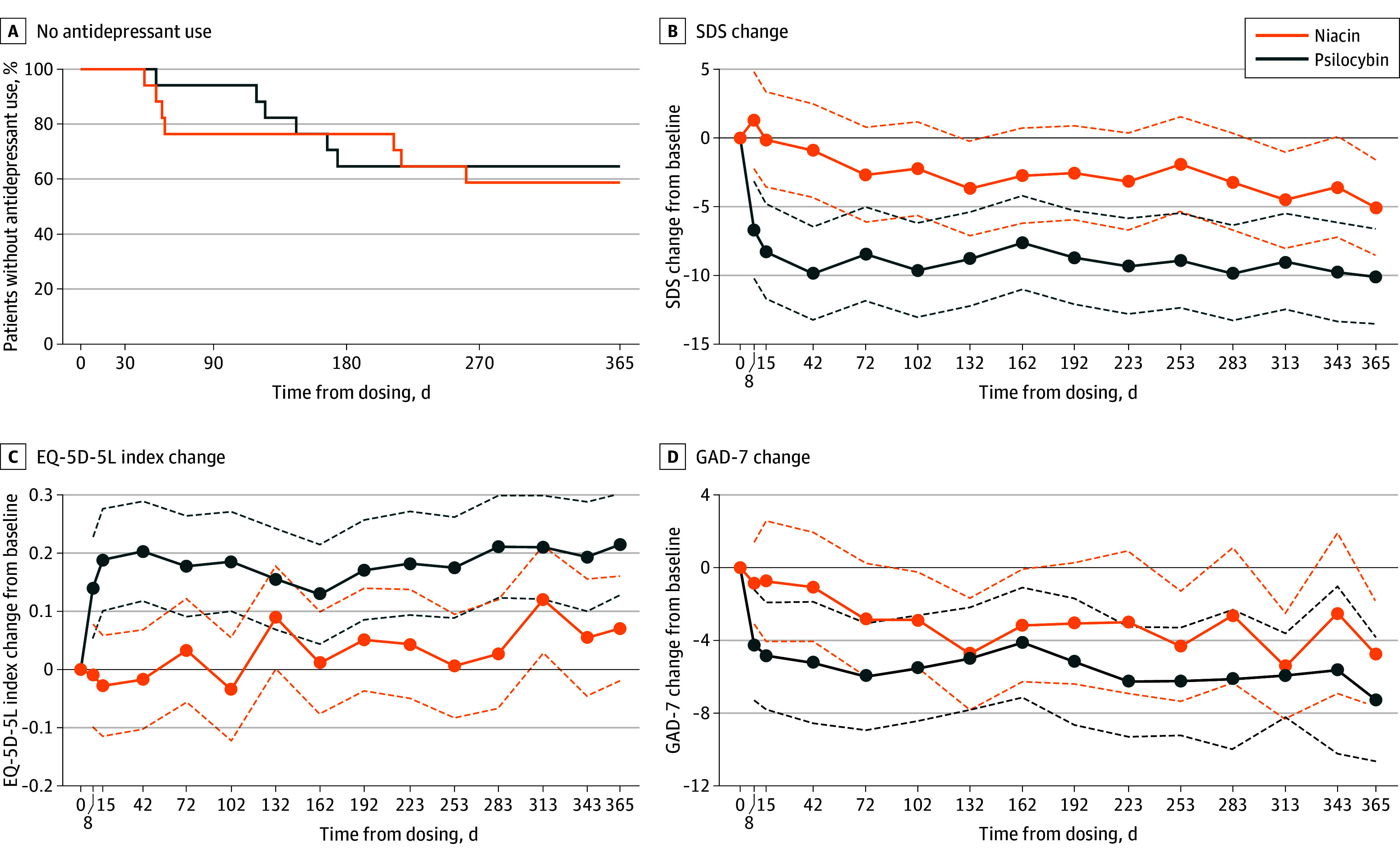
Line Graphs of Antidepressant Use and Patient-Reported Outcomes During 12-Month Follow-Up A, Kaplan-Meier curves by treatment group, showing the proportion of participants without antidepressant use during the 12-month follow-up. B, Change from baseline in Sheehan Disability Scale (SDS) scores during follow-up by treatment group, displayed as model-estimated means with 95% CIs (dashed lines). C, Change in EuroQoL 5-dimension 5-level (EQ-5D-5L) index scores during follow-up by treatment group, displayed as model-estimated means with 95% CIs (dashed lines). D, Change in Generalized Anxiety Disorder 7 (GAD-7) scores during follow-up by treatment group, displayed as model-estimated means with 95% CIs (dashed lines).

Antidepressant initiation during follow-up did not significantly differ between groups, with 6 participants in the psilocybin group and 7 in niacin group starting treatment. Among these, the mean (SD) time to antidepressant initiation was 130.0 (44.6) days in the psilocybin group vs 128.0 (96.8) days in the niacin group (*P* = .70) (Figure 4A). Additional outcomes, including Sheehan Disability Scale, EQ-5D-5L, GAD-7, Clinical Global Impression, and response rates, are reported in Figure 4B-D and eAppendix 2 and eTables 1 to 13 in Supplement 2.

### Sensitivity Analyses

Two sensitivity analyses were conducted alongside the ITT analysis for MADRS and MADRS-S, censoring participants from the time of antidepressant initiation (n = 6 in the psilocybin group and 7 in the niacin group; all after day 42) or from reported psychedelic use (n = 2 in the niacin group, 1 participant before and 1 after day 42). Across all analyses, significant between-group differences in change of MADRS scores were observed through day 42 but not for day 365. For MADRS-S, censoring on antidepressant initiation resulted in a slightly shorter duration of effect (day 72) than for ITT (day 102) (eTable 1 in Supplement 2).

### Blinding Integrity

On day 365, 16 of 17 participants (94.1%) in the psilocybin correctly guessed allocation (mean [SD] confidence, 9.53 [0.80] on a 10-point scale, with 0 indicating not at all confident and 10 indicating very confident), whereas in the niacin group the rate was 16 of 16 (100%) (mean [SD] confidence, 9.00 [1.75]); however, 1 participant did not fill in the form . The blinded rater on day 8 correctly guessed the assignment of 12 of 17 (70.6%) in the psilocybin group and 15 of 17 (88.2%) in the niacin group; confidence was not measured on this time point. The blinded rater on day 365 correctly guessed 14 of 17 (82.4%) (mean [SD] confidence, 7.94 [1.71]) in the psilocybin group and 13 of 17 (76.5%) (mean [SD] confidence, 7.88 [1.93]) in the niacin group.

### Treatment Expectation and Psychedelic Intensity

On a scale of 0 to 100, with 0 indicating no expectation of improvement and 100 indicating the highest possible expectation of improvement, the mean (SD) treatment expectation score was 56.7 (22.3) in the psilocybin group and 56.8 (27.5) in the niacin group. The mean (SD) psychedelic intensity in the psilocybin group was 7.06 (3.58), ranging from 0 to 10, with 0 indicating not intense at all and 10 indicating very intense, whereas the mean (SD) in the niacin group was 1.22 (1.63), ranging from 0 to 5 on the same scale.

### Safety

The most common TEAEs (with causality classified as certain, probable or likely, or possible) reported during the follow-up period in the psilocybin group were headache (n = 9), anxiety (n = 7), hallucination (n = 5), agitation (n = 3), hypertension (n = 3), and paresthesia (n = 3). In the niacin group, the most common TEAEs were headache (n = 5), anxiety (n = 3), and paresthesia (n = 2). Ten SAEs were reported; none of these were considered related to psilocybin. Notably, 2 participants in the psilocybin group reported anxiety of severe grade that persisted weeks after dosing (eTable 2 in Supplement 2)

## Discussion

Psilocybin generated a significantly larger and clinically meaningful^36^ decrease in depressive symptom severity at the primary end point (MADRS score of day 8) compared with niacin. This finding is consistent with previous RCTs but now for the first time, to our knowledge, demonstrated as a primary outcome.^9,37^ Self-reported MADRS-S data supported significant improvement already at day 2, corroborating the rapid antidepressive effect reported in previous trials.^9,12^ At early time points, antidepressant effects were numerically larger in the self-rated than in the clinician-rated MADRS. Such rapid effects are comparable only to those of ketamine^38^ and possibly electroconvulsive therapy^39^ and faster than other MDD treatments, including SSRIs, cognitive behavioral therapy, and repeated transcranial magnetic stimulation.^40,41,42^

At 6 weeks, the remission rate was 52.9% for psilocybin vs 5.9% for niacin. Placebo-controlled SSRI and serotonin-norepinephrine reuptake inhibitor trials have reported remission rates of approximately 40% to 45% with active treatment vs approximately 30% for placebo,^43^ suggesting a lower placebo effect in this trial than is typical for trials of conventional antidepressants.^44,45,46^

The 1-year placebo-controlled follow-up retained all but 1 participant. Psilocybin remained superior to niacin at 6 weeks but not at 12 months for MADRS. Self-reports indicated superiority for 102 days, with significant differences in both change and remission rates. Censoring participants who initiated antidepressants shortened the duration of effect to 72 days, perhaps reflecting the smaller sample size. This finding aligns with a recent 12-month follow-up of a small, nonrandom sample from the original cohort of patients with treatment-resistant depression, reporting a median time to depressive event of 92 days in the 25-mg psilocybin group.^13^

We found a 12-month remission rate (MADRS) of 52.9% for psilocybin in the ITT population, which is comparable to the 58.0% reported in an open-label follow-up. This finding was not significantly different from the 41.2% remission rate observed for niacin, but whereas psilocybin maintained a high degree of remission from day 8 to the end of the study, the remission rate in the niacin group increased from 11.8% to 41.2% during the same period. A sensitivity analysis censoring participants on antidepressant initiation suggested that antidepressants did not drive the improvement in the placebo group, underscoring the episodic nature of MDD,^47^ with potential for spontaneous remission that may cause uncontrolled studies to overestimate durability of treatment effects. In addition, the contribution of other interventions (eg, psychotherapy) cannot be excluded.

Antidepressant initiation occurred in 6 of the 17 participants randomized to the psilocybin group, similar to previous reports^18,48^ and not significantly differing from the niacin group (7 of 18 participants). Time to relapse after psilocybin treatment appears similar to that reported after discontinuation of SSRI treatment, cognitive behavioral therapy, repeated transcranial magnetic stimulation, or electroconvulsive therapy.^40,41,42,49^ Secondary outcomes were broadly consistent with sustained symptomatic improvement, including health-related quality of life and functional disability, which favored psilocybin (eAppendix 2 and eTables 1 to 13 in Supplement 2).

Our findings indicate that psilocybin might be a valuable addition to current treatments because of its rapid onset and relatively long-lasting effects, although the duration may not be as long as suggested by previous uncontrolled studies.^18,19^ Repeated dosing or maintenance therapy might therefore be needed to prevent relapse.

Psilocybin was generally well tolerated, producing mostly transient TEAEs of mild to moderate severity. However, 2 participants reported severe, persisting anxiety that required medical attention, indicating that some patients may need additional support, aligning with findings from another study.^50^

Psychedelic intensity ratings clustered at the upper end of the scale in the psilocybin group and at the lower end for the niacin group, suggesting a high likelihood of functional unblinding. Indeed, blinding integrity was low for both groups. Participants correctly guessed allocation in 94.1% of cases in the psilocybin group vs 100% for niacin and the blinded rater in 82.4% vs 76.5%. We conclude that using niacin as an active placebo did not achieve the blinding integrity generally expected from a double-blind RCT, even in a psychedelic-naive population.

Overall treatment expectation indicated a moderately positive belief in treatment efficacy in our self-selected sample, raising the possibility of an expectancy-blinding interaction.^20^ As treatment expectation is known to influence clinical outcomes,^51,52,53^ disentangling direct pharmacologic effects from indirect effects related to expectancy, subjective psychedelic experience, and functional unblinding remains an important next step in the field.

### Limitations

This study has some limitations. One limitation was the modest sample size, increasing the likelihood of chance imbalances at baseline, as indicated by the higher prevalence of prior SSRI use in the niacin group. Although sufficiently powered to answer the primary research question, the study was not designed to yield conclusions regarding secondary outcomes, such as long-term effects. Recruiting participants in social media might have introduced self-selection bias and expectancy confounds. Although community recruited, participants had a well-documented history of depression. The low blinding integrity in participants and raters might have influenced the treatment effect size, particularly given the subjective nature of the outcome measures, with self-reports potentially more sensitive than clinician-rated scales.^21,54^ Additionally, using study physicians as blinded raters may have compromised blinding integrity

## Conclusions

In this RCT, a single dose of psilocybin, 25 mg, generated a clinically meaningful reduction in depressive symptoms, already at day 8, compared with 100 mg of niacin. This finding implies that psilocybin can be an option to standard treatments when rapid symptom relief is important. Superiority over niacin persisted on day 42 and was observed until day 102 in the self-ratings but no longer. This study adds nuance to previous reports^18,19^ of antidepressant effects beyond 6 months in uncontrolled trials, indicating a potential requirement for a booster dose or maintenance therapy to prevent relapse. However, larger-sample RCTs are needed to draw firm conclusions about long-term effects. Psilocybin was well tolerated by most participants with mostly transient, mild to moderate AEs and no related SAEs. Two participants reported severe and persisting anxiety, indicating that some individuals may require additional support. The reported low blinding integrity highlights the limitations of niacin as an active placebo, also in a psychedelic-naive population. Further research is necessary to refine study design in psychedelic RCTs, to address the issue of functional unblinding, and to account for expectancy and placebo effects.

## Notes

Supplement 1.
Trial Protocol

Supplement 2. **eAppendix 1. **Psychotherapeutic Support in the PSIPET Study **eAppendix 2. **Supplemental Results **eTable 1.** MADRS (Clinician-Rated) and MADRS-S (Self-Report) (Days 1–365) **eTable 2.** Treatment-Emergent Adverse Events (TEAEs) and List of Treatment Emergent SAEs Reported Throughout the Complete Follow-Up **eTable 3.** Full-Model Estimates of the Between-Group Difference in Change From Baseline in MADRS Total Score to All Timepoints
**eTable 4.** Full-model Estimates of the Between-Group Difference in Change From Baseline in MADRS-S Total Score to All Timepoints **eTable 5.** Full-model Estimates of the Between-Group Difference in Change From Baseline in GAD-7 Total Score to All Timepoints **eTable 6.** Full-model Estimates of the Between-Group Difference in Change from Baseline in SDS Total Score to All Timepoints **eTable 7.** Full-Model Estimates of the Between-Group Difference in Change From Baseline in EQ5D-5L (Index) to All Timepoints **eTable 8.** Full-Model Estimates of the Between-Group Difference in Change From Baseline in CGI-S to All Timepoints **eTable 9.** Full-Model Estimates of the Between-Group Difference in Change From Baseline in CGI-I to All Timepoints **eTable 10.** MADRS Response Rates **eTable 11.** MADRS Remission Rates **eTable 12.** MADRS-S Response Rates **eTable 13.** MADRS-S Remission Rates

Supplement 3.
Data Sharing Statement
