# Supplementary material for: Short-Term and Late-Term Effects of Psilocybin on Symptoms in Major Depression: A Randomized Clinical Trial
Source: JAMA Netw Open. 2026 May 15;9(5):e2612589. doi: 10.1001/jamanetworkopen.2026.12589 (PMC13179547; doi:10.1001/jamanetworkopen.2026.12589)
Supplement: Supplement 1. — Trial Protocol [file jamanetwopen-e2612589-s001.pdf]

The Effect of Psilocybin on MDD Symptom Severity and Synaptic Density – A Single Dose Randomized, Double Blind, Placebo-Controlled Phase 2 Positron Emission Tomography Study

Effekten av psilocybin på depressionssymptom och synaptisk densitet – en singeldos, randomiserad, dubbel-blind, placebokontrollerad, fas två, positron-emissionstomografistudie

Sponsor:
SLSO
Organisation number: 232100-0016

Sponsor Representative:
Andreas Carlborg, MD, PhD
Head of clinic
Norra Stockholms Psykiatri, SLSO
Vårdvägen 3
112 19 Stockholm
Sweden

Principal Investigator (PI):
Johan Lundberg, associate professor, MD, PhD
Head of section for affective disorders
Section for affective disorders, Norra Stockholms Psykiatri, Region Stockholm
Center for Psychiatry Research, Department of Clinical neuroscience, Karolinska Institutet and Region Stockholm

Co-PIs:
Maria Beckman, clinical psychologist, PhD
Center for Psychiatry Research, Department of Clinical neuroscience, Karolinska Institutet and Region Stockholm

Mikael Tiger, MD, PhD
Senior physician
Section for affective disorders, Norra Stockholms Psykiatri, Region Stockholm
Center for Psychiatry Research, Department of Clinical neuroscience, Karolinska Institutet and Region Stockholm

Co-investigators:
Carl-Johan Ekman, MD, PhD
Senior physician
Section for affective disorders, Norra Stockholms Psykiatri Region Stockholm
Center for Psychiatry Research, Department of Clinical neuroscience, Karolinska Institutet and Region Stockholm

Eva Hejne, associate professor, MD, PhD
Senior physician
Department of Clinical Sciences, Child and Adolescent Psychiatry, Umeå universitet

Alexander Lebedev, assistant professor, MD, PhD
Department of Clinical neuroscience, Karolinska Institutet

Predrag Petrovic, associate professor, MD, PhD
Senior physician
Section for psychosis, Norra Stockholms Psykiatri, Region Stockholm, Department of Clinical neuroscience, Karolinska Institutet

Hampus Yngve, MD
Norra Stockholms Psykiatri Region Stockholm

EudraCT-number: 2020-002790-94

Sponsor

Date: 11 juni 2020

By:

Print Name: Andr as Carlborg
Title: Head of Clinic/verksamhetschef; MD PhD

Investigator

Date: 10 JUNE 2020

By:

Print Name: Johan Lundberg
Title: MD PhD

Table of Contents

1. Contact Information for Study Personnel 10
2. Abbreviations 11
3. PROTOCOL SYNOPSIS 13
4. INTRODUCTION 21
   - 4.1 Acknowledgement 21
   - 4.2 Purpose 21
   - 4.3 Study rationale 21
   - 4.4 Background 21
     - 4.4.1 Major depressive disorder (MDD) 21
     - 4.4.2 Psilocybin 23
     - 4.4.3 Previous Clinical Experience with Psilocybin Relevant to MDD 23
   - 4.5 The Set and Setting (SaS) Protocol 24
5. OBJECTIVES AND ENDPOINTS 25
   - 5.1 Primary objective 25
     - 5.1.1 Primary study endpoint 25
     - 5.1.2 Secondary endpoints 26
   - 5.2 Secondary objective and endpoint 26
     - 5.2.1 Secondary endpoints 27
   - 5.3 Exploratory Objectives and endpoints 27
   - 5.4 Safety Objectives 27
     - 5.4.1 Specific Safety Monitoring Objectives 28
     - 5.4.2 Safety Endpoints 28
   - 5.5 Outcome Measures 28
     - 5.5.1 Montgomery-Asberg Depression Rating Scale (MADRS) 28
     - 5.5.2 Sheehan Disability Scale (SDS) 29
     - 5.5.3 General Anxiety Disorder 7 (GAD-7) 29
     - 5.5.4 Clinical Global Impression – Improvement and Severity (CGI-I and CGI-S) . 30
     - 5.5.5 EQ-5D 30
     - 5.5.6 Challenging Experience Questionnaire (CEQ) 31
     - 5.5.7 Mystical Experience Questionnaire (MEQ-30) 31
     - 5.5.8 Emotional Breakthrough Inventory (EBI) 31
     - 5.5.9 Treatment Expectation 31
     - 5.5.10 Treatment Evaluation 32
     - 5.5.11 Acceptance in Action Questionnaire (AAQ-II) 32
     - 5.5.12 Actively Living and Interconnecting Vitally in one's Embedded-world (ALIVE) 32

5.5.13 Meaning of Life Questionnaire (MLQ) 33
5.5.14 Satisfaction with Life Scale (SWLS) 33
5.5.15 Working Alliance Inventory-Short (WAI-S) 33
5.5.16 The Lasting Effects Questionnaire (LEQ) 33
5.5.17 Manual for conducting semi-structured interviews 34
5.5.18 Motivational Interviewing Treatment Integrity Code (MITI) 34
5.5.19 Scale for Psychedelic Intensity Rating (SPIR) 34
5.5.20 The Client Language Easy Rating (CLEAR) 34
5.6 Eligibility and Safety Measures 35
5.6.1 The MINI Structured Interview for ICD-10 diagnosis 35
5.6.2 Questionnaire Order of Assessments 35
5.6.3 Laboratory Assessments 35
6. PROTOCOL DESIGN 35
6.1 Overall Study Design 35
6.2 Justification for Selected Aspects of the Study Design 36
6.2.1 Use of a single psilocybin dose 36
6.2.2 Psilocybin and Niacin Dosing 37
6.3 Planned Duration of Study 37
7. PARTICIPANT POPULATION 39
7.1 Inclusion and Exclusion Criteria 39
7.1.1 Inclusion Criteria 39
7.1.2 Exclusion Criteria 40
7.2 Justification for Inclusion/Exclusion Criteria 42
7.2.1 Outside Psychotherapy 42
7.2.2 Benzodiazepines 42
7.2.3 Age range 43
7.2.4 Medically Healthy Participants 44
7.2.5 Psychiatric Symptoms 44
7.2.6 Medication Exclusions 45
7.2.7 Blood Pressure 45
8. SCREENING PROCESS AND PROCEDURES 45
8.1 Recruitment 45
8.2 Study Recruitment Website and Pre-Screener Questionnaire 46
8.3 Telephone Screen 46
8.4 Written Informed Consent 46
8.5 In-Person Screening Visit (7-35 days prior to dose) 47

8.5.1 Inclusion/ Exclusion Criteria Confirmation 48
8.5.2 Magnetic Resonance Imaging (MRI) 48
8.5.3 Lumbar puncture 48
8.5.4 PET1 48
9. PREPARATION, RANDOMIZATION, DOSING AND FOLLOW UP PROCEDURES 48
9.1 Preparatory Sessions with Clinical Facilitators (1 day prior to dose) 48
9.1.1 Post Preparatory Session Assessment (1 day prior to dose) 49
9.2 Randomization 49
9.3 Day 0: Dosing Session 49
9.3.1 Pre-Dose 49
9.3.2 Dosing 50
9.3.3 Post-Dosing Release Procedures 54
9.4 Day 1: Post Dose, Integration Session #1 55
9.4.1 Day 1 MRI (+3 days) 55
9.5 Day 8 (-1/+2 days) and 15 (+2 days) 55
9.5.1 Day 15 post dose PET2 and biomarker sampling (±7 days). 55
9.5.2 Day 8 and 15 Integration Sessions #2 and #3 55
9.6 Day 42: Post Dose (±3 days) 55
9.7 Monthly evaluations, visits from day 43 to 365 and 56
9.8 Day 360 Post dose (±14 days) End of study visit 56
9.9 Add on antidepressant treatment 56
9.10 Blinding Related to Delivery of Study Interventions and Collection of Outcome Data 56
9.10.1 Unblinding Procedure 57
9.11 Early Termination Visit 57
9.12 Unscheduled Visits 57
10. STUDY DISCONTINUATION AND COMPLETION CRITERIA 57
10.1 Screen Failures 57
10.2 Evaluable Participants 58
10.3 Early Termination from the Study 58
10.4 Early Termination Post-Baseline and Pre-Randomization (Preparation Phase Termination) 58
10.4.1 Early Terminations Post-Randomization and Pre-Dosing 58
10.4.2 Early Termination Post Dosing 58
10.4.3 Dropouts 59
10.4.4 Lost to Follow-up Post Dosing 59
10.4.5 End of Study Definition 59

10.4.6 Premature Study Discontinuation 59
11. INVESTIGATIONAL PRODUCTS 60
11.1 Description of Investigational Products 60
11.1.1 Psilocybin 60
11.1.2 Description of Active Placebo 60
11.2 Source 60
11.2.1 Study Drugs Administered 60
11.3 Dosing 61
11.3.1 Drug Delivery, Storage, Handling, and Accountability 61
12. PET data acquisition 61
12.1 [11C]UCB-J 62
12.2 PET Image analysis 62
13. Peripheral biomarkers sampled at time of PET1 and PET2 62
14. Sampling and analysis of cerebrospinal fluid 63
15. Magnetic Resonance Imaging (MRI) experimental procedure and data aquisition 63
15.1 fMRI tasks 63
15.2 MRI safety 65
16. Statistical analysis 65
16.1 Power Analysis and Sample Size Determination 65
16.2 Populations for Analyses 66
16.3 Statistical Hypothesis 66
16.4 Statistical Analyses 66
16.4.1 Efficacy Analyses 67
16.4.2 Multiple comparisons 67
16.4.3 Handling of missing data 67
16.4.4 Interim analyses 68
16.4.5 Subgroup Analyses 68
16.4.6 Safety Analyses 68
17. SAFETY MANAGEMENT 68
17.1 Risks Associated with Psilocybin 68
17.1.1 Physiological and Psychological Adverse Effects 68
17.1.2 Visual Perceptual Effects 69
17.1.3 Drug Interactions 69
17.1.4 Risk of Worsening MDD 69
17.2 Risks Associated with Collection of Potentially Sensitive Information 70
17.3 Risks Associated with Recordings of Sessions 70

17.4 Risks Associated with Psychiatric Questionnaires 71
17.5 Risks Associated with venepuncture and arterial cannulation 71
17.6 Risks Associated with lumbar puncture 71
17.7 Risks Associated with Positron Emission Tomography (PET) 71
17.8 Contacting Emergency Services 72
17.9 Abuse Liability 72
17.9.1 Abuse Monitoring 73
18. ADVERSE EVENTS 73
18.1 Definitions 73
18.1.1 Adverse Event 73
18.1.2 Treatment Emergent Adverse Events 74
18.1.3 Solicited Adverse Events 74
18.1.4 Serious Adverse Events 75
18.1.5 Definition of Terms 75
18.2 Guidelines for Assessing Intensity of an Adverse Event 76
18.3 Guidelines for Determining Causality of an Adverse Event 76
18.4 Actions to be Undertaken 76
18.5 AE Collection Period and Follow up 76
18.6 Regulatory Reporting 76
18.6.1 Definition of reason to stop the trial 77
18.6.2 End of Trial Notification 77
19. STUDY MONITORING, AUDITING AND DOCUMENTATION 77
20. HUMAN SUBJECTS PROTECTIONS 78
20.1 Study Conduct 78
20.2 Principal Investigator Responsibilities 78
20.3 Human Subjects Training 78
20.4 Voluntary Participation 78
20.5 Benefits of Participation 79
20.6 Alternatives to Participation 79
20.7 Study and Site Closure 79
20.8 Vulnerable Populations 79
20.9 Cost to Participants 79
20.10 Participant Compensation 80
20.11 Treatment/ Compensation for Study Related Injury 80
20.12 Protocol Deviations and Violations 80
20.13 Record Retention 80

20.14 Publication Policy ... 80
21. CONFIDENTIALITY AND DATA SECURITY ... 81
21.1 Confidentiality and Data Security at Research Site ... 81
21.2 eCRF ... 81
21.3 Take Care and 1177 ... 81
22. PREGNANCY ... 81
22.1 Definitions: Women of Childbearing Potential (WOCBP) ... 81
22.2 Contraception Guidance ... 82
22.2.1 Male participants ... 82
22.2.2 Female participants ... 82
22.3 Pregnancy Testing ... 83
23. Previous experience ... 83
24. Substantial amendments ... 84
25. Appendixes ... 84
26. REFERENCES ... 84

1. CONTACT INFORMATION FOR STUDY PERSONNEL

Johan Lundberg, MD, PhD; 08-12348784; johan.lundberg@ki.se
Role: Principal investigator (PI)

Maria Beckman, clinical psychologist, PhD; 070-797 22 65; maria.beckman@ki.se
Role: Co-PI and responsible set and settings protocol

Mikael Tiger, MD, PhD; 08-1234 00 00; mikael.tiger@ki.se
Role: Co-PI

Carl-Johan Ekman, MD PhD; 08-1234 00 00; carl-johan.ekman@sll.se
Role: Co-investigator and Study Physician

Hampus Yngve, MD, 08-1234 00 00; hampus.yngve@sll.se
Role: Study Physician

Opokua Britton Cavaco; 08-517 744 22; opokua.britton.cavaco@ki.se
Role: Research nurse, PET center, Karolinska Institutet; PET measurements

Johanna Evengård; 08-123 484 33; johanna.evengård@sll.se
Role: research nurse, Norra Stockholms Psykiatri, SLSO

Eva Henje, MD, PhD; 073-801 92 12; eva.henje@umu.se
Role: Co-investigator and responsible set and settings protocol

Alexander V. Lebedev MD, PhD; 076-59 04 649; alexander.lebedev@ki.se
Role: *Co-investigator and responsible MRI arm*

Predrag Petrovic, MD, PhD; 073-51 01 211; predrag.petrovic@ki.se
Role: *Co-investigator and responsible MRI arm*

Åsa Carlsheimer, Senior Biostatistician; asa.carlsheimer@4pharma.com
Role: *statistician*

Jack Spira, monitor; jack.spira@telia.com
Role: *monitor*

2. ABBREVIATIONS

21 CFR	Title 21 of the Code of Federal Regulations
AAQ-II	Acceptance in Action Questionnare
AE	Adverse Event
AUDIT	Alcohol Use Disorders Identification Test
BDI	Beck Depression Inventory
BP	Binding Potential
CBC	Complete Blood Count
CGI-I	Clinical Global Impression - Improvement
CIOMS	Council for International Organizations of Medical Sciences
CLEAR	Client Language Easy Reading
CRF	Case Report Form
d	Cohen's d
DBS	Deep Brain Stimulation
DUDIT	Drug Use Disorders Identification Test
EBI	Emotional Breakthrough Inventory
ECG	Electrocardiogram
ECT	Electroconvulsive Therapy
EDC	Electronic Data Capture
EQ-5D	Euro Qual 5 Dimensions
FDA	Food and Drug Administration
FSH	Follicle Stimulating Hormone
g	Hedges g
GCP	Good Clinical Practice
HADS	Hospital Anxiety and Depression Scale
HAM-D	Hamilton Depression Scale
HDPE	High-Density Polyethylene
Hopkins	Johns Hopkins University
HPMC	Hydroxypropyl Methylcellulose
HPPD	Hallucinogen-Persisting Perception Disorder
HR	Heart Rate
Hrs	Hours
HRT	Hormonal Replacement Therapy
ICD-10	International Classification of Disorders - 10
ICF	Informed Consent Form
ICH	International Conference on Harmonization
IND	Investigational New Drug
IP	Investigational Product
IRB	Institutional Review Board
ISF	Investigator Site File
IUD	Intrauterine Device
IUS	Intrauterine Hormone-releasing System
LEQ	Lasting Effects Questionnaire
LSD	Lysergic Acid Diethylamide
MADRS	Montgomery-Asberg Depression Rating Scale
MADRS-S	Montgomery-Asberg Depression Rating Scale- Self rating

MAOIs	Monoamine Oxidase Inhibitors
MBq	Mega-Becquerel
MD	Medical Doctor
MDD	Major Depressive Disorder
MDMA	3,4-Methylenedioxy-methamphetamine
MedDRA	Medical Dictionary for Regulatory Activities
MEQ-30	Mystical Experience Questionnaire
mmHg	Millimeters of Mercury
MMRM	Mixed Model for Repeated Measures
MPA	Medical Products Agency
MRI	Magnetic Resonance Imaging
MRS	Magnetic Resonance Spectroscopy
NYU	New York University
p	Probability
PCP	Phencyclidine
PET	Positron Emission Tomography
PI	Principal Investigator
ROI	Region(s) of Interest
SAE	Serious Adverse Event
SAEs	Serious Adverse Events
SAP	Statistical Analysis Plan
SaS	Set and Setting Protocol
SDS	Sheehan Disability Scale
SNRIs	Serotonin-norepinephrine reuptake inhibitors
SOP	Standard Operating Procedure
SPIR	Subjective Psychedelic Intensity Rating
SSRIs	Selective Serotonin Reuptake Inhibitors
SUSAR	Suspected Unexpected Serious Adverse Reaction
TAC	Time Activity Curve
TCAs	Tricyclic Agents
TEAE	Treatment Emergent Adverse Event
THC	Tetrahydrocannabinol
TIA	Transient Ischemic Attack
TMS	Transcranial Magnetic Stimulation
TRD	Treatment-Resistant Depression
TSH	Thyroid Stimulating Hormone
VNS	Vagus Nerve Stimulation
WOCBP	Woman of Childbearing Potential

3. PROTOCOL SYNOPSIS

Title
The effect of psilocybin on MDD symptom severity and synaptic density – a single dose randomized, double blind, placebo-controlled phase 2b positron emission tomography study

Study Code
PSIPET

Name of Sponsor
SLSO
Organisationsnr: 232100-0016

Sponsor representative:
Andreas Carlborg
Norra Stockholms Psykiatri
Vårdvägen 3
112 19 Stockholm
Sweden

Medical Monitor
Inspira Medical AB

Phase of Study
Phase 2b

Sample Size
30 randomized

Name of Investigational Product (IP)
Psilocybin, 3-[2-(dimethylamino)ethyl]-1H-indol-4-yl] dihydrogen phosphate

Name of Active Placebo
Niacin

EudraCT
2020-002790-94

Description of IP and Active Placebo

**Study Intervention Name:**	Psilocybin (active drug product)	Niacin (active placebo product)
**Dosage formulation:**	One active capsule contains 25 mg of psilocybin	One active placebo capsule contains 100 mg of niacin
**Capsule:**	Size 2 hydroxypropyl methylcellulose (HPMC), opaque	Size 2 HPMC, opaque
**Unit dose strength:**	25 mg	100 mg
**Route of Administration:**	Oral (solid dose)	Oral (solid dose)
**Dosing instructions:**	One capsule administered with water	One capsule administered with water
**Packaging and Labeling:**	Study Intervention will be provided in a high-density polyethylene (HDPE) bottle. Each bottle will contain one capsule (psilocybin or niacin) and will be labeled as required per Swedish requirement for blinded study.	

Study Description and Overview

Thirty participants (males and females) ages 20 to 65 inclusive, who, at Screening, meet ICD-10 criteria for major depressive disorder (MDD), have a current depressive episode of at least 30-day duration, have a Screening Montgomery-Asberg Depression Rating Scale (MADRS) total score ≥ 22 and meet all other inclusion/exclusion criteria will be randomized with a 1-to-1 allocation under double-blind conditions to receive a single 25 mg oral dose of psilocybin or a single 100 mg oral dose of niacin. Niacin will serve as an active placebo control that provides an acute physiological response (flushing) that is intended to aid in blinding of intervention allocation. All randomized participants will be included in the Full Analysis Set (FAS) population used in analyzing primary and secondary study endpoints.

Only participants who meet depressive symptom severity criteria at web screening (MADRS self-rating (MADRS-S) score ≥ 19) and who do not show an unacceptably large degree of symptom improvement between the web screening and in-person screening (indexed by change in MADRS-S (improvement) ≤ 30% (MADRS representing web screening will be approximated to MADRS-S + 3) will be eligible for randomization. This is to minimize the risk for spontaneous remission before dosing.

Participants deemed eligible following successful completion of all screening assessments including a structural Magnetic Resonance Imaging (MRI) examination will be determined as eligible.

Eligible participants at Baseline will submit cerebrospinal fluid (CSF), submit blood samples, be examined with positron emission tomography (PET) and the radioligand [$_{11}$C]UCB-J and receive one preparation session (see further below) to be eligible for randomization on Dosing (Day 0) to receive either psilocybin or niacin active-placebo. They will complete follow-up visits on study Day 1, 8, 15, 42 and 365 (within corresponding visit windows). At day 15 the sampling of CSF, blood and [$_{11}$C]UCB-J PET will be repeated.

After day 42, all participants will be given follow-up visits at Norra Stockholms Psykiatri for up to one year after dosing, to study dedicated physicians or nurses at a frequency determined by the health care professional. If needed to reach/stay in remission, the participants will be provided antidepressant treatment in accordance with the regional guidelines for antidepressant treatment (https://psykiatristod.se/regionala-vardprogram/depression). At least monthly the participants will be asked to provide on-line symptom rating data (via 1177.se). At the 365-day visit, symptoms will be evaluated using MADRS, CGI-I and CGI-S. After completing the study (one year or withdrawal), participants will be subject to standard care, including referral in accordance with regional guidelines.

The study outcome measures will be used to assess depressive symptoms, clinical global functioning, functional disability, anxiety symptoms and health-related quality of life. Safety outcome measures will be collected at all assessment time points from the time of consent through the end of study.

To enhance participant safety, the current study proposes to test psilocybin within a "set and setting" (SaS) protocol similar to the protocol that has been used in all modern studies of psilocybin in both diseased and normal healthy populations. The SaS protocol for this study includes: 1) a preparation with session Facilitators (licensed psychologists) prior to dosing; 2) administration of study medications in an aesthetically neutral room under the supervision of two Facilitators who are present throughout the session (with the exception of short, temporary allowances for facilitator breaks; e.g. bathroom breaks); and 3) three post-dose integration sessions during which participants are encouraged to discuss their intervention experience with the Facilitators. To evaluate the Facilitators' adherence to the study manual, and the role of Facilitators' and participants' in-session behaviors for treatment outcome, all five sessions in the trial with Facilitators present will be recorded. The SaS will be identical for those randomized to psilocybin or niacin active placebo.

Study Duration

The planned maximum study duration for each participant will be approximately one year, with variation primarily dependent on the length of the screening period, the number of days between baseline and dosing, and the visit windows provided for each post-dose assessment. For each participant, the study will be divided into two phases: Phase A or treatment phase (day 0 to and including day 42), and phase B or follow-up phase (day 43 – 365).

Primary Objective

The primary objective of this study is to evaluate the efficacy of a single 25 mg oral dose of psilocybin for major depressive disorder (MDD) compared to an active placebo (niacin) in otherwise medically-healthy participants between the ages of 20 and 65, assessed as the difference between groups in changes in depressive symptoms.

Primary Outcome Measure

Change in blinded rater MADRS total score from Baseline to Day 8.

Secondary Objective

One secondary objective is to evaluate the effect of psilocybin on synaptic density in prefrontal cortex and hippocampus, by comparing the change in [11C]UCB-J binding after psilocybin and placebo dosing.

The other exploratory objective is to study the clinical effect on depressive symptoms, clinical global impression, perceived disability and of post treatment use of antidepressants.

The following secondary endpoints will be evaluated to support the primary and secondary objectives:

Secondary Outcome Measures

- Relative increase (ratio) in [11C]UCB-J binding from Baseline to Day 15 post dose
- Change in MADRS total score from Baseline to Day 15, 42, 180 and 365 (end of study) respectively
- Change in MADRS total score from Baseline to the treatment period (mean of Day 8-365)
- Change in MADRS-S score from Baseline to Day 365 (end of study)
- Change in MADRS-S score from Baseline to the treatment period (mean of Day 8-365)
- Response rate at all time points post dose (MADRS and MADRS-S)
- Remission rate at all time points post dose (MADRS and MADRS-S)
- Change in Sheehan Disability Scale (SDS) score from Baseline to Day 365 (end of study)
- Change in SDS score from Baseline to the treatment period (mean of Day 8-365)
- Change in Clinical Global Impression (CGI) score from Baseline to Day 8, 42, 180 and 365 (end of study) respectively
- Change in CGI score from Baseline to the treatment period (mean of Day 8-365)
- Time to initiation of antidepressant treatment in follow up phase (day 43-365)

Exploratory Objectives

One exploratory objective is to evaluate the effect of psilocybin on biological markers of synaptogenesis, neuroinflammation, serotonergic activity and on self-rated depressive symptoms, reversal fear learning, clinical global functioning, anxiety symptoms, functional disability, health-related meaning of/satisfaction with life and psychological flexibility, subjective psychedelic experiences, participants' expectations prior to the/experience of the intervention, and facilitator and participants' in-session behaviors.

The purpose of collecting the exploratory outcome measures is to further understand the effect of psilocybin. The results will be reported in academic journals and not included in the Clinical Study Report.

Exploratory Outcome Measures (see table 1 for time points)

1. Biomarkers
   1.1. fMRI BOLD signal
   1.2. fMRI activation paradigms post dosing
   1.3. p11 (blood)
   1.4. BDNF and VEGF (plasma)
   1.5. monoamines and monoamine metabolites (CSF)

1.6. cytokines (CSF)
1.7. kynurenic acid and metabolites (CSF)

2. Scales
   2.1. General Anxiety Disorder 7 (GAD-7)
   2.2. Euroqol five dimension five level (E-5D-5L)
   2.3. Euroqol visual analogue scale (EQ-VAS)
   2.4. Mystical Experience Questionnaire (MEQ)
   2.5. Emotional Breakthrough Inventory (EBI)
   2.6. Challenging Experience Questionnaire (CEQ)
   2.7. Lasting Effects Questionnaire (LEQ)
   2.8. Treatment Expectation and Evaluation questionnaires
   2.9. Meaning of Life Questionnaire (MLQ)
   2.10. Satisfaction with Life Scale (SWLS)
   2.11. Acceptance in Action Questionnaire (AAQ-II)
   2.12. The Actively Living and Interconnecting Vitally in one's Embedded-world (ALIVE) Questionnaire
   2.13. Working Alliance Inventory-Short (WAI-S)
   2.14. Motivational Interviewing Treatment Integrity Code (MITI)
   2.15. Client Language Easy Rating (CLEAR)
   2.16. Scale for Psychedelic Intensity Rating (SPIR)

Safety Objectives

The overall safety objective of this study is to evaluate a single 25 mg oral dose of psilocybin compared to an active placebo in incidence, severity and frequency of Adverse Events (AEs), Treatment Emergent AEs (TEAEs), and Serious Adverse Events (SAEs) during and after the dosing session and at all follow-up visits. Solicited AEs will be compared from randomization until day 8 only.

Solicited Adverse Events

The following solicited AEs will be collected from randomization to Day 8:

Visual perceptual effects will be solicited by asking the following questions: 1) *Since your dosing session have you experienced any uncontrolled or disturbing return of study drug effects?*, and 2) *Since your dosing session have you experienced any visual distortions (e.g. geometric hallucinations, false perceptions of movement in the peripheral visual fields, flashes of color, intensified color, trails for images of moving objects, positive after-images, halos around objects)*? If a participant reports "Yes" to any of the above questions a study psychologist or psychiatrist will follow up for further assessment/diagnosis.

Additional solicited AEs include:

- Headache
- Nausea
- Elevated blood pressure (BP) as defined by systolic blood pressure (SBP) >140 mmHg or diastolic blood pressure (DBP) >90 mmHg on three separate readings and requiring medication (recorded for Periods 1-2; may result in early termination); and
- Elevated heart rate (HR) as defined as >100 beats per minute (BPM) lasting > 1 hour.

Unsolicited Adverse Events
Any other observed and participant reported AEs and SAEs will be recorded throughout the study, following written informed consent. SAEs will be reported in accordance with the medical Product Agency (MPA) guidelines.

Inclusion/ Exclusion Criteria

Inclusion Criteria
Individuals eligible to be randomized in this protocol are those who meet *all* of the following criteria:

1. Are 20 to 65 years old (inclusive) at the time of written informed consent at the In-Person Screening visit
2. Are able to read, speak, and understand Swedish
3. Are able and willing to adhere to study requirements, including attending all study visits, preparatory and follow-up sessions, and completing all study evaluations
4. Are able to swallow capsules
5. Women of childbearing potential (WOCBP) must agree to practice an effective means of birth control throughout the duration of the study, from Screening through the Day 43 assessment (see Section 15 Pregnancy for a definition of WOCBP)
6. Meet ICD-10 criteria for a diagnosis of remitting major depressive disorder and are currently experiencing a major depressive episode of at least a 30-day duration at the time of the Screening
7. Have sustained moderate-severe depression symptoms at Screening, as defined by a Screening MADRS total score ≥ 22 and ≤30% or ≤ 7 improvement (i.e. decrease) in MADRS total score from Web-Screening to In person screening (a standard 3 points will be added to the MADRS-S web screening to model the MADRS score).
9. Have an identified support person
    a. Agree to be driven/accompanied home (or to an otherwise safe destination) by the support person, or another responsible party, following dosing

Exclusion Criteria
Individuals not eligible to be randomized in this protocol are those who meet *any* of the following criteria:

1. Women who are pregnant, as indicated by a positive urine pregnancy test at Screening or Baseline. Women who intend to become pregnant during the study or who are currently nursing. Women who not agree to practice an approved birth control method for a period of 30 days following the dosing.
2. Current depressive episode lasting >5 years
2. Unwilling or unable to discontinue formal psychotherapy
3. Ongoing antidepressant drug treatment
4. Have previously during the current episode received the following non-medication treatments:
    a. deep brain stimulation (DBS)
    b. vagus nerve stimulation (VNS)
5. Currently receiving electroconvulsive therapy (ECT) or transcranial magnetic stimulation (TMS)

6. Unable or unwilling to discontinue any current medications that are known uridine diphosphate (UDP) or glucuronosyltransferase (UGT) enzyme modulators (eg valproate)
   - Note: Any prohibited agents must have been stopped at least 5x the elimination half-life of the specific drug at the time of Baseline. See Appendix A for a full list of prohibited medications.
8. Report psychedelic substances use ever. *Note:* Psychedelic substances include psilocybin, Lysergic acid diethylamide (LSD), mescaline (and natural products containing mescaline including peyote and San Pedro cactus), N,N-Dimethyltryptamine (DMT), natural products containing DMT including ayahuasca and 5-Methoxy-N,N-dimethyltryptamine (5-MeO-DMT), ibogaine, 2C compounds, 3,4-methylenedioxy-methamphetamine (MDMA), methylone or other psychedelics.
9. Have the following cardiovascular conditions:
   a. coronary artery disease, congenital long QT syndrome (prior diagnosis), cardiac hypertrophy, cardiac ischemia, congestive heart failure, myocardial infarction (prior diagnosis);
   b. tachycardia (defined as heart rate > 100 beats per minute);
   c. a clinically significant Screening ECG abnormality (e.g., atrial fibrillation);
   Note: A QTcF interval > 450 milliseconds is considered a clinically significant ECG abnormality
   d. artificial heart valve; or
   e. any other significant current or history of cardiovascular condition, based on the clinical judgment of study physician, that would make a participant unsuitable for the study
10. At Screening or Baseline have elevated blood pressure as defined as:
    a. Screening blood pressure SBP >135 mmHg or DBP > 85 mmHg on three separate readings; or
    b. Baseline blood pressure SBP >140 mmHg or DBP > 90 mmHg on three separate readings
11. Have a history of stroke or Transient Ischemic Attack (TIA)
12. Have moderate to severe hepatic impairment, as indexed by a Child-Pugh score ≥ 7
13. Have epilepsy
14. Have insulin-dependent diabetes
15. Are unable or unwilling to adhere to the following medication requirements:
    a. Agree to suspend sildenafil (Viagra®), tadalafil, or similar medications at least 72 hours prior to dosing
    b. If taking any supplement containing >20 mg of niacin, agrees to suspend use for the duration of the study
16. Have a positive urine drug test including Amphetamines, Barbiturates, Buprenorphine, Benzodiazepines, Cocaine, Cannabis, Methamphetamine, MDMA, Methadone, Opiates (Morphine, Oxycodone), Phencyclidine (PCP), and Tetrahydrocannabinol (THC). Exceptions are made for prescribed Benzodiazepines (stable dose for sleep or anxiety). *Note:* Benzodiazepine medications for sleep and non-benzodiazepine sleeping medications will be allowed to at discretion of the research physician. *Note:* Participants using cannabis, including legal cannabis, for any purposes will be excluded. Note: Participants who are taking prescription maintenance opiates, methadone or buprenorphine naloxone will be excluded.
17. Nicotine dependence that would disallow an individual to be nicotine free for the 7-10 hours during the dosing period

18. Meet ICD-10 criteria for schizophrenia spectrum or other psychotic disorders, including MDD with psychotic features (except substance/medication-induced or due to another medical condition), or Bipolar I, Bipolar II Disorder and Bipolar Disorder Not Otherwise Specified. *Note:* Participants with any lifetime diagnosis of schizophrenia spectrum or other psychotic disorders will be excluded
19. Meet ICD-10 criteria for antisocial personality disorder
20. Meet ICD-10 criteria for a moderate or severe alcohol or drug use disorder (excluding caffeine). *Note:* Participants with a diagnosis of alcohol or drug use disorder within the past 12 months will be excluded
21. Have presence of any psychiatric condition or symptom judged by the PI (or designee) to be a more significant clinical problem than MDD for the participant.
22. Have a first-degree relative with schizophrenia spectrum or other psychotic disorders (except substance/medication-induced or due to another medical condition), or Bipolar I Disorder
23. Have a psychiatric condition judged to be incompatible with establishment of rapport with the Facilitators or safe exposure to psilocybin
24. Report the following suicidal ideation or suicidal thoughts defined as:
    a. Have a score of ≥ 5 on Item 10 (suicidal thoughts) of the central-rater or computer administered MADRS at Screening or Baseline; or
    b. Have any suicidal ideation or thoughts, in the opinion of the study physician or PI, that presents a serious risk of suicidal or self-injurious behavior at any time prior to randomization
25. Have any suicidal ideation or thoughts, in the opinion of the study physician or PI, that presents a serious risk of suicidal or self-injurious behavior
26. Have any physical or psychological symptom, medication or other relevant finding at Screening or Baseline, based on the clinical judgment of clinical/medical study personnel, that would make a participant unsuitable for the study.
27. Have an allergy or intolerance to any of the materials contained in either drug product
28. Have one or more pathological blood test results as defined in 5.6.3 (as determined by a study physician).

Statistical Analysis

The difference between psilocybin and placebo in change from baseline to Day 8 in MADRS total score (primary endpoint) will be analyzed for the Full Analysis Set using Analysis of Covariance including treatment as main effect and baseline as covariate.

Based on prior controlled studies of psilocybin in cancer- related depression and anxiety[1], a decrease in depression symptoms from baseline to one-week post-dose of at least 30% is expected in the placebo arm.
In an open-label study of psilocybin[2] the change in MADRS total score from baseline to 1 week was -23.3. Several clinical studies in the literature report a standard deviation in change in MADRS total score around 10.

A sample size of 15 in each group will have 80% power to detect a difference in means of 11 (the difference between a psilocybin mean, μ_1, of 23 and a placebo mean, μ_2, of 12) assuming that the common standard deviation is 10 using a two group t-test with a 5% two-sided significance level.

The sample size is set to a total of 30 patients. Considering the short duration between treatment and primary endpoint measurement the risk of withdrawal is considered low. In case of withdrawal before day 15 (collection of USB-J binding) additional patients (maximum 6) will be randomized in order to achieve 30 patients completing 15 days.

4. INTRODUCTION

4.1 Acknowledgement

This protocol is in part based on information in the protocol "PSIL201 PROTOCOL IND # 129532, A Randomized, Double-Blind, Support-of-Concept Phase 2 Study of Single-Dose Psilocybin for Major Depressive Disorder (MDD)" from Usona Institute, 2800 Woods Hollow Rd, Madison, WI 53711; sponsor contact: Rob Barrow.

4.2 Purpose

The overarching purpose of this study is to conduct an initial examination of the safety, efficacy, tolerability and MDD relevant biological effects of single-dose psilocybin (3-[2-(dimethylamino)ethyl]-1H-indol-4-yl] dihydrogen phosphate) in otherwise medically healthy patients with recurrent MDD. The overall objectives of this study are to examine the efficacy of a single, fixed, 25 mg oral dose of psilocybin in reducing depressive symptoms and improving functional disability and quality of life in medically healthy patients with recurrent MDD when compared to active placebo; to compare the effect of biological markers of MDD in blood, CSF and using PET and MRI; to collect safety and tolerability data on single-dose psilocybin intervention in medically healthy patients with MDD, and to evaluate Facilitators' and participants' in-session behaviors on treatment outcome.

4.3 Study rationale

MDD is currently the leading cause of disability in the world (http://www.who.int/mediacentre/factsheets/fs369/en/). This highlights the urgent need to identify and test novel pharmacological agents that might benefit depressed patients who have not achieved symptom remission with currently approved antidepressant modalities. Recent studies suggest that psilocybin produces a substantial and sustained improvement in depressive symptoms following a single administration. It also highlights the need to identify the biological underpinning related to response to these novel treatments, in order to a) identify response markers and b) pave the way for rational drug development of novel antidepressant drugs.

4.4 Background

4.4.1 Major depressive disorder (MDD)

Major depression has become a health crisis of epidemic proportions in the modern world (http://www.who.int/mediacentre/factsheets/fs369/en/). The prevalence of major depression has risen over the last several generations in every country examined[3] and age of symptom onset has decreased[4]. It is estimated that major depression will rank second after cardiac disease as a cause of international medical morbidity by the year 2020[5]. Indeed, depression is now recognized to be a highly chronic and recurrent illness[6,7]. On average, patients with major depression are symptomatic 60% of the time, even when receiving community-

standard antidepressant treatments[8]. Depression is associated with greater disability than are most other chronic illnesses and is a risk factor for mortality[9]. In addition of increased risk of death by suicide, many studies indicate that depression significantly increases all-cause mortality independently of suicide. Depression predicts the later development of a number of medical conditions, including cardiac and cerebrovascular disease[10], hypertension[11], diabetes[12], obesity and metabolic syndrome[13] and cancer[14].
Unfortunately, most patients with depression do not experience a complete resolution of symptoms with antidepressant treatment[7]. The risks of not responding completely to (or tolerating) treatment have been highlighted by recent studies documenting that partial - but incomplete - response is associated with an increased risk of full symptomatic relapse (even when on therapy) and a worse long term disease course, as well as with significantly impaired quality of life[15–17]. Combined with the high prevalence and significant disability associated with MDD, the fact that currently available treatments are not fully adequate highlights the need to identify novel treatment strategies.

4.4.1.1 Pathophysiology of MDD

Converging evidence indicates that changes in selected neuropeptides, cytokines, neurotrophic factors, monoaminergic and glutamatergic signal transduction and, ultimately, brain plasticity play important roles in depression. Svenningsson et al have shown a relation between serotonin (5-HT) 1B receptors, the intracellular protein p11 and MDD[18] and that peripheral p11 levels may predict SSRI treatment response in patients[19]. Several groups have also published data for other promising peripheral biomarkers of MDD treatment effect related to serotonin, eg. the neurotrophic factors brain derived neurotrophic factor (BDNF) and vascular endothelial growth factor (VEGF)[20–22,23,24] , several different cytokines and kynurenic[25].

Chemical synapses are the predominant neuron-to-neuron contact in the central nervous system. Presynaptic boutons of neurons contain hundreds of vesicles filled with neurotransmitters. Changes in the number of synapses are associated with numerous brain disorders. Both basic research and clinical studies have demonstrated that depression can cause atrophy of prefrontal cortex (PFC) pyramidal neurons, decreased number of synapses and decreased expression of synapse related genes in rodent models and in postmortem tissue of depressed subjects[26–28]. Notably, imaging studies have shown a negative association between the number of depressive episodes and PFC thickness[29]. It is not clearly described which molecular mechanisms underpin a normalization of these structural changes in the recovery from MDD in man, but a study in rodents described in some detail a potential mechanism[30]: a low dose of ketamine was followed by transient activation of the mammalian target of rapamycin (mTOR). This effect could be blocked by the AMPA antagonist NBQX, as could the behavioral effects on depression by rapamycin (as quantified in the forced swim test, learned helplessness and novelty suppressed feeding test). Importantly, ketamine also increased synaptic proteins, spine number and spine density in proximal and distal segments in layer V pyramidal neurons of the mPFC within 6-72 hours. Synaptic strengthening was supported by analysis of excitatory post-synaptic current (EPSC) frequency responses to 5-HT.

The synaptic vesicle glycoprotein 2A (SV2A) is a transmembrane protein expressed ubiquitously in secretory vesicles in all brain areas[31]. SV2A is required for correct transmitter release, and is critical for synaptic function[32]. In addition to its role as the target for the drug levetiracetam[33], an established antiepileptic, dysfunction of SV2A has been implicated in eg. Alzheimer's disease[34]. While levetiracetam has shown antidepressant effects in rat[35], it is not

known whether it also has antidepressant properties in human. SV2A has been suggested as a marker for synaptic density, and recently the PET radioligand [^{11}C]UCB-J evaluated in non-human primate PET and in human controls and patients for synaptic density quantification[36,37]. [^{11}C]UCB-J PET represents the first method for in vivo quantification of human synaptic densities. First-in-human PET studies demonstrated that [^{11}C]UCB-J had excellent imaging properties. It was recently reported that MDD patients have lower [^{11}C]UCB-J binding in several brain regions implicated in the pathophysiology of depression, and that there is a negative correlation between MDD symptom severity and [^{11}C]UCB-J binding[38]. Although this is indicative of [^{11}C]UCB-J binding being a marker of MDD state, this has not yet been shown.

4.4.2 Psilocybin

Psilocybin is a natural product produced by numerous species of *Psilocybe* mushrooms. The phosphate group is enzymatically cleaved in the body to produce psilocin, an agonist at a variety of serotonin receptors, the most important of which for its behavioral effects is the 5-HT2A receptor[39,40]. Psilocybin was first isolated from *Psilocybe* mushrooms in 1957, followed by *de novo* synthesis in 1958[41]. Psilocybin was marketed worldwide by Sandoz in the 1960s as *Indocybin*™ for experimental and psychotherapeutic purposes. Although it was well tolerated and demonstrated potentially useful effects, it was classified as a controlled substance, placed in Schedule I in 1970, and effectively removed from clinical use or scientific study. Psilocybin, and similar drugs such as lysergic acid diethylamide (LSD) and mescaline, fall into a pharmacological class referred to in this application as "classic psychedelics" to differentiate them from other psychoactive substances (i.e. MDMA [3,4-methylenedioxy-methamphetamine] that have different psychological/ behavioral effects and different adverse effect profiles and risk/benefit ratios than psilocybin[42,43].

The psilocybin used in this study is synthetically manufactured in a laboratory and meets quality specifications suitable for human research use. No mushrooms naturally containing psilocybin are used in the manufacturing process. The active drug is encapsulated using a hydroxypropyl methylcellulose (HPMC) capsule and contains 25 mg of psilocybin. The active placebo is encapsulated using a HPMC capsule and contains 100 mg of niacin USP. Psilocybin and niacin are administered orally and taken with water.

Information regarding the pharmacology and toxicology of psilocybin can be found in the Investigator's Brochure (IB).

4.4.2.1 Biological effects related to psilocybin as an antidepressant

In vivo in man, it was recently confirmed using PET and the radioligand [^{11}C]CIMBI-36 that psilocin binds to the 5-HT2A receptor in a dose dependent way, and that there is a correlation between psilocin occupancy of the 5-HT2A receptor and the acute hallucinogenic symptomatology[44]. Interestingly, studies in preclinical models have shown that 5-HT2A agonists such as psilocybin induce synaptogenesis and neuroplasticity acutely in the pathway shared by the rapid antidepressant ketamine described above: TrkB and mTOR and that this effect can be blocked by the 5-HT2A antagonist ketanserine[45].

4.4.3 Previous Clinical Experience with Psilocybin Relevant to MDD

Data reaching back to the 1960s propose that classic psychedelics, including psilocybin, have behavioral effects relevant to the treatment of depression. Recent studies indicate that psilocybin may possess antidepressant properties. Specifically, two randomized, double-blind, placebo-controlled studies in patients with life-threatening cancer and clinically-

significant depression/anxiety and an open trial in patients with Treatment-Resistant Depression (TRD) advocate that a single intervention with psilocybin conducted within a set and setting (SaS) protocol, described below, produces antidepressant effects that are sustained for up to six months post- intervention, while having a minimal, time-limited (hours) side effect profile. However, the available data that directly support this possibility are currently limited to a 12 participant open trial in TRD and to the fact that a subset of participants in the cancer depression/anxiety studies met criteria for MDD and had failed previous treatments with antidepressants[1,2,46].

Placebo-controlled studies of psilocybin to treat depression have only been conducted in patients with cancer, not in medically-healthy patients. And the single 12 participant study conducted to date of psilocybin intervention in medically-healthy patients with TRD was not blinded and did not include a placebo condition. The scientific rationale for the current study is thus to address both these methodological gaps in understanding the potential value of single dose psilocybin for MDD in medically-healthy patients, and, importantly, explore the biological underpinning of this effect. Specifically, this study will address this by: 1) randomizing a population of medically-healthy participants with MDD; 2) utilizing a randomized, double-blind, placebo-controlled, parallel-group design to control for baseline covariates, placebo and other therapeutic effects not specifically linked to medication exposure; and 3) utilizing PET and the radioligand [^{11}C]UCB-J to test the hypothesis that successful psilocybin treatment of MDD is associated with increased synaptic density in MDD relevant brain regions, analysis of peripheral blood mononuclear cells (PBMCs), plasma and CSF with regards to potential biomarkers of MDD and psilocybin response can be used, and a MRI paradigm to explore associations between treatment expectation and CNS metabolic consequences of treatment.

In addition and of importance, data derived from this study will allow for a refinement of effect size estimates for larger phase 3 studies of single dose psilocybin in MDD. Moreover, because previous psilocybin studies utilized weight-based dosing, the current study will allow us to examine whether fixed-dose psilocybin will produce effect size benefits consistent with those seen with weight-based dosing.

4.5 The Set and Setting (SaS) Protocol

Early studies with psilocybin, administered for research purposes done without a supportive setting, observed a wide range of responses, including panic reactions and episodes of paranoia that were highly distressing[47,48]. From these early experiments came an increasing appreciation of the importance of designing the optimal conditions under which psilocybin could be safely administered within the research setting. Such attention was aimed at reducing the risk of these negative responses while simultaneously increasing the likelihood of participants having the types of positive mystical-type experiences that has been hypothesized to play a role in long-term positive behavioral/emotional changes. By addressing what are now known as “set” (i.e. participant emotional/cognitive/behavioral state/mindset and expectations just prior to psilocybin exposure) and “setting” (the physical environment in which the exposure occurs), the rate of adverse responses to classic psychedelic exposure dropped significantly. Confirmation of the importance of context for optimizing the therapeutic benefit of psilocybin (and other psychedelics) is aggregated in a meta-analysis of 23 controlled studies and provides a strong rationale for the use of the SaS in this protocol[49].

By the mid-1960s, a set and setting approach had been widely accepted as including three components: 1) preparation prior to drug session; 2) drug session; and 3) post session meetings to integrate the psychedelic experience. In component 1, participants underwent pre-exposure preparation sessions designed to build rapport with the Facilitators who would be present during the drug exposure session and to identify personal themes and struggles that might be especially likely to impact the session experience. In component 2, the drug session itself was conducted by two Facilitators who were present throughout the session. Sessions were typically conducted in a room designed to be quiet, comfortable, and aesthetically pleasing, and participants were encouraged to wear eyeshades and listen to a program of music on headphones during the drug exposure to aid them in focusing their attention inward. In component 3, participants engaged in a series of drug-free interview meetings of variable frequency, sometimes over a period of several weeks, to discuss their session experience thoroughly.

The SaS protocol proposed in the current study is in line with this 3-component model. A full and detailed description of information and instructions given during the study preparation, dosing, and integration sessions is provided in the PSIPET Manual for Clinical Facilitators. The intervention protocol, for purposes of this research study, includes the period of participant preparation prior to drug administration, the 7-10 hour drug session, and the post-dose integration sessions to maximize participant safety and support. It is for this reason that eligibility verification is conducted prior to the preparatory sessions rather than immediately preceding the delivery of study medication (psilocybin vs. active placebo).

5. OBJECTIVES AND ENDPOINTS

5.1 Primary objective

The primary objective of this study is to evaluate the efficacy of a single 25 mg oral dose of psilocybin for recurrent major depressive disorder (MDD) compared to an active placebo (niacin) in otherwise medically-healthy participants ages 20 to 65, assessed as the difference between groups in changes in depressive symptoms.

5.1.1 Primary study endpoint

Between-group difference in change of blinded rater Montgomery-Asberg Depression Rating Scale (MADRS) total score from Baseline to post-dose Day 8.

5.1.1.1 Justification for primary study endpoint

As with other rapidly acting investigational antidepressant agents (e.g. ketamine), a single dose of psilocybin has shown to induce a large effect-size reduction in depressive symptoms within a day of administration in comparison to active placebo, with no further improvement in symptom scores seen at subsequent assessment points (i.e. at 2 weeks and 6 weeks post-intervention)[1]. These findings demonstrate that an assessment at one week post-intervention will likely capture the entire antidepressant effect of psilocybin, both in comparison to active placebo and in comparison to baseline symptom score. Further support for the appropriateness of one week post-intervention as the primary study endpoint comes from the association of immediate (e.g. one day post-intervention) and longer-term (e.g. six weeks post-intervention) antidepressant responses to both psilocybin and niacin in the New York University (NYU) study of depressed/anxious patients with cancer. In that study reductions in Beck Depression Inventory (BDI) and Hospital Anxiety and Depression Scale (HADS)

depression subscale scores between baseline and one day post-intervention were similar to reductions in BDI and HADS depressive subscale scores between baseline and six weeks post-intervention (*unpublished data*), as shown in this table:

Instrument	Niacin	Psilocybin
HADS-Depression	**.750** (*p* = *.002*)	**.925** (*p* = *.000016*)
BDI	**.863** (*p* =*.001*)	**.877** (*p* = *.000082*)

These results indicate that the impact of psilocybin on depressive symptoms at one-week post- intervention is likely to be a near perfect proxy for its longer-term effects, which further strengthens its appropriateness as a primary study endpoint in the proposed study. Because this study proposes the impact of psilocybin vs. active placebo on MADRS score at Day 42 post-dose as a secondary endpoint, results from the NYU study strongly suggest that this endpoint will be met, if indeed the primary endpoint is confirmed.

The selection of the MADRS to assess the study's primary endpoint is consistent with it being the most commonly used assessment tool for assessment of primary endpoints in recent phase 3 antidepressant registration trials. This is consistent with data indicating that in comparison to the other historically-used instrument, the Hamilton Depression Rating Scale (HAM-D), the MADRS shows improved ability to capture meaningful clinical improvement in response to active antidepressants versus placebo[50,51].

5.1.2 Secondary endpoints

The following secondary endpoints will be evaluated to support the primary objective:

- Change in MADRS total score from Baseline to post-dose Day 15, 42, 180 and 365 (end of study) respectively.
- Change in MADRS total score from Baseline to the treatment period (mean of Day 8-365)
- Change in MADRS-S score from Baseline to Day 365 (end of study)
- Change in MADRS-S score from Baseline to the treatment period (mean of Day 8-365)
- Response rate at all time points post dose (MADRS and MADRS-S)
- Remission rate at all time points post dose (MADRS and MADRS-S)
- Change in Sheehan Disability Scale (SDS) score from Baseline to Day 365 (end of study)
- Change in SDS score from Baseline to the treatment period (mean of Day 8-365)
- Change in Clinical Global Impression (CGI) score from Baseline to Day 8, 42, 180 and 365 (end of study) respectively
- Change in CGI score from Baseline to the treatment period (mean of Day 8-365)
- Time to initiation of antidepressant treatment

5.2 Secondary objective and endpoint

The secondary objective of this study is to evaluate the efficacy of a single 25 mg dose of psilocybin for MDD compared to an active placebo in medically-healthy participants ages 20 to 65, to increase synaptic density in hippocampus and prefrontal cortex. measured as between-group differences in change in [^{11}C]UCB-J binding ratio from Baseline to day 15 post dose.

5.2.1 Secondary endpoints

- Relative increase (ratio) in [11C]UCB-J binding from Baseline to Day 15 post dose

5.3 Exploratory Objectives and endpoints

The exploratory objectives of this study are to evaluate the efficacy of a single 25 mg dose of psilocybin for MDD compared to an active placebo in medically-healthy participants ages 20 to 65, measured as between-group differences in:

- Change in p11 concentration in PBMC subsets from Baseline to day 15 Post dose
- Change in BDNF and VEGF concentration in plasma from Baseline to day 15 Post dose
- Change in kynurenic acid in CSF from Baseline to day 15 Post dose
- Change in monoamine and monoamine metabolites in CSF from Baseline to day 15 Post dose
- Change in cytokine concentration (exact markers to be determined based on current literature at time of analysis) in CSF from Baseline to day 15 Post dose
- Change in metrics of whole-brain connectomics and hub characteristics of medial and ventrolateral prefrontal cortex as well as orbitofrontal cortex in the psilocybin treated group as compared to the placebo group, and in relation to clinical outcome (MADRS) as well as PET-outcome (resting state fMRI).
- Change in influence of top-down priors in an instructed fear learning paradigm and its relation to a change in activity of the ventromedial prefrontal cortex and lateral orbitofrontal cortex in the psilocybin treated group as compared to the placebo group, and in relation to clinical outcome (MADRS) as well as PET-outcome (tasked based fMRI).
- Change in Emotion detection under perceptual uncertainty and perspective-shifting ability in relation to large-scale network activity patterns in the psilocybin treated group as compared to the placebo group, and in relation to clinical outcome (MADRS) as well as PET-outcome.
- Change in anxiety symptoms from Baseline to post-dose Day 8, 15, 42 and 365
- Change in functional disability to post-dose Day 8, 15, 42 and 365
- Change in health-related meaning of life and satisfaction with life from Baseline to post-dose Day 8, 15, 42 and 365
- Change in psychological flexibility from Baseline to post-dose Day 8, 15, 42 and 365
- Correlations between biomarkers and scales

5.4 Safety Objectives

The overall safety objective of this study is to evaluate a single 25 mg oral dose of psilocybin compared to an active placebo in incidence, severity and frequency of Adverse Events (AEs), Treatment Emergent AEs (TEAEs), Solicited AEs, and Serious Adverse Events (SAEs) before, during and after the dosing session and at all follow-up visits.

5.4.1 Specific Safety Monitoring Objectives

Safety will be continually evaluated by study staff through monitoring and assessment of AEs, vital signs, concomitant medication use, physical exams, on-site rater administered measures, and integration with session Facilitators. The study will also assess abuse liability, including non-clinical, illicit use of psilocybin and other psychedelics and other illicit and non- prescribed drug use via the AUDIT and DUDIT, participant self-report, and urine drug testing per the Schedule of Assessments.

5.4.2 Safety Endpoints

Specific safety endpoints are as follows:

Differences between the psilocybin and active placebo groups in:
- Incidence of AEs by severity
- Incidence of AEs leading to termination from the study
- Incidence of TEAEs
- Incidence of TEAEs by severity
- Incidence of solicited AEs
- Incidence of solicited AEs by severity
- Incidence of SAEs

5.5 Outcome Measures

5.5.1 Montgomery-Asberg Depression Rating Scale (MADRS)

The MADRS is a 10-item depression rating scale developed in 1979 to be more sensitive to symptom changes induced by antidepressants than were currently available instruments[52]. The MADRS has been used for assessing primary outcomes in registration trials for new pharmacological agents for treating MDD, whether these agents are traditional monoamine modulators or agents with novel mechanisms of action (e.g., esketamine)[53]. The MADRS includes questions on the following symptoms 1) Reported sadness, 2) Apparent sadness, 3) Inner tension, 4) Reduced sleep, 5) Reduced appetite, 6) Concentration difficulties, 7) Lassitude, 8) Inability to feel, 9) Pessimistic thoughts, and 10) Suicidal thoughts. Items are scored via a clinical interview that progress from more broadly phrased questions about symptoms to more detailed queries that allow a precise rating of severity. Items are rated to capture the patient's clinical state over the prior week. Each item yields a score of 0 to 6, and higher scores indicate more severe depression. The overall score ranges from 0 to 60.

Various studies have suggested cut scores from < 9 to <12 as a definition of remission. For the current study, remission will be defined as a score ≤ 10. Response will be defined as ≥ 50% decrease compared to the last measurement before dosing.

MADRS-S is the self-rating version of MADRS. Apart from item 1 in MADRS, that is deleted from MADRS-S, the two scales are identical.

To reduce the risk of functional unblinding during collection of the primary study outcome measure, MADRS assessments will be conducted by a blinded rater. Raters will be blinded to participant allocation. At any occasion, if a face to face interview is not possible, a non-recorded video interview via an encrypted link will take place.

MADRS ratings will be performed at screening, PET1, day 0, 8, 15, 42, 180 and 365.

At day of dosing, results of MADRS (item 2-10) and MADRS-S before dosing will be used for validation purposes.

There is a lack of scientific evidence supporting that any instrument has sufficient accuracy to predict future suicide with 80% sensitivity and 50% specificity or better[54]. However, MADRS item 10 (MADRS-S item 9) address suicidal ideation. A score of ≥ 5 at any time point suggests that a participant may be experiencing a level of active suicidal ideation and will necessitate clinical assessment, management and disposition.

The result on each MADRS item will be documented by the interviewer in the eCRF at time of interview.

5.5.1.1 MADRS self-rating (MADRS-S)

In order to get preliminary information on time to response and remission, duration of clinical effect, the self-rating version of MADRS[55] will be administered at web-screening, PET1, day 0 prior to dosing, and day 1-8, 15 and monthly from day 42, 180 and 365 electronically via the participants smart phone or computer at the site as implemented the Region Stockholm web based patient interface 1177.se.

5.5.2 Sheehan Disability Scale (SDS)

The SDS will be utilized to determine the impact of psilocybin vs. placebo on functional disability (psychosocial functioning). This will be a key secondary endpoint for the current study. The SDS is a composite of three self-rated items designed to measure the extent to which three major sectors in the patient's life are impaired by psychiatric symptoms, including depression[56]. This scale has been used widely in psychopharmacology randomized controlled trials and has been accepted by the Food and Drug Administration (FDA) for functional disability labeling. The SDS uses visual-spatial, numeric, and verbal descriptive anchors simultaneously to assess disability across three domains: work, social life, and family life. The SDS was developed as an intervention outcome measure that would be sensitive to change and to drug placebo differences over time. The SDS asks patients to rate the extent to which his or her 1) work/school, 2) social life or leisure activities, and 3) home life or family responsibilities are impaired by his or her symptoms on a 10-point visual analog scale. There are verbal descriptors for the points on the scale as well as numerical scores that provide more precise levels of the verbal descriptors. Typically, four scores are derived from the scale in research studies – one for each of the work, social life and family life disability measures and an aggregate total score of these three scores combined.

SDS is a self-report instrument that will be collected electronically at day -3, 0, 8, 15, 42 and then monthly until 365, via the participants smart phone or computer as implemented the Region Stockholm web-based patient interface 1177.se.

5.5.3 General Anxiety Disorder 7 (GAD-7)

GAD-7 was originally designed as a brief screening tool and symptom severity measure for GAD[57], but is increasingly being used as a broad general measure of anxiety, sufficiently sensitive to detect panic disorder, social phobia, and posttraumatic stress disorder[58]. The self-

report questionnaire, designed to assess the patient's status during the previous 2 weeks, is widely used in both research and clinical settings. It contains seven items with a score range from 0 to 21, and has both high levels of internal consistency (a 1⁄4 .89 – .92) and test – retest reliability (r 1⁄4 .83)[59,60].

GAD-7 is a self-report instrument that will be collected electronically at day -3, 0, 8, 15, 42 and then monthly until 365, via the participants smart phone or computer at the site as implemented the Region Stockholm web-based patient interface 1177.se.

5.5.4 Clinical Global Impression – Improvement and Severity (CGI-I and CGI-S)

The CGI rating scales are measures of symptom severity, intervention response and the efficacy of interventions in intervention studies of patients with mental disorders[61]. The CGI-S is a 7-point scale that requires the rater to rate the severity of the patient's illness at the time of assessment, relative to the clinician's past experience with patients who have the same diagnosis. Possible ratings are:

1. Normal, not at all ill
2. Borderline mentally ill
3. Mildly ill
4. Moderately ill
5. Markedly ill
6. Severely ill
7. Among the most extremely ill patients

CGI-I is a 7-point scale that requires the rater to rate the change of the patient's illness compared to the baseline (ie first) assessment regardless of cause. Possible ratings are:

0. Not assessed
1. Very much improved
2. Much improved
3. Minimally improved
4. No change
5. Minimally worsened
6. Much worsened
7. Very much worsened

CGI- S and CGI-I will be collected at the same time points (day 0 (only CGI-S), 8, 15, 42, 365), by the same rater, as MADRS, and the results will be documented by the interviewer in the eCRF at time of interview.

5.5.5 EQ-5D

EQ-5D[62] is a valid, reliable and responsive self-rating of quality of life. Five dimensions of quality of life, mobility, self-care, usual activities, pain/discomfort and anxiety/depression are quantified in three (EQ-5D-3L) or five (EQ-5D-5L) levels of severity. There is also a visual analogue scale (VAS) for quantification of overall health status.

EQ-5D is a self-report instrument that will be collected electronically at day -3, 0, 8, 15, 42 and then monthly until 365, via the participants smart phone or computer as implemented the Region Stockholm web-based patient interface 1177.se.

5.5.6 Challenging Experience Questionnaire (CEQ)

CEQ is a validated 26-item self-report scale developed in 2016[63] to measure acute adverse psychological reactions to psilocybin and other classic hallucinogens. At the end of the dosing session, participants rate the degree to which at any time during that session they experienced potential adverse symptom on a scale from 0 (none; not at all) to 5 (extreme; more than ever before in my life).

CEQ will be administered by the Facilitators on paper and then transferred to the eCRF by study personnel, upon completion of the dosing session when all behavioral effects of the intervention have resolved.

5.5.7 Mystical Experience Questionnaire (MEQ-30)

The MEQ is a self-report measure developed to assess the immediate effects of classic psychedelics. It is based on Stace's conceptual framework of mystical experiences[64], and covers the major dimensions of classic mystical experience: unity, transcendence, noetic quality, sacredness, positive mood, and ineffability/paradoxicality. The MEQ has been administered in various forms in a number of studies over the past 50 or more years[47,48,65–70]. Items on the questionnaire are rated on a 6-point scale, from 0 (none; not at all) to 5 (extreme; more than ever before in my life). A factor analysis conducted by MacLean et, al. found that the most recent version (MEQ30), is a psychometrically sound instrument for assessing psychedelic-occasioned mystical experiences[63].

MEQ-30 will be administered by the Facilitators on paper and then transferred to the eCRF by study personnel, upon completion of the dosing session when all behavioral effects of the intervention have resolved.

5.5.8 Emotional Breakthrough Inventory (EBI)

The EBI is a new 6-item scale developed by researchers at Imperial College in London, that assesses the presence and severity of emotionally challenging/distressing experiences that can occur during a psychedelic experience. The scale utilizes visual analog responses captured on a line from "not at all" to "very much so". Experiences queried include 1) facing emotionally difficult feelings that are usually pushed aside; 2) experiencing a resolution of a personal conflict/trauma; 3) being able to explore challenging emotions and memories; 4) having an emotional breakthrough; 5) getting a sense of closure on an emotional problem, and 6) achieving an emotional release followed by a sense of relief.

EBI will be administered by the Facilitators on paper and then transferred to the eCRF by study personnel, upon completion of the dosing session when all behavioral effects of the intervention have resolved.

5.5.9 Treatment Expectation

Treatment Expectation is a compilation of questions regarding the participants' expectation of the forthcoming treatment in five areas: Their treatment expectation; whether they will feel safe and seen during sessions; if they think the treatment will help them; and if the treatment will make them satisfied. The questions are answered on a scale ranging from 0 (not at all) to 4 (to a very high degree). At the end of the form, participants will also answer a question regarding how much they think the treatment will reduce their depression on a scale ranging from 0 to 100.

The questionnaire will be administered on paper and then transferred to the eCRF by a study coordinator, before the first treatment session.

5.5.10 Treatment Evaluation

Treatment Evaluation is a compilation of questions regarding the participants' treatment experience in five areas: Their experience during sessions; if they felt safe and seen; if they think the treatment was helpful; and if the treatment made them satisfied. The questions are answered by the participants on a scale ranging from 0 (not at all) to 4 (to a very high degree). At the end of the form, participants will also answer a specific question regarding how much they think the treatment will reduce their depression, on a scale ranging from 0 to 100, and a question regarding any recent lifestyle changes.

The questionnaire will be administered on paper and then transferred to the eCRF by a study coordinator, after the last treatment session.

5.5.11 Acceptance in Action Questionnaire (AAQ-II)

AAQ-II is a brief 7-item measure of psychological flexibility with a satisfactory structure, reliability and construct validity[71]. Participants score value statements on a scale ranging from 1 (never true) to 7 (always true) to capture six core processes of psychological flexibility: Present moment awareness; Acceptance of experiences; Defusion from the literal belief in thoughts; Values clarification; The identification of specific behaviors in the service of those values (committed action); and, Contact with a flexible experience of the self (self-as-context)[72]. Lower scores on the AAQ-II indicate greater levels of psychological flexibility and predict a range of outcomes including wellbeing in patients with anxiety and depression[73,74]. The theory of psychological flexibility has proposed to fit with the phenomenological experience of psychedelics, and thus act as a good research guide for how these treatments work in the acute phase, and may lead to lasting change[75–77].

AAQ-II will be administered together with ALIVE by the Facilitators on paper and then transferred to the eCRF by study personnel, in conjunction with the first (Preparation session), fourth (Integration session number two), and fifth (Integration session number three) treatment session. At Day 42 and 365, AAQ-II will be administered on paper and then transferred to the eCRF by a study coordinator.

5.5.12 Actively Living and Interconnecting Vitally in one's Embedded-world (ALIVE)

Alive (Bond and Dowling, *in press*) is another measure of psychological flexibility that measures three indivisible, or interwoven, activities: Learning to actively tune-in to perceptions; Discover multiple possibilities; Take a stand as the person (the participant) wish to become. ALIVE is a brief 6-item self-report scale with value statements on a scale ranging from 1 (never true) to 7 (always true). Psychometric analyses with over 13.000 participants across eight studies appear to support the construct validity of the ALIVE measure. Analyses include exploratory and confirmatory factor analyses, (supportive) CFA invariance analyses, structural equation models that examine concurrent validity, autoregressive longitudinal panel studies, and quasi-experiments (Bond and Dowling, *in press*).

ALIVE will be administered together with AAQ-II by the Facilitators on paper, and then transferred to the eCRF by study personnel, in conjunction with the first (Preparation session), fourth (Integration session number two), and fifth (Integration session number three) treatment session. At Day 42 and 365, ALIVE will be administered on paper and then transferred to the eCRF by a study coordinator.

5.5.13 Meaning of Life Questionnaire (MLQ)

MLQ[78] is a brief 10-item self-report measure of the search for and presence of meaning in life, with value statements on a scale ranging from 1 (absolutely untrue) to 7 (absolutely true). MLQ has shown to be a reliable, structurally sound measure of the search for/presence of meaning, and presents a number of improvements over other measures of meaning of life, including more precise measurement, greater structural stability, and assessment of the search for meaning[78].

MLQ will be administered together with SWLS by the Facilitators on paper, and then transferred to the eCRF by study personnel, in conjunction with the first (Preparation session), fourth (Integration session number two), and fifth (Integration session number three) treatment session. At Day 42 and 365, MLQ will be administered on paper and then transferred to the eCRF by a study coordinator.

5.5.14 Satisfaction with Life Scale (SWLS)

SWLS[79] was developed as a measure of the judgmental component of subjective well-being. The high convergence of self- and peer-reported measures of subjective well-being and life satisfaction provide strong evidence that subjective well-being is a relatively global and stable phenomenon. SWLS is shown to be a valid and reliable measure of life satisfaction, suited for use with a wide range of age groups and applications. SWLS is a brief 5-item self-report measure with value statements on a scale ranging from 1 (strongly disagree) to 7 (strongly agree).

SWLS will be administered together with MLQ by the Facilitators on paper, and then transferred to the eCRF by study personnel, in conjunction with the first (Preparation session), fourth (Integration session number two), and fifth (Integration session number three) treatment session. At Day 42 and 365, SWLS will be administered on paper and then transferred to the eCRF by a study coordinator.

5.5.15 Working Alliance Inventory-Short (WAI-S)

The Working Alliance Inventory[80] and the Working Alliance Inventory-Short[81] are widely used measures of alliance in both psychotherapy and research settings. The WAI was originally derived from a well-researched framework on the working alliance developed by Bordin[82]. WAI-S contains 12 items rated on a seven points scale. It provides a summary of the total working alliance and three subscales to assess primary components of the working alliance: Goal (i.e., agreement with regard to the treatment goals), Task (i.e., agreement with regard to the tasks) and Bond (i.e., the empathic bond between the client and the therapist). In this study, the Facilitator and the participant each complete WAI-S at the end of the same treatment sessions. Both forms also contain two additional questions regarding empathy and partnership/collaboration, as defined in the MITI.

The participants' form will be administered on paper and then transferred to the eCRF by a study coordinator, after session one (Preparation session) and four (Integration session number two). The Facilitators' form will be answered by the Facilitators on paper, and then transferred to the eCRF, after the same treatment sessions.

5.5.16 The Lasting Effects Questionnaire (LEQ)

LEQ is a fusion of 18 questions from CEQ, MEQ and EBI, aiming to measure the lasting effects of the participants' acute subjective psychedelics experience. Items on the

questionnaire are rated on a 100-point scale, from 0 (none; not more than usual) to 100 (Extremely much/maximum).

LEQ will be administered by the Facilitators on paper, and then transferred to the eCRF by study personnel, in conjunction with the fourth (Integration session number two), and fifth (Integration session number three) treatment session. At Day 42 and 365, LEQ will be administered on paper and then transferred to the eCRF by a study coordinator.

5.5.17 Manual for conducting semi-structured interviews

The manual for the semi-structured interviews consists of three themes with questions for the Facilitators and participants respectively: 1) Therapeutic activity (e.g., *What was your most important function during treatment sessions? During which moment where you most/least active*); 2) Experience of structure and content (e.g., *Which parts of the sessions were most/least helpful for the participants, and in what way?*; 3) Outcome (e.g., *What effect did the treatment have on you?).*

All interviews will be conducted by telephone after Day 42, with a duration of approximately thirty to forty minutes. They will be optional for both Facilitators and participants, audiotaped and then transcribed.

5.5.18 Motivational Interviewing Treatment Integrity Code (MITI)

The Motivational Interviewing Treatment Integrity (MITI) Code is a coding system with acceptable psychometric properties[83] widely used as a treatment Motivational interviewing (MI) integrity measure and as a feedback tool in MI training and supervision. MITI classifies and quantifies therapist's (i.e., the Facilitators') in session verbal behavior, and consists of two main components: (1) The global dimensions; and (2) The behavior counts, which are frequency counts of the practitioners' every utterance coded in specified categories.

All study coding will be performed by the trained coders at Motivational Interviewing Quality Assurance (MIQA), at Karolinska Institutet https://www.miqagruppen.org.

5.5.19 Scale for Psychedelic Intensity Rating (SPIR)

SPIR is a Likert scale previously applied to measure the psychedelic intensity rating after psilocybin dosing[44]. All subjects are asked to rate psychedelic intensity from 0 = not intense at all, to 10 = very intense. Facilitators will ask the patient to rate psychedelic intensity 90 minutes after dosing.

The participants' verbal response will be coded in the eCRF by a study coordinator at time of rating.

5.5.20 The Client Language Easy Rating (CLEAR)

CLEAR is a coding scheme designed to classify and quantify in-session client (i.e., participant) language[84]. Although the client language is categorized into unique codes, only Change Talk (utterances in the direction of behavior change) and Sustain Talk (utterances in the direction of maintaining current behaviors) are coded, all neutral client language and all Facilitator language are ignored during coding with CLEAR. As such, CLEAR focuses upon the types of in-session client language that have been predictive of future change (or non-change); Increased sustain talk has a negative effect on healthier client behavioral outcomes, and the proportion of change talk has a small positive effect on positive behavioral outcomes

[85,86]. The benefits of CLEAR are its simplicity, relative ease of training and use, non-reliance on session transcripts, and ability to calculate the Percentage Change Talk variable[84].

All study coding will be performed by the trained coders at Motivational Interviewing Quality Assurance (MIQA), at Karolinska Institutet https://www.miqagruppen.org.

5.6 Eligibility and Safety Measures

5.6.1 The MINI Structured Interview for ICD-10 diagnosis

A MINI interview[87] will be performed by a trained physician after informed consent has been given. The MINI includes semi structured interview support for diagnosis of MDD, anxiety disorders, bipolar disorder, psychotic disorders, alcohol and drug abuse disorders.

5.6.2 Questionnaire Order of Assessments

At all visits, starting at Baseline, the rater MADRS interview will be conducted prior to all other outcome assessments, followed by additional rater assessments, patient-reported outcome measures and on-site rater administered assessments. Specific order of outcome assessments is provided in the CRF.

5.6.3 Laboratory Assessments

The following laboratory assessments will be performed at screening: Complete Blood Count with Differential (CBC w/ Diff), PK-INR, APTT, Na, K, creatinine, ALAT, ASAT, ALP, Ca, albumin, CDT, PEth, Thyroid Stimulating Hormone (TSH), high-sensitivity C-Reactive Protein (hs-CRP), HBV, HCV, HIV. Urine Drug Test and Pregnancy Testing will be done at screening, PET1 and PET2.

6. PROTOCOL DESIGN

6.1 Overall Study Design

Thirty patients (males and females) ages 20 to 65 who, at Screening, meet ICD-10 criteria for recurrent MDD with a current depressive episode of at least a 30-day duration, an on-site Screening MADRS score ≥ 22 (consistent with moderate or greater depressive symptom severity), ≤ 30% symptom decrease from web-screen, and who meet all other inclusion/exclusion criteria at Baseline will be enrolled into the study and randomized with a 1-to-1 allocation under double-blind conditions to receive a single 25 mg oral dose of psilocybin or a single 100 mg oral dose of niacin. Expecting a maximum of 20% drop out rate a maximum of 6 more subjects may be randomized, in order to reach 30 patients completed (performed all study related activities up until and including PET#2 day 15). Niacin will serve as an active placebo that provides an acute physiological response (flushing) that may aid in blinding of intervention allocation. All randomized participants will be included in the Full Analysis Set (FAS) that will be used for analyzing primary and secondary study endpoints.

Eligible participants at Screening will undergo pre dosing data acquisition, preparation session and be eligible for randomization on Dosing Day to receive either psilocybin or niacin active-placebo and will complete follow-up visits and assessments on study Day 1, 8, 15 and 42 (within corresponding visit windows). Study outcome measures will assess depressive symptoms, clinical global functioning, anxiety symptoms and health-related

quality of life. Safety outcome measures will be collected at all assessment time points from the time of consent through the end of study.

To enhance participant safety, the current study does not propose to test psilocybin as a “context- less” pharmacological agent, but rather within a “set and setting” (SaS) protocol similar to the protocol that has been used in all modern studies of psilocybin in both diseased and normal healthy populations. The SaS protocol for this study includes: 1) a preparation visit with session Facilitators prior to dosing; 2) administration of study medications in an aesthetically neutral room under the supervision of two Facilitators who are present throughout the session (with the exception of short, temporary allowances for facilitator breaks; e.g. bathroom breaks); and 3) three post-dose integration sessions during which participants are encouraged to discuss their intervention experience with the Facilitators. To evaluate the Facilitators' adherence to the study manual, and the role of Facilitators' and participants' in-session behaviors for treatment outcome, the preparation session and all three integration sessions will be recorded using a digital voice recorder. The dosing session will be filmed using a digital film camera. Upon requests from the participant, to further examine the details and small nuances of the dosing day experience, (parts of) the filmed dose session can also be viewed together with the participant during the integration sessions. The recordings will be analyzed with established instruments to classify and quantify in-session verbal behaviors. For some of the sessions, qualitative content analysis will also be performed. The SaS will be identical for those randomized to psilocybin or niacin active placebo.

6.2 Justification for Selected Aspects of the Study Design

6.2.1 Use of a single psilocybin dose

Traditional psychotropic agents used to treat MDD require continued administration for maintenance of clinical efficacy[88], and single doses of novel agents such as ketamine that produce an antidepressant effect that outlasts the medication’s direct biological activity rarely induce a therapeutic response that lasts more than a week[89]. On the other hand, available evidence suggests that a single dose of psilocybin when administered under the SaS protocol produces an antidepressant effect durable enough to justify this treatment modality as a significant addition to the antidepressant armamentarium. For example, in a study conducted at Johns Hopkins University (Hopkins)[46], 24 participants with cancer and clinically significant depression (mean HAM-D score = 22.84 [0.97]) randomized to receive a single dose of psilocybin demonstrated remarkably high rates of symptomatic response (79%) and remission (71%) six months post-intervention without requiring additional pharmacologic intervention. A similarly high rate of response at 26 weeks post-intervention was seen in 11 participants randomized to psilocybin in the study of depression/anxiety in patients with cancer conducted at NYU (BDI response = 82%; HADS Depression response = 82%)[1]. Finally, a 12 participant open trial of two doses of psilocybin separated by a week in otherwise medically-healthy patients with TRD reported BDI response rates at 3 months post- intervention as 58% and 3 month post-intervention remission rates as 42%[2]. Taken together, these data suggest that clinically-meaningful antidepressant efficacy for single dose (or two doses with one being less than half the dose proposed for the current study) can be observed out to 6 months post-intervention in the context of cancer, and at least 3 months post-intervention in medically healthy individuals with MDD.

6.2.2 Psilocybin and Niacin Dosing

This study will compare the depression-relevant behavioral effects of a single 25 mg oral dose of psilocybin with a single 100 mg oral dose of a niacin active placebo. A 25 mg oral dose of psilocybin was selected based on data from an open trial of psilocybin in otherwise medically- healthy patients with TRD showing that this dose produced a robust and sustained antidepressant response. Moreover, this dose is within range of the absolute dosages received in previous trials of single-dose psilocybin for depression and anxiety in patients with cancer (actual mean dose based on weight of patients at each site was 23.4 mg for the study conducted at Hopkins (Roland Griffiths, PhD, *personal communication with Rob Barrow (RB)*) and 21.4 mg for the study conducted at NYU (Steve Ross, MD, *personal communication with RB*). Justification for a fixed dose, as opposed to a weight-based strategy, comes from data pooled from four studies (N =141) in which participants of varying weight were administered 30 mg/70 kg psilocybin, and from data from six studies of individuals of varying weight who received a dose of 23 mg (N = 21) (Garcia-Romeu A, Johnson MW, Barrett FS, Carbonaro TM, Griffiths RR. Psilocybin effects in humans: absolute vs. body weight adjusted dose, *in preparation).*

In both cases, no significant effect of weight was seen on any acute psychological effects of psilocybin, suggesting that a fixed-dose strategy should be as effective as weight-based dosing while significantly simplifying future medication delivery. Moreover, a fixed dose of 25 mg of psilocybin produced large effect size reductions in depressive symptoms in medically-healthy patients with TRD.

The selection of 100 mg as the dose for niacin is based on several factors. First, it is above the 75 mg dosage that reliably induces skin flushing that may provide a physiological effect to help blind the study (https://articles.mercola.com/sites/articles/archive/2017/03/06/is-niacin-flush- dangerous.aspx). Second, it is less likely to induce the severity of flushing that was frequently seen when a dose of 250 mg was used to blind the NYU phase 2 study of psilocybin in patients with cancer and depression/anxiety[1]. This level of flushing produced uncomfortable physical sensations that might induce a dysphoric state that would artificially inflate psilocybin-placebo differences. In addition, the degree of flushing induced by a 250 mg dose was occasionally patently apparent to observers (i.e. Facilitators), which may have worked against its use as a blinded active placebo.

6.3 Planned Duration of Study

The planned maximum study duration for each participant will be approximately one year, with variation primarily dependent on the length of the screening period, the number of days between Baseline and dosing, and the visit windows provided for each post-dose assessment.

The screening period will last between 7 and 35 days to allow for scheduling and completion of all screening activities.
The intervention session will occur following completion of the preparation session but no later than 7 days after preparation.

The period between the dosing session and completion of the final post-dose assessment in phase A will be on average 6 weeks (42 days), with some variation allowed as a result of each post-dose session having a window for completion. The follow-up phase (B) will continue until 365 days after treatment.

Time schedule: from Web-Screening-day to day 16

Activity	Dur. (min)	W-S	S-Day (-35- -7)	MRI1 (S-day- -7)	-10 (MRI1-1 - -7)	-3(-7 - -2)	-2	-1	0	1	2,3,4,5,6,7	8(-1/+2)	15 (+1)
Psychiatric interview	20		x										
AUDIT	5	x											
DUDIT	5	x											
Height, weight	5		x										
Confirm eligibility	5	MD1											
Informed consent	10		MDs										
MADRS	10		MDs			MD_P			MD1			MD1	MD2
MINI	40		MDs										
Physical exam	15		MDs										
Referral ECG	20		MDs										
Referral blood & urine screen	20		MDs										
Confirm eligibility	5		MD1					MD1					
Booking LP and PET slot			MD1										
Urine tox and pregnancy	5					PET_N							MD_P[1]
MRI1	60			x									
MRI2	90									x			
BSB	5					PET_N							PET_N[1]
PET	120					MD_P							MD_P[1]
PET-spare day							x	x					
LP Biomarkers	60				MD2								MD2
MADRS-S (SRP, TC)	3	x				x			x	x	x	x	x
Adverse event interview	3								x	x		x	x

Activity	Dur. (min)	W-S	S-Day (-35- -7)	MRI1 (S-day- -7)	-10 (MRI1-1 - -7)	-3(-7 - -2)	-2	-1	0	1	2,3,4,5,6,7	8 (-1/+2)	15 (+1)
GAD-7 (SRP, TC)	1					x			x			x	x
EQ-5D (SRP, TC)	1					x			x			x	x
SDS (SRP, TC)	1					x			x			x	x
T-Exp (SRP)	1			MD_M									
Preparation session	60							F1;F2					
WAI-S (SRF)	2							MHS_N				MHS_N	
WAI-S T (SRP)	2							F1;F2				F1;F2	
Dosing session	480								MHS_N F1;F2				
MEQ (SRP)	3								F1;F2				
EBI (SRP)	1								F1;F2				
CEQ (SRP)	2								F1;F2				
SPIR (SRP)	1								F1;F2				
BP & HR	5								F1;F2				
PDM	5								MHS_N				
PDRF	5								MHS_N				
Integration session	60									F1;F2		F1;F2	F1;F2[2]
AAQ-II & ALIVE (SRP)	1							F1;F2				F1;F2	F1;F2
MLQ & SWLS (SRP)	1							F1;F2				F1;F2	F1;F2
LEQ (SRP)	3							F1;F2				F1;F2	F1;F2
CGI-S	1								MD1			MD1	MD2
CGI-I	1											MD1	MD2
T-Eval (SRP)	1									MD_M			MHS_N
Total duration/day (min)		***13***	***145***	***61***	***60***	***146***		***74***	***480***	***157***	***3***	***90***	***277***
Visit at research site (v)			***v***	***v***	***v***	***v***		***v***	***v***	***v***	***v***	***v***	***v***

Time schedule: Day 43-365

Activity	Dur. (min)	42(±3)	72(±5)	102 (±5)	132 (±5)	162 (±5)	192((±5)	223 (±5)	253 (±5)	283 (±5)	313 (±5)	343 (±5)	365 (±14)
MADRS	10	MD1											MD1
MADRS-S (SRP, TC)	3	x	x	x	x	x	x	x	x	x	x	x	x
GAD-7 (SRP, TC)	1	x	x	x	x	x	x	x	x	x	x	x	x
EQ-5D (SRP, TC)	1	x	x	x	x	x	x	x	x	x	x	x	x
SDS (SRP, TC)	1	x	x	x	x	x	x	x	x	x	x	x	x
AAQ-II & ALIVE (SRP)	1	MD1											MD1
MLQ & SWLS (SRP)	1	MD1											MD1
LEQ (SRP)	3	MD1											MD1
CGI-S	1	MD1											MD1
CGI-I	1	MD1											MD1
Adverse event interview	3	x											
Total duration/day (min)		*26*	*6*	*6*	*6*	*6*	*6*	*6*	*6*	*6*	*6*	*6*	*23*
Visit at research site (v)		*V*											*V*

Dur = approximate duration for patient (minutes), W-S = Web screen, S-Day = Screening day, MD1 = Study physician, Medical doctor and study coordinator, MD2=Medical Doctor at MHS, MDs = Screening responsible MD, MD-M = MR-responsible MD, MD_P = PET responsible MD, MHS = Mottagningen för hjärnstimulering, MHS_N = Dedicated nurse from MHS, SRP = Self rating by patient, SRF = Self rated by facilitator, TC = TakeCare, BP = Blood pressure, HR = Heart rate, T-Exp = Treatment Expectation, T-Eval = Treatment Evaluation, PDRF = Post dose release form, PDM = Post dose monitoring, BSB = Blood sampling biomarkers. X = completed by patient. [1]PET2 is done day on 15±7.[2]after LP2 and PET2

Table 1: Schedule of assessments

7. PARTICIPANT POPULATION

7.1 Inclusion and Exclusion Criteria

7.1.1 Inclusion Criteria

Individuals eligible to be randomized in this protocol are those who meet *all* of the following criteria:

1. Are 20 to 65 years old at the time of written informed consent at the In-Person Screening visit
2. Are able to read, speak, and understand Swedish
3. Are able and willing to adhere to study requirements, including attending all study visits, preparatory and follow-up sessions, and completing all study evaluations
4. Are able to swallow capsules
5. Women of childbearing potential (WOCBP) must agree to practice an effective means of birth control throughout the duration of the study, from Screening through the Day 42 assessment
6. Meet ICD-10 criteria for a diagnosis of remitting major depressive disorder and are currently experiencing a major depressive episode of
   a) at least a 30-day duration at the time of the Screening
   b) less than 5 years at time of Screening
7. Have sustained moderate-severe depression symptoms at Screening and Baseline, as defined by a Screening MADRS total score ≥ 22 and ≤30% and ≤7 point improvement (i.e. decrease) in MADRS total score from web-screening to screening visit (assuming 3 points on item 1 at web screening).

9. Have an identified support person
    a. Agree to be driven/accompanied home (or to an otherwise safe destination) by the support person, or another responsible party, following dosing

7.1.2 Exclusion Criteria

Individuals not eligible to be randomized in this protocol are those who meet *any* of the following criteria:

1. Women who are pregnant, as indicated by a positive urine pregnancy test at Screening or Baseline. Women who intend to become pregnant during the study or who are currently nursing.
2. Current depressive episode lasting >5 years
2. Unwilling or unable to discontinue formal psychotherapy
3. Ongoing antidepressant drug treatment
4. Have previously during the current episode received the following non-medication treatments:
    a. deep brain stimulation (DBS)
    b. vagus nerve stimulation (VNS)
5. Currently receiving electroconvulsive therapy (ECT) or transcranial magnetic stimulation (TMS)
6. Unable or unwilling to discontinue any current medications that are known uridine diphosphate (UDP) or glucuronosyltransferase (UGT) enzyme modulators (eg valproate)
    o Note: Any prohibited agents must have been stopped at least 5x the elimination half-life of the specific drug at the time of Baseline. See Appendix A for a full list of prohibited medications.
7. Report psychedelic substances use ever
    o Note: Psychedelic substances include psilocybin, Lysergic acid diethylamide (LSD), mescaline (and natural products containing mescaline including peyote and San Pedro cactus), N,N-Dimethyltryptamine (DMT), natural products containing DMT including ayahuasca and 5-Methoxy-N,N-dimethyltryptamine (5-MeO-DMT), ibogaine, 2C compounds, 3,4-methylenedioxy- methamphetamine (MDMA), methylone or other psychedelics.
8. Have the following cardiovascular conditions:
    a. coronary artery disease, congenital long QT syndrome (prior diagnosis), cardiac hypertrophy, cardiac ischemia, congestive heart failure, myocardial infarction (prior diagnosis);
    b. tachycardia (defined as heart rate > 100 beats per minute);
    c. a clinically significant Screening ECG abnormality (e.g., atrial fibrillation);
        o Note: A QTcF interval > 450 milliseconds is considered a clinically significant ECG abnormality
    d. artificial heart valve; or
    e. any other significant current or history of cardiovascular condition, based on the clinical judgment of study physician, that would make a participant unsuitable for the study
9. At Screening or Baseline have elevated blood pressure as defined as:

   a. Screening blood pressure SBP >135 mmHg or DBP > 85 mmHg on three separate readings; or
   b. Baseline blood pressure SBP >140 mmHg or DBP > 90 mmHg on three separate readings
10. Have a history of stroke or Transient Ischemic Attack (TIA)
11. Have moderate to severe hepatic impairment, as indexed by a Child-Pugh score ≥ 7
12. Have epilepsy
13. Have insulin-dependent diabetes
   - Note: Participants who are taking oral hypoglycemic agent and have a history of hypoglycemia requiring medical intervention will be excluded
14. Are unable or unwilling to adhere to the following medication requirements:
   a. Agree to suspend sildenafil (Viagra®), tadalafil, or similar medications at least 72 hours prior to dosing
   b. If taking any supplement containing >20 mg of niacin, agrees to suspend use for the duration of the study
15. Have a positive urine drug test including Amphetamines, Barbiturates, Buprenorphine, Benzodiazepines, Cocaine, Cannabis, Methamphetamine, MDMA, Methadone, Opiates (Morphine, Oxycodone), Phencyclidine (PCP), and Tetrahydrocannabinol (THC). Exceptions are made for prescribed Benzodiazepines (stable dose for sleep or anxiety).
   - Note: Benzodiazepine medications for sleep and non-benzodiazepine sleeping medications will be allowed to continue through the study period for participants who have been on a stable dose of such a medicine for at least 6 weeks prior to Screening, as determined during review of concomitant medications
   - Note: Participants using cannabis, including legal cannabis, for any purposes will be excluded
   - Note: Participants who are taking prescription maintenance methadone or buprenorphine naloxone will be excluded
   - Note: Prescription opiates must have been stopped at least 5x the elimination half- life of the specific drug at the time of inclusion, as confirmed with a negative urine drug screen.
16. Nicotine dependence that would disallow an individual to be nicotine free for the 7-10 hours during the dosing period
17. Meet ICD-10 criteria for schizophrenia spectrum or other psychotic disorders, including MDD with psychotic features (except substance/medication-induced or due to another medical condition), or Bipolar I Disorder, Bipolar II Disorder and bipolar disorder NOS.
   - Note: Participants with any lifetime diagnosis of schizophrenia spectrum or other psychotic disorders will be excluded
18. Meet ICD-10 criteria for antisocial personality disorder
19. Meet ICD-10 criteria for a moderate or severe alcohol or drug use disorder (excluding caffeine)
   - Note: Participants with a diagnosis of alcohol or drug use disorder within the past 12 months will be excluded
20. Have presence of any psychiatric condition or symptom judged by the PI (or designee) to be a more significant clinical problem than MDD for the participant.
21. Have a first-degree relative with schizophrenia spectrum or other psychotic disorders (except substance/medication-induced or due to another medical condition), or Bipolar I Disorder

22. Have a psychiatric condition judged to be incompatible with establishment of rapport with the Facilitators or safe exposure to psilocybin
23. Report the following suicidal ideation or suicidal thoughts defined as:
    a. Have a score of ≥ 5 on Item 10 (suicidal thoughts) of the central-rater or computer administered MADRS at Screening or Baseline; or
    b. Have any suicidal ideation or thoughts, in the opinion of the study physician or PI, that presents a serious risk of suicidal or self-injurious behavior at any time prior to randomization
24. Have any suicidal ideation or thoughts, in the opinion of the study physician or PI, that presents a serious risk of suicidal or self-injurious behavior
25. Have any physical or psychological symptom, medication or other relevant finding at Screening or Baseline, based on the clinical judgment of clinical/medical study personnel, that would make a participant unsuitable for the study.
26. Have an allergy or intolerance to any of the materials contained in either drug product
27. Have Hepatitis B, C or HIV
28. Have one or more pathological blood test results as defined in 5.6.3 (as determined by a study physician; with the exception of CRP).

7.2 Justification for Inclusion/Exclusion Criteria

Provided below is a justification for criteria that are either not self-evident or differ in some way from standard practice in clinical trials in patients with MDD.

7.2.1 Outside Psychotherapy

Participants will only be eligible for study enrollment if they agree and are judged able, from a safety perspective, based on the opinion of a study psychiatrist, to suspend any ongoing outside psychotherapy for the duration of study involvement phase A (ie 42 days from dosing). The rationale for suspending outside therapy is to allow for adequate evaluation of the study intervention. Suspending outside psychotherapy for the duration of the study is similar to the need for participants not to be taking outside psychotropic medications (e.g. antidepressants) during the study period. As with continuing pre-existing antidepressant medications, allowing participants to continue pre-existing outside psychotherapy would potentially confound the ability to unambiguously interpret study findings regarding whether monotherapy with psilocybin delivered in a therapeutic context shows an antidepressant effect when compared to placebo. This is true for several reasons. First and foremost, continuance of outside psychotherapy would not be "per protocol" and hence would not be standardized, nor would it be randomized, leading to the possibility of it being unequally distributed across groups. Moreover, it is completely unknown whether outside psychotherapy would potentiate or diminish antidepressant responses to psilocybin. Starting with phase B, in the presence of clinically relevant MDD symptomatology, outside psychotherapy as well as antidepressant drug treatment may be introduced, as it is included in the exploratory outcome measures.

7.2.2 Benzodiazepines

Benzodiazepine medications for sleep and non-benzodiazepine sleeping medications will be allowed for participants who have been on a stable dose of such a medicine for at least 6 weeks prior to Screening. These medications are allowed because they have no psychotropic effects that might confound any potential differential antidepressant signal from the

psilocybin vs. niacin placebo. On the other hand, requiring discontinuation of these sleeping agents prior to study drug dosing would likely disrupt stable sleep patterns that were part of the pre-treatment depressive symptom profile, and by doing this might interfere with potential antidepressant signals from the study drugs. Because these agents are used at bedtime, they will be out of participants' systems prior to study drug dosing and thus should not interfere with either psychodynamic or pharmacokinetic properties of psilocybin.

7.2.3 Age range

The age range for the current study is typical for most antidepressant studies, with enrollment limited to individuals 20 years of age and older, but not older than 65 years. The rationale for excluding individuals with MDD younger than 20 is twofold. First, although not explicitly written in the current European Council Directive 2013/59/Euratom of 5 December 2013 (https://eur-lex.europa.eu/eli/dir/2013/59/oj), the Regional Radiation Safety Committee of Karolinska University Hospital Stockholm has hitherto recommended the age of included subjects to be 20 years or older. Second, younger subjects are at an increased risk of demonstrating a bipolar disease course with the passage of time and bipolar disease is believed to be contraindicated for the use of psilocybin[90]. There are no data to support the use of psilocybin in patients with bipolar disorder, nor data on how likely psilocybin would be to induce a manic episode in patients with a bipolar diathesis. In addition to this particular concern regarding incipient or misdiagnosed bipolar disorder in those younger than 20, individuals in this age group are in general at increased risk for adverse psychological reactions to antidepressants.

This study will also exclude individuals older than 65 years of age. In addition, as with individuals younger than 20, older adults often differ from younger adults in factors associated with their depression and in their intervention response. For example, depression in older adults, especially when it is new onset, is significantly associated with the subsequent development of dementia[91]. And studies suggest that older adults are less likely than younger adults to respond to pharmacological treatments for MDD when depression is associated with brain changes also associated with dementia[92,93].

Additionally, over the course of its long-term clinical development phase, over a thousand participants have received psilocybin under controlled conditions in clinical settings for various indications (including healthy control subjects), with subsequent results published in peer- reviewed journals[94]. The modern-day trials, enrolling approximately 165 adult participants, include open-label, dose-escalating studies, as well as randomized, double-blind placebo-controlled trials, and enrolled both healthy volunteers and various subpopulations with differing indications[1,2,46]. Multiple ongoing studies are continuing this exploration. All these studies had or currently have approval to enroll participants > 55 years (age ranges from 18-80 years). Furthermore, of the approximate 165 participants receiving study drugs in completed trials, 73 adult participants were > 55 years (Roland Griffiths, PhD; Matthew Johnson, PhD; Robin Carhart-Harris, PhD; Francisco Moreno, MD, Paul Hutson, PharmD; Stephen Ross, MD; Charles Grob, MD, *personal communication with RB*). To date, no Serious Adverse Events attributed to the study drug were reported for any participant in these trials, regardless of age.

For these reasons, selecting the 20-65 year age range maximizes the generalizability of any potential positive findings while reducing risk and constraining biological/phenomenological variance that might decrease the power to detect clinically-

relevant effects. Should results from the proposed study be promising, separate trials of psilocybin intervention in younger adult and geriatric populations would be warranted.

7.2.4 Medically Healthy Participants

The current study will enroll participants in good general overall medical health. While this somewhat limits the potential generalizability of any positive findings given the high comorbidity between mood disorders and medical disease, there are two primary reasons for choosing this strategy. First, medically-ill participants often have complex patterns of depressive pathogenesis born of the psychological stress of serious disease, but also likely from the direct physiological effects of the disease state itself, most notably the chronic immune activation that accompanies a wide range of medical illnesses[95]. Given this, separate studies are indicated to examine the potential antidepressant effects of psilocybin in patients with MDD and significant medical comorbidity.

7.2.5 Psychiatric Symptoms

Study inclusion/exclusion criteria include several exclusions related to psychiatric symptoms and conditions. Participants with active suicidal ideation with intent to act are excluded because these individuals require immediate psychiatric intervention with currently approved therapeutic modalities. The safe study of such individuals would require an inpatient setting and the current study will be conducted on an out-patient basis. Similarly, individuals with a history of medically significant suicide attempt will be excluded given evidence that a recent history of suicide attempts increases the short-term risk for subsequent suicide attempts.

Individuals who are experiencing depression in the context of conditions characterized by a heightened risk for psychotic features, including schizophrenia and bipolar I and II disorder, are also excluded, including individuals with first-degree relatives with these conditions. These exclusions are based on the fact that not enough is known regarding the risks of a psychedelic experience in individuals with a vulnerability to psychosis to justify their inclusion in the current study. At the least, such individuals might have experiences occasioned by psilocybin that would differ markedly in their character and effects from experiences that would be more typical for individuals without psychosis risk.

As is common practice in phase 2 studies of new antidepressant modalities, individuals with active drug/alcohol abuse are excluded, as are those who have only recently achieved sobriety. Justification for these exclusions comes from evidence that antidepressant treatments are less effective in those who are actively abusing drugs/alcohol and from the fact that relapse rates are high during early sobriety, increasing the risk of a return to substance use during the study.

Because psilocybin administered under the SaS requires the development of rapport between participant and his/her intervention Facilitators, participants will be excluded prior to intervention if it is judged by the Facilitators (with agreement from a study clinician) that a given participant is not demonstrating a minimal degree of involvement/engagement with the intervention team.

Also, because this study focuses on MDD, participants with comorbid psychiatric conditions (e.g., obsessive-compulsive disorder, panic disorder, ADHD, Borderline Personality Disorder) that are judged at Screening to be a more significant source of distress/impairment than the MDD will be excluded from study participation. Comorbid psychiatric disorders other than those specifically disallowed (i.e. psychotic disorders, bipolar I and II disorder,

antisocial personality disorder) will be allowed if it is determined based on assessment at Screening that the participant's primary condition is MDD.

7.2.6 Medication Exclusions

In addition to these medical and psychiatric inclusion/exclusion criteria, participants will be required to abstain from medications/supplements that might interact with, or impact the experience of, psilocybin administration. This strategy is proposed both to enhance safety (i.e. by limiting co-exposure to medications that might increase potential serotonin-related side effects of psilocybin) and to reduce biological heterogeneity of participants entering the intervention session.
Patients with ongoing antidepressant treatment are excluded to avoid difficulties in interpretation of results from psilocybin treatment. This exclusion is also expected to result in a cohort more representative of the future target patient group, assuming approval of psilocybin as a depression treatment. The time that patients should be off treatment is at least 30 days prior to screening.

7.2.7 Blood Pressure

The Screening and Baseline blood pressure criteria are in place for both safety and feasibility reasons. First, the American College of Cardiology and American Heart Association recently lowered their criteria for Stage 1 hypertension from SBP140 mmHg/ DBP 90 mmHg to SBP 130mmHg/DBP 80 mmHg, citing strong observational data to support this change[96]. By lowering the Screening BP criteria to SBP > 135 mmHg or DBP > 85 mmHg, the study will in effect recruit a healthier population that is at less risk for cardiovascular events. Second, due to the nature of the study, it is likely that some percentage of participants will present on the dosing day with vital signs elevated above Baseline values due to anxiety over the upcoming procedure (i.e. "white coat syndrome"). By reducing the Screening criteria to 135/85 mmHg, it is expected that the number of participants who fail to meet the dosing day (pre-dose) BP criteria of <140/90 mmHg due to elevated BP resulting from anxiety will be reduced. This change will significantly reduce the time burden and psychological stress on participants who would otherwise qualify but fail to meet BP criteria on the dosing day. Importantly, by setting more stringent BP parameters at screening we will also reduce the number of participants who will unnecessarily taper off from psychotropic agents and psychotherapy prior to dosing day should they be discontinued due to "white coat syndrome" elevation of BP on dosing day.

8. SCREENING PROCESS AND PROCEDURES

Study screening procedures and their timing are summarized in the Schedule of Assessments (Table 1). Participants will be provided with visit reminders. Adherence to the study design requirements, including those specified in the Schedule of Assessments, is essential and required for study conduct.

8.1 Recruitment

Participants for the study will/may be recruited from the following sources: 1) a study specific website, 2) relevant clinical programs at Norra Stockholms Psykiatri, SLSO; 3) via advertisements on the web, and 4) via relevant national clinician-focused listservs and patient-advocate listservs. All advertisements will be approved by the IRB prior to posting. As this study will require intensive study recruitment measures and new and various

recruitment opportunities are found often, there may be instances of recruitment which do not fall under the above categories. However, prior to initiation of any recruitment activities, approval will be obtained from the IRB.

8.2 Study Recruitment Website and Pre-Screener Questionnaire

A clinical research recruitment website will serve as the anchor for the patient recruitment efforts and as the hub for study digital recruitment activities. The website contains basic educational content about the clinical trial, depression and psilocybin. The study profile page provides key study details for prospective participants. Study profile page visitors who are interested in participating in the study may complete an eligibility pre-screener questionnaire.

Pre-Screener Questionnaire
The pre-screener includes questions regarding demographics and availability, medical and mental health questions, and prior psychedelic and other drug use including alcohol. It also includes a waiver of documentation of consent as it does not present more than minimal risk of harm to participants and involves no procedures for which written consent is normally required outside of the research context. During the pre-screener, participants will be informed, via the website, that answering questions is voluntary and that any information shared will be kept confidential. Identifiable contact information will be encrypted.

8.3 Telephone Screen

Prospective participants will be prescreened by telephone to learn if they meet basic eligibility criteria. The Telephone Screen will include questions similar to the pre-screener regarding demographics and availability, medical and mental health questions, and prior psychedelic and other illicit drug use.

If at any time during the telephone screen process a potential participant discloses suicidal ideation, he or she will be immediately encouraged to call 112.

If the initial telephone screen suggests that the participant is eligible, an In-Person Screening visit will be scheduled. If the participant does not qualify or decides not to participate the reason will be documented in the Electronic Data Capture (EDC) system with all identifiable information encrypted.

Telephone Screen information will be verified at the In-Person Screening visit prior to written informed consent.

8.4 Written Informed Consent

For this trial, written informed consent will occur at the initial In-Person Screening visit. Study participants will be considered enrolled in the study when they provide written informed consent. All individuals who sign an ICF will be assigned a participant ID number in the EDC, which will be assigned sequentially. Written informed consent will be obtained in person from all participants before undergoing any study procedures. Prisoners, pregnant women and mentally impaired persons will not be included. Competent persons meeting other eligibility criteria will be welcomed, regardless of race, gender, ethnicity, religion or

socioeconomic status. Additionally, students, employees, patients or family members affiliated with the PI(s) will not be enrolled, and family members of any study team member will be prohibited from participating in this study.

The following consent process will be adhered to for each participant:

- The PI or a qualified representative will explain the screening process and nature of the study to the participant and answer all questions.
- Participants must be informed that their participation is voluntary and that he/she may withdraw from the study at any time, for any reason.
- Participants will be provided adequate time for review and discussion prior to his/her making a decision about participation in the study, including the option to take the information home to discuss with family prior to making a decision.
- Participants will be required to sign a statement of informed consent that meets the current requirements of ICH guidelines, and the Institutional Review Board (IRB).
- Participants will be encouraged to ask any questions at any time for any reason, and to seek outside counsel when appropriate.
- To verify informed consent was obtained before the participant was enrolled in the study, the ICFs will contain the date and time written consent was obtained. The authorized person obtaining informed consent must also sign the ICFs.
- Participants must be re-consented to the most current version of the ICFs during their participation in the study, per institutional IRB requirements, and they will be informed of any changes to the consent form.
- A copy of the ICFs must be provided to the participant.

The ICF and any other written participant information to be provided to participants will be revised whenever important new information becomes available that may be relevant to the participant's consent. Any revised ICF and written participant information will receive IRB approval before being implemented. The participant will be informed of any changes and re- consented with the current version of the consent form and provided a copy at their next visit. Communication of this information to the participant will be documented.

8.5 In-Person Screening Visit (7-35 days prior to dose)

After preliminary eligibility is determined via the Telephone Screen, an in-clinic screening will occur at Norra Stockholms Psykiatri. The purpose of the In-Person Screening visit is to confirm eligibility for the study. Upon signing the Informed Consent Form (ICF), the potential participant may commence study-related screening activities. No study related procedures, other than the study pre-screening questionnaire and telephone screen, will occur prior to obtaining written informed consent.
Screening will take 7 to 35 days to allow for full medical and psychiatric evaluation. The purpose of the Screening visit is to evaluate medical and psychiatric appropriateness for the study. If at any point the participant is found to be ineligible, the screening process may be discontinued, and any remaining procedures will not take place.

8.5.1 Inclusion/ Exclusion Criteria Confirmation

Following the completion of all Screening medical and psychiatric assessments, the PI will confirm participant eligibility on an ongoing basis until dosing.

8.5.2 Magnetic Resonance Imaging (MRI)

After informed consent and before Lumbar Puncture (day 7-35 before dosing), MRI1 will take place at MR-centrum Solna. The visit will take approximately 1 hour and will include structural functional sequences including a set of tasks (see 14).

8.5.3 Lumbar puncture

The first lumbar puncture (LP; day 7-35 before dosing) for acquisition of Cerebro Spinal Fluid (CSF) will be performed at a separate visit. This is done after written or verbal report of the MRI examination within the screening process has confirmed absence of signs of increased intracerebral pressure. The LP will take place at Norra Stockolms Psykiatri, Mottagningen för hjärnstimulering or the PET center, or another medical site and will be performed by a registered physician according to standard procedures in local anaesthesia. All together at least 5 ml CSF will be sampled.

8.5.4 PET1

The first [11C]UCB-J PET examination will take place after LP and before dosing (1-7 days). The examination will be performed at the PET-center at Karolinska Solna and the visit including preparations will take approximately 3 hours. At the visit a rater based MADRS and blood sampling will be performed. Urine samples for toxicology and, if applicable, pregnancy, will be analysed prior to the PET examination.

9. PREPARATION, RANDOMIZATION, DOSING AND FOLLOW UP PROCEDURES

This section outlines the purpose and content of all preparation, randomization, dosing and follow up study procedures. Participants will be provided with visit reminders. For the timing and frequency of assessments at each visit see Table 1.

9.1 Preparatory Sessions with Clinical Facilitators (1 day prior to dose)

Following successful completion of Screening, participants will be assigned a Lead Clinical Facilitator and a Co-Facilitator. Both Facilitators will remain with the participant throughout the preparatory, dosing, and integration sessions.

The main objective during preparatory session is to build rapport and therapeutic alliance between the participant and Session Facilitators, as support for the participant as they navigate the dosing session. Participants will meet with both the Facilitators, who will also attend the participant's dosing session. Whenever possible, these meetings will occur in the room in which dosing will take place, so participants can develop a level of comfort and familiarity with the intervention location. Consistent with previous psilocybin protocols, the participant's life history and current situation in life will be reviewed, and intentions and expectations for the intervention session will be discussed.

9.1.1 Post Preparatory Session Assessment (1 day prior to dose)

This assessment can occur on the same day as the preparatory assessment but must be performed after all preparatory session activities are complete. Before dosing, Facilitators will communicate with the PI or and Study Physician to discuss overall appropriateness for dosing. If the Facilitators, PI or Study Physician believes the dosing session is contraindicated, the session will be cancelled or postponed (Table 1; confirm eligibility#2).

9.2 Randomization

Randomization will occur for participants who have been determined to be eligible for the study and will occur the morning prior to dosing. Following this, participants will be randomized to one of two intervention groups. Participants are randomized in blocks in a 1:1 ratio in a blinded fashion to the psilocybin group or the active control group. The block size will not be shared. Randomization will be done at the site by strictly following a randomization list in sequential order. If a patient is replaced the new patient will be allocated a randomization number with the same treatment while keeping the double blinded allocation. This will be performed by having back-up blinded treatment allocations for each subject in sealed envelopes. These will only be used and opened following withdrawal. Importantly, the allocated treatment will remain blinded for both the withdrawn and replacement subject as well as for the study team.

The master randomization code will be maintained in a secured location until the time of unblinding. Individual emergency envelopes will be available at the site in case the investigator for safety reasons has to break the blind for a specific patient.

9.3 Day 0: Dosing Session

Participants will be asked to report to the research site, Mottagningen för Hjärnstimulering, NSP, ST Görans sjukhusområde, in the morning. Sessions are expected to last 6-10 hours.

9.3.1 Pre-Dose

Upon arrival, and prior to the dosing, the following assessments will occur:

1. Study personnel will inquire about any possible changes in health
2. Study personnel will inquire about any change in concomitant medication use and adherence.
3. A urine drug test for drugs of abuse with be performed
4. Urine pregnancy testing (women of childbearing potential only) will be performed
5. The study coordinator will remind the participant of their agreement to the following during the dosing session, as previously agreed to during the preparation sessions (also see Manual for Clinical Facilitators for agreements):
    - The participant agrees to remain on-site for a minimum of 7 hours on their dosing day after ingesting the psilocybin or placebo capsule and all identified study personnel agree that the participant may safely leave the facility with their identified support person.

- o The participant agrees to call the site and/or 112 if he or she has suicidal thoughts or feelings throughout the duration of the study.

6. Pre-Dose vital signs (Blood Pressure and Heart Rate)

<u>Urine Drug Test Results</u>
A negative urine drug test will be required the morning of dosing prior to drug administration. The Study Physician will be notified of urine drug test results. A positive urine drug test will result in the dose being canceled with no option for future rescheduling. The patient will be withdrawn from the study.

<u>Pregnancy Test Results</u>
A negative urine pregnancy test (women of childbearing potential only) will also be required the morning of dosing prior to drug administration. The Study Physician will be notified of urine pregnancy test results. A positive pregnancy test will result in the dose being canceled with no option for future rescheduling. The patient will be withdrawn from the study.

<u>Pre-Dose Cardiovascular Monitoring</u>

Table 2: Pre-Dose Blood Pressure Monitoring

<table>
<tr><th colspan="3">Cardiovascular Monitoring (Blood Pressure): Pre-Dose</th></tr>
<tr><td colspan="3">Blood pressure will be obtained prior to dose administration[1]. Blood pressure assessments will be conducted by trained study personnel. The following safety parameters have been established:</td></tr>
<tr><th colspan="2">Result</th><th>Instructions</th></tr>
<tr><td>SBP < 140 mmHg and DBP < 90 mmHg</td><td>Acceptable</td><td>Proceed with dosing</td></tr>
<tr><td rowspan="3">SBP ≥ 140 mmHg or DBP ≥ 90 mmHg</td><td rowspan="3">High</td><td>Wait a minimum of 5 minutes and repeat blood pressure to determine if elevation is temporary. Participant should continue to remain recumbent.</td></tr>
<tr><td>If repeat BP is < 140 mmHg and < 90 mmHg, proceed with dosing</td></tr>
<tr><td>If three readings are elevated[2], consult with the study physician for further evaluation and follow up.</td></tr>
</table>

1 The initial reading should occur after the participant has been recumbent a minimum of 10 minutes

2 Dosing may be rescheduled at the discretion of the site PI or study physician. If > 7 days have elapsed since the Baseline visit, all Baseline measures must be repeated before dosing. If > 40 days have elapsed since the original Screening date the participant will be unable to participate in the study.

9.3.2 Dosing

Following successful completion and acceptable results of the above scheduled assessments the participant will be dosed. When the participant is ready, he or she will receive a single dose of the study drug (25 mg psilocybin or 100 mg niacin placebo), administered as a capsule and taken with approximately 200 ml of water. Regardless of the study drug received, the participant will be under observation for at least 7 hours following ingestion.

Following study drug ingestion, it is recommended that study staff entering the session room be limited to the two Facilitators. Only in the event of an emergency should additional study staff enter the room. The rationale for minimizing who enters the session room is to reduce distractions for the participant.

Session Facilitators

Facilitators will be licensed psychologists with experience in the psychological treatment of MDD. Both Lead and Co-Facilitators will have adequate training to identify safety issues during the participant's preparation, dosing, and integration sessions, and will have undergone specific Clinical Facilitator training.

For the majority of the dosing session, both Facilitators will be present in the session room and will attend to the participant. At least one Facilitator will always be present in the session room to continuously observe and evaluate the participant's physical and mental status (allowing for infrequent, short breaks; e.g. facilitator bathroom breaks). Continuous supervision provides a safety structure and ensures that the participant will receive reassurance and emotional support from the Facilitators; should he/she experience strong emotions or become anxious or agitated during the session. Specific dosing session safety monitoring, via eCRF, will be conducted at regular, pre-specified times through assessment of the following: nausea, acute physical or psychological distress, including self-harm, and self-reported anxiety. This is intended to provide Facilitators with an enhanced ability to monitor participant safety and communicate any observed symptoms to the Study Physician, who will determine if immediate clinical assessment is warranted.

Role of the Study Physician During Dosing

The on-call Study Physician is responsible for the overall safety of participants, and he/she will oversee the medical management of study participants during the dosing session, as needed.

- Availability during Dosing: The Study Physician will be on-call throughout the duration of the dosing session and the Study Physician or a psychiatry MD will need to be available to be onsite within approximately 5 minutes in the event of an emergency for medical/psychiatric assessment. An MD is responsible for administering rescue medications, should these be warranted.
- End of Dosing Day: The Study Physician or a designated study nurse will assess the participant in-person at the end of the dosing day to determine if he/she is ready to be released from the research site.
- Overnight: In case of need for support during the night the patient will be referred to the emergency ward of the Norra Stockholms Psykiatri (open 24/7/365). The Study Physician will be on-call following participant release and until the following morning when the participant returns for his/her integration session.

Rescue Medications Available During Dosing

In the unlikely event that a participant requires medication management for blood pressure, anxiety or psychosis, the study physician may use the eg the following medications per his/her medical discretion:

1. Nitroglycerin
2. Clonidine
3. Diazepam or oxazepam
4. Risperidone

The use of rescue medications to control symptoms will be at the judgment of the treating physician, therefore use of specific medications is not protocolized. The study site will supply the rescue medications, directly or via the medical emergency unit, Capio S:t Görans Hospital which is at the same hospital area as the study site. The date and time of medication administration, reason for administration, as well as the name and dosage regimen of the rescue medication must be recorded on the Concomitant Medications log. Use of psychotropic agents (e.g. diazepam, risperidone) will be on a single dose/time limited basis and hence will not fall under the study exclusionary criteria regarding chronic use of these agents prior to enrollment.

In the unlikely event of a medical or psychiatric emergency that cannot be safely managed by staff with reassurance or pharmacological intervention, the study physician will determine if the participant will be needed to receive treatment via the psychiatric emergency unit at the hospital area. If necessary, this treatment can be given according to the Swedish law regarding compulsory psychiatric treatment, *Lagen om Psykiatrisk Tvångsvård*.

Cardiovascular Monitoring During Dosing Session

Blood pressure and heart rate will be obtained at 30, 60, 90, 120 minutes, 4, 6, and 7 hours after drug administration. Participants will not be discharged until after the 7-hour blood pressure and heart rate assessments have been completed. Measurements must be collected within +/-10 minutes of the scheduled time point. Measurements will be conducted by trained study personnel. Blood pressure measurements should occur after the participant has been recumbent a minimum of 10 minutes.

Table 3: Blood Pressure Monitoring during Dosing

**Cardiovascular Monitoring (Blood Pressure): Dosing Session**	
The following Blood Pressure safety parameters have been established for the dosing session:	
**Result**	**Instructions**
SBP ≤ 170 mmHg and<br>DBP ≤ 95 mmHg	No additional follow up needed, take BP at next scheduled time point.
SBP > 170 and < 200 mmHg<br>or<br>DBP > 95 and <110 mmHg	Repeat every 5 minutes for a total of three readings over 15 minutes.<br>• If three or more readings are elevated consult with the

SBP ≥ 200 mmHg or DBP ≥ 110 mmHg	Consult with the study physician for further evaluation and follow<br>up. Medication and medication administration is at the clinical judgment of the Study Physician and is not protocolized. The following medication administration is a recommendation; however, the Study Physician may treat the participant based on his or her<br>own judgement and with the medications[1] available on site.<br>• The participant may be treated with sublingual nitroglycerin[1] 0.4 mg<br>• If blood pressure readings do not decrease below these thresholds after 5 minutes, the same dose of nitroglycerin will be administered<br>• A third dose of nitroglycerin[1] will be given after another 5 minutes, if readings remain elevated above these levels (maximum dose is 0.4 mg x 3)<br>• If blood pressure remains ≥200 systolic or ≥110 diastolic, at the judgment of the study physician, the participant will be treated with oral clonidine[1] (e.g., 0.1 mg) and the Study Physician will evaluate whether transport to the emergency room is necessary..

[1] Administration of any medications must be recorded on the Concomitant Medications form

Table 4: Heart Rate Monitoring during Dosing

**Cardiovascular Monitoring (Heart Rate): Dosing Session**	
The following Heart Rate safety parameters have been established for the dosing session:	
**Result**	**Instructions**
< 50 BPM	Repeat every 5 minutes for a total of 3 readings over 15 minutes.<br>• If three or more readings are below 50 BPM consult with the study physician
≥ 50 BPM and ≤ 110	No additional follow up needed, take HR at next scheduled time
> 110 BPM	Repeat every 5 minutes for a total of three readings over 15 minutes<br>• If three or more readings are elevated consult with the study physician

The final scheduled vital signs assessment is at 7-hours post-dosing, with the following safety parameters/ instructions.

The following safety parameters have been established for the End of Dosing Day (7-hour) blood pressure (BP) and heart rate (HR) measurements[1] .		
**BP Result**	**HR Result**	**Instructions**
SBP < 140 mmHg and DBP < 90 mmHg	< 100 BPM	No action needed. The participant may be released.

SBP ≥ 140 mmHg or DBP ≥ 90 mmHg	≥ 100 BPM	Participant should continue to remain recumbent.<br>Wait a minimum of five minutes and repeat measurement to determine if elevation is temporary. The measurement can be repeated up to three times, separated by a minimum of five minutes, with the participant remaining recumbent. Document any additional measurements.
SBP ≥ 140 mmHg or DBP ≥ 90 mmHg	≥ 100 BP	If there are three consecutive readings of SBP ≥<br>140 mmHg or DBP ≥ 90 mmHg, contact the Study Physician for further in-person evaluation and follow-up.<br>• The participant may be released if the Study Physician does not feel the elevated measurement is concerning.<br>• If the Study Physician feels the elevation is concerning, the participant will remain on site for further evaluation. If the Study Physician feels medication administration[2] is warranted, he/she can treat the participant with medications available at the site.<br>• If the participant responds to either waiting or medication administration, and the measurement decreases to a level the Study Physician does not feel is concerning, the participant may be released.<br>• If the participant does not respond and the Study Physician feels the elevation is concerning and warrants further evaluation, Study Physician will evaluate whether transport to the emergency room is necessary.

1Heart rate and blood pressure measurements should occur after the participant has been recumbent a minimum of 10 minutes
2Administration of any medications must be recorded on the Concomitant Medications form

9.3.3 Post-Dosing Release Procedures

Following the 7-hour vital sign assessment, if the participant appears to have returned to his/her baseline psychological and physiological state and expresses a readiness to begin the discharge process, the Lead Facilitator and Study Physician/designee will assess the participant for release. This assessment will confirm whether the participant is ready to safely leave the research facility accompanied by his/her support person (family or a trusted friend). This support person will have already been identified and briefed by Facilitators on basic guidelines for accompanying the participant home during preparation session #2. Self-

care activities and additional safety precautions will be reviewed with the participant before the participant and support person leave the premises.

If the participant is experiencing residual study drug effects, such as mild persistent sensorial distortions, he or she will be referred to the Psychiatric Emergency Unit for further observation.

While it is unlikely, if it is found that the participant is experiencing severe persisting physical or perceptual drug effects or is exhibiting signs of significant/severe emotional distress including suicidal ideation, or any other circumstance deemed an emergency per the Study Physician, the Study Physician will refer the participant to the Psychiatric Emergency Unit for further observation.

The participant will be given a post-dosing session summary form which they can use to record recollection of the dosing session before returning for Integration Session #1 on Day 1. This summary may be reviewed with the participant throughout the integration sessions.

9.4 Day 1: Post Dose, Integration Session #1

The Day 2 visit will occur in person. The purpose of the Day 2 visit is to assess the participant for safety and to allow for integration of the dosing session with the session Facilitators. This visit may take place at the MRI site for the convenience of the subject.

9.4.1 Day 1 MRI (+3 days)

At day 2 the MRI protocol will be repeated. The same sequences will be repeated (see paragraph 15.).

9.5 Day 8 (-1/+2 days) and 15 (+2 days)

Day 9 consists of collection of the primary study outcome as well as other safety and efficacy outcomes, and Day 16 allow for continued safety and efficacy follow-up. These visits will occur in person.

9.5.1 Day 15 post dose PET2 and biomarker sampling (±7 days).

At day16 PET2, LP2 and blood sampling 2 will occur at the PET-center. All procedures are identical to the #1 occasion.

9.5.2 Day 8 and 15 Integration Sessions #2 and #3

The purpose of these visits is to allow for further integration of the dosing session together with the dosing session Facilitators. These visits take place in person unless specific circumstances do not allow the participant to attend in person. In that case, video conferencing or phone calls will be arranged.

9.6 Day 42: Post Dose (±3 days)

This visit will occur in person at Mottagningen för Hjärnstimulering or Affektiva Mottagningen I/II.

Whenever possible, participants who have withdrawn from the study but agree to follow-up will also complete this assessment.

At this time point the patient will be referred for follow up treatment of MDD in accordance with regional treatment guidelines https://psykiatristod.se/regionala-vardprogram/depression

The patient will be kept blinded to the actual treatment received.

9.7 Monthly evaluations, visits from day 43 to 365 and

Each participant will report MADRS-S and EQ-5D data monthly (±5days) from day 43 to day 365.

9.8 Day 360 Post dose (±14 days) End of study visit

This visit will occur in person at Mottagningen för Hjärnstimulering or Affektiva Mottagningen I/II. Rating scales used: MADRS, GAD-7, EQ-5D, SDS, AAQII, MLQ, LEQ and CGI (table 1).

Whenever possible, participants who have withdrawn from the study but agree to follow-up will also complete this assessment.

9.9 Add on antidepressant treatment

From day 43, as determined by the patients physician (see 9.7), the patient shall be treated in accordance with regional guidelines: https://psykiatristod.se/regionala-vardprogram/depression

Information on any treatment that has occurred from day 43 to 365 will be extracted from patient records after day 365. Differences in duration (days) until initiation of antidepressant treatment between treatment groups will be analyzed.

9.10 Blinding Related to Delivery of Study Interventions and Collection of Outcome Data

In order to secure blinding for remaining patients in the study, no patient will receive information of the actual treatment received, with the exception of a serious adverse event, and then at the discretion of the PI or designee.

As with any interventions that produce noticeable psychological effects following administration, blinding psilocybin poses unique challenges.

Prior to start-up, all study site staff will receive extensive training in basic research principles of maintaining the study blind and ensuring only appropriate personnel have access to this information.

Session Facilitators, who will be in the room with participants throughout their dosing sessions, may become functionally unblinded as a result of participants demonstrating changes in behavior and/or speech suggestive of a psychedelic experience, or as a result of participants not demonstrating these types of changes. They will receive written information not to share their thoughts on the outcome of randomization with anyone.

9.10.1 Unblinding Procedure

Full blinding of study personnel and participants will be maintained until data lock at the conclusion of day 365 for all participants, follow-up study physicians and nurses.

Exceptions on an individual subject basis will be made in cases in which it is determined by the PI, the Medical Monitor or the Sponsor that unblinding of a participant's intervention assignment is required for participant safety. Anyone requesting individual unblinding prior to study-wide unblinding will first be referred to the site PI. The site PI must notify the medical monitor of the request and discuss the circumstances surrounding the request. The medical monitor will notify the Sponsor of the request and if unblinding occurred. Attempts should be made to maintain the blind of the investigators prior to the study-wide unblinding. Unblinding during the study will be recorded on the Protocol Deviation Form. All instances of unblinding must be reported to the site IRB and the Sponsor.

9.10.1.1 Interim analysis

After day 43 visit is completed for the last patient, data from phase A supporting the primary and secondary objectives will be made available to the statistician for analysis. No clinical staff, or PI, who may have future contact with the participants before day 365 will be unblinded. Aggregated results will be presented in a Clinical Study Report but individual data will not be listed.

9.11 Early Termination Visit

Should a participant terminate early, including Prep Phase Terminations and participants who are randomized and later terminate or are terminated for safety reasons, the End of Study visit (day 365) assessments will be completed to the extent possible within 14 days. The End of Study Plan will be discussed at the termination/End of Study visit. An End of Study visit will not be conducted for Screen Failures.

9.12 Unscheduled Visits

Unscheduled visits are any visits conducted to perform additional procedures other than regularly scheduled visit procedures, including additional integration sessions, if needed. Procedures performed will be based on the clinical judgment of the PI, Study Physician, or session Facilitators. Procedures and results will be recorded as an Unscheduled Visit.

10. STUDY DISCONTINUATION AND COMPLETION CRITERIA

10.1 Screen Failures

Screen Failures are defined as participants who are deemed ineligible either at the in-person Screening or Baseline assessment. Screen failures may fail to meet one or more Inclusion Criteria and/or may meet one or more Exclusion Criteria or withdraw consent. Screen Failures may be identified by various ways, including review of medical history, assessments, measures, laboratory results, or conversations with the participant. Medical assessments may be repeated for confirmation. At any time during Screening, if a potential participant is deemed to be ineligible and therefore qualify as a Screen Failure, study personnel will notify the potential participant that he/she is not eligible and no additional Screening assessments will be scheduled or conducted. All potential participants who begin

Screening will be tracked in the EDC study database and reasons for Screen Failure will be recorded.

At the discretion of the site PI or Study Physician, participants who screen fail may be eligible to be re-screened.

Screen Failures may request or be referred to an outside mental health clinician/ medical provider or to their health care provider, if needed.

Screen Failures are not considered evaluable.

10.2 Evaluable Participants

A participant is considered evaluable and eligible for the FAS analysis if he or she meets study eligibility criteria and is randomized to psilocybin or active placebo.

10.3 Early Termination from the Study

Participants can withdraw consent or terminate from the study at any time at his/her request without prejudice. Study personnel can withdraw a participant if, in their clinical judgment, it is in the best interest of the participant or if the participant cannot comply with elements of the protocol that are critical for safety or for the scientific integrity of the study. If study personnel withdraw a participant from the study, study personnel will explain the reason for withdrawing the participant. The reason for early termination will be recorded.

Randomized participants who prematurely terminate from the study will not be replaced.

10.4 Early Termination Post-Baseline and Pre-Randomization (Preparation Phase Termination)

It is possible that participants will discontinue study participation between the end of the Baseline assessments and up to randomization on the morning of dosing. This might occur for any of following reasons, including, but not limited to, a decision to withdraw, a decision on the part of site personnel to withdraw the participant prior to dosing, a change in mental or physical health status, or become ineligible to be randomized during the preparatory sessions. These participants will be considered Preparation Phase Terminations and will complete an End of Study visit within 14 days. Similar to Screen Failures, these participants will not be included in the FAS analysis population but will be included in the safety analysis set.

10.4.1 Early Terminations Post-Randomization and Pre-Dosing

Randomization is to take place immediately before dosing. If a participant is terminated or withdraws post-randomization but prior to dosing, the reason for termination will be documented and the participant terminated.

10.4.2 Early Termination Post Dosing

If termination from the study occurs between dosing and release from study site (approximately 8 hours post dose) the patient have to stay on-site until release, due to safety reasons. If a participant develops any condition post-dosing that, in the opinion of the PI (and upon consultation with the study Sponsor as needed), would negatively impact safety should

he/she remain in the study, the participant will be asked to complete the End of Study visit within 14 days. Efforts will be made to obtain information about AE outcomes, if deemed necessary by the PI, Medical Monitor and/or Sponsor.

For other post-dosing exclusion criteria not judged to impact participant safety, participants will be encouraged to remain in the study and continue with all follow up assessments, given that no additional exposure to the study medication is required following the dosing day. If a participant does not agree to remain in the study, or if a participant chooses to withdraw from the study for any other reason post-dosing, he/she will be asked to complete the End of Study visit assessments within 43 days of their dosing day, so that the maximal time between dosing and assessment is no longer than the time period for participants who complete the study.

10.4.3 Dropouts

If a participant withdraws consent, they will be terminated from the study without further follow up. These participants are defined as dropouts. If they withdraw consent prior to or during Baseline they are considered Screen Failures. If they withdraw consent post dosing then study records/data generated until the date consent is withdrawn will remain available for use by the Sponsor and PI.

10.4.4 Lost to Follow-up Post Dosing

A participant will be considered lost to follow-up if he or she repeatedly fails to return for scheduled visits post dosing and is unable to be contacted by the study site. The following actions must be taken if a participant fails to return to the clinic for a required study visit:

- The site must attempt to contact the participant and reschedule the missed visit as soon as possible and counsel the participant on the importance of maintaining the assigned visit schedule and ascertain whether or not the participant wishes to and/or should continue in the study.
- Before a participant is deemed lost to follow up, the PI or designee must make every effort to get in contact with the participant (where possible, 3 telephone calls and, if necessary, a certified letter to the participant's last known mailing address or local equivalent methods). These contact attempts should be documented in the participant's study file.
- Should the participant continue to be unreachable, he/she will be considered to have withdrawn from the study.

10.4.5 End of Study Definition

The end of the study is defined as the date of the last visit of the last participant in the study or last scheduled procedure shown in the Schedule of Assessments.

10.4.6 Premature Study Discontinuation

The Sponsor, MPA and IRB have the right to discontinue this study at any time. If the trial is prematurely terminated, the PI is to promptly inform the study participants and will assure appropriate referral and follow-up. If the study is prematurely discontinued, all procedures and requirements pertaining to retention and storage of documents will be observed. All other study materials will be returned to the Sponsor and will be treated in accordance with Swedish regulations.

11. INVESTIGATIONAL PRODUCTS

11.1 Description of Investigational Products

The investigational products (IP) to be used in this protocol are psilocybin and active placebo. [^{11}C]UCB-J is defined as a non-investigational medicinal product (non-IMP).

11.1.1 Psilocybin

Psilocybin is a tryptamine that produces its behavioral effects primarily by acting as post-synaptic agonist at serotonin 5-HT_{2A} and 5-HT_{2C} receptors (Sanders-Bush & Mayer, 2006). Refer to the psilocybin IB for a comprehensive review of the pharmacology, effects and proposed mechanisms of action of psilocybin.

11.1.2 Description of Active Placebo

Niacin, also known as nicotinic acid or vitamin B3, will be used as the active placebo in this protocol. Upon ingesting niacin, one commonly experiences a physiological reaction including warmth or flushing of the skin and mild dizziness.

11.2 Source

Usona Institute provides GMP psilocybin and niacin. Usona Institute has contracted the manufacture of cGMP psilocybin (active pharmaceutical ingredient), cGMP psilocybin capsules (25 mg) and cGMP niacin capsules (100 mg). Compendial grade (USP) niacin will be used in the preparation of the niacin active-control drug product.

11.2.1 Study Drugs Administered

Identical capsules will contain either 25 mg of psilocybin or 100 mg of niacin. Each capsule is provided in an HDPE bottle; bottles of study medication will be stored in the research pharmacy or other MPA-approved storage location of each study site. A capsule will be provided to a participant at the appropriate time during the dosing session. The participant will swallow the capsule with water.

Table 5: Psilocybin and Active Placebo Information

**Study Intervention Name:**	Psilocybin Capsule (active drug product)	Niacin Capsule (active placebo product)
**Dosage formulation:**	One active capsule contains 25 mg of psilocybin	One active placebo capsule contains 100 mg of niacin
**Capsule:**	Size 2 HPMC, white opaque	Size 2 HPMC, white opaque
**Unit dose strength:**	25 mg	100 mg
**Route of Administration:**	Oral (solid dose)	Oral (solid dose)
**Dosing instructions:**	One capsule administered with water	One capsule administered with water

**Packaging and Labeling:**	Study Intervention will be provided in an HDPE bottle. Each bottle will contain one capsule (psilocybin or niacin) and will be labeled as required per MPA requirement for blinded study.

11.3 Dosing

Participants in this study will receive one dose of either psilocybin or niacin during their dosing session. Participants randomized (or blindly-assigned) to psilocybin will receive a 25 mg of psilocybin taken with approximately 200 ml of water. Participants randomized to niacin will receive 100 mg of niacin taken with approximately 200 ml of water.

11.3.1 Drug Delivery, Storage, Handling, and Accountability

1. The PI or designee must confirm appropriate temperature conditions have been maintained during transit for all study intervention received and any discrepancies are reported and resolved before use of the study medication.
2. Only participants randomized in the study may receive study medication and only authorized site staff may supply or administer study medication. All study medication must be stored in a secure, environmentally controlled, and monitored (manual or automated) area in accordance with the labeled storage conditions with access limited to the PI and authorized site staff.
3. The PI (or designee) is responsible for study medication accountability, reconciliation, and record maintenance (receipt, reconciliation, and final disposition records).
4. Further guidance and information for the final disposition of unused study medications are provided in the Pharmacy Manual.

12. PET DATA ACQUISITION

MDD subjects will be examined with HRRT PET and [^{11}C]UCB-J at two occasions, within one week before (PET1) and within one to three weeks (day 15; -7/+7 days) after (PET2) completion of the psilocybin/placebo treatment. At each PET measurement the subject is placed recumbent with the head in the PET system. A head fixation system with an individual plaster helmet is used [97]. [^{11}C]UCB-J is synthesized as previously described[17]. A sterile physiological phosphate buffer (pH 7.4) solution containing 400 MBq (±10%, for a subject of 70 kg) [^{11}C] UCB-J is diluted with saline to the volume of 10 ml and then injected as a bolus during 10 seconds into a cannula inserted into an antecubital vein. The cannula is then immediately flushed with 10 ml saline. Arterial cannulation in either a. radialis is performed for sampling throughout the experiment (total volume circa 70ml). The effective dose for each PET experiment will be approximately 2.4 mSv, based on the assumption of 0.006 mSv/MBq 11C-labelled radioligands[98]. The PET system used is ECAT HRRT (High Resolution Research Tomograph, Siemens Molecular Imaging). A 6-min transmission scan using a rotating 137Cs source is first acquired for attenuation correction. Emission data is acquired in list mode for a period of 93 min. Dynamic images are reconstructed in a series of 31 time frames (4 × 15 s, 4 × 30 s, 6 × 60 s, 6 × 180 s, 11 × 360 s) using three-dimensional ordinary Poisson ordered subset expectation maximization (OP-3D-OSEM), including modeling of the system's point spread function (PSF). This procedure has previously been shown to correspond to a resolution of approximately 2 mm[99]. To enable quantification of specific

binding, arterial blood will be sampled from a cannula in a radialis dx or sin throughout the measurement. A trained anesthesiologist will position the cannula within two hours before each examination. Altogether some 80 ml blood will be sampled during each PET experiment.

MADRS, CGI-S and CGI-I (PET2) rating will be performed at day of PET1 and PET2.

12.1 [11C]UCB-J

[11C]UCB-J, (4*R*)-1-[(3-[11C]methyl-4-pyridyl)methyl]-4-(3,4,5-trifluorophenyl)pyrrolidin-2-one, is a radioligand for quantification of Synaptic Vesicle protein 2A, (SV2A). In radioligand binding studies at 37°C, UCB-J displayed high SV2A binding affinity: pKi of 8.15 (7 nM) for humans and 7.6 (25 nM) for rats. UCB-J exhibited a greater than 10-fold and greater than 100-fold selectivity for SV2A over SV2C and SV2B protein, respectively. Moreover, UCB-J at 10 mM lacked any significant interaction (>50% inhibition) with a wide variety of receptors, ion channels, enzymes, and transporters[36]. Refer to the [11C]UCB-J non-IMPD for a comprehensive review of the compound.

[11C]UCB-J is synthesized at Karolinska PET center in accordance with the non-IMPD.

12.2 PET Image analysis

PET List mode data is reconstructed using the ordinary Poisson 3D ordered subset expectation maximization algorithm, with 10 iterations and 16 subsets, including modelling of the system resolution. PET images are corrected for head motion with a frame-to-frame-realignment algorithm as described by Schain et al.[100] .

T1-weighted (T1-w) MRI-images are coregistered to PET-images and segmented into grey matter, white matter and cerebrospinal fluid in SPM5 (Statistical Parametric Mapping, Wellcome Trust Centre for Neuroimaging, U.K.). Regions of interest, ROIs, will be chosen based on relevance for the pathophysiology of depression according to the literature [101]. Cortical reconstruction and volumetric segmentation was performed with the Freesurfer image analysis suite, which is documented and freely available for download online (http://surfer.nmr.mgh.harvard.edu/). Based on the variation in radioactivity in arterial blood and in the different ROIs over the time period of data aquisition, the Volume of distribution, Vt, for [11C]UCB-J will be calculated for each ROI. The parameter of comparison will be Vt_{ratio}: $Vt_{target\ ROI}/Vt_{reference\ ROI}$.

13. PERIPHERAL BIOMARKERS SAMPLED AT TIME OF PET1 AND PET2

Blood will be analyzed for L-acetylcarnitine[102], proBDNF, BDNF, VEGF, the Ca^{2+} binding protein S100B[103] the translocator protein p11[18] and related potential response markers of psilocybin treatment in MDD.

Briefly, 30 ml whole blood will be sampled in EDTA tubes. 10 ml will be used for preparation of serum and blood cell pellets stored for possible later DNA extraction. 20 ml will be diluted 1 to 1 with PBS and peripheral blood mononuclear cells (PBMCs) will be

isolated by density centrifugation. Cells will be stored at -80$_{o}$C in 90% FCS and 10% DMSO until use. P11 levels will be measured in monocyte, and T cell subsets and NK cells by intracellular staining using a monoclonal p11 antibody. Stained cells will be analyzed by multicolor flow cytometry carried out with a Beckman Coulter Gallios. Data will be analysed with Flow Jo using an appropriate color compensation matrix to correct for spectral overlap and autofluorescence and an isotype control antibody will be used to confirm antibody specificity for p11. Samples will be kept for ten years.

14. SAMPLING AND ANALYSIS OF CEREBROSPINAL FLUID

Cerebrospinal fluid (CSF) (5 ml) will be sampled at two occasions, between screening visit and Baseline (CSF1), and at PET2 (CSF2). Samples will be analysed for monoamines, monoamine metabolites[104], cytokines, kynurenic acid[25] and related potential response markers to psilocybin treatment of MDD including genetic markers. Samples will be kept for ten years to enable additional analyses of the same research questions with future, improved methods.

15. MAGNETIC RESONANCE IMAGING (MRI) EXPERIMENTAL PROCEDURE AND DATA AQUISITION

Collection of MRI data will occur on two occasions: 1) as a part of post preparatory assessment (7-35 days prior to dose) and 2) day 1, post dose, i.e the day after the treatment has been given. Brain imaging sessions will be performed at the Solna MRI center, Department of Clinical Neuroscience, Karolinska Institutet. on a 3-Tesla GE scanner. The protocol includes acquisition of structural 3D T1 MPRAGE, three functional MRI (fMRI) tasks, Diffusion Tensor Imaging (DTI), resting state fMRI (rsfMRI) with presentation of abstract figures – all are standardized methods at the MRI center. The fMRI tasks will include: reversal aversive learning (RALT), perspective shifting (P-SHIFT), and emotion detection under perceptual uncertaintly (EDPU). During the first session no task-based fMRI will be performed, otherwise the sessions are the same. The planned scanning session will take 1 hour (session 1) and 1.5 hours (session 2) to complete including preparations and instructions. Research coordinators and assistants possessing an MRI driver license are supervising these assessments. The MRI scanning sessions can be stopped by the subject at any time by activating a squeeze-ball provided to the subjects in order to inform the researchers that the procedure needs to be terminated.

In case of accidental finding requiring medical attention (the scans will be evaluated by a neuroradiologist) the subject will be contacted by the responsible study MD and further steps will be discussed (e.g. referral to a specialist).

15.1 fMRI tasks

Task	Description

RAVLT
(Reversal Aversive Learning)

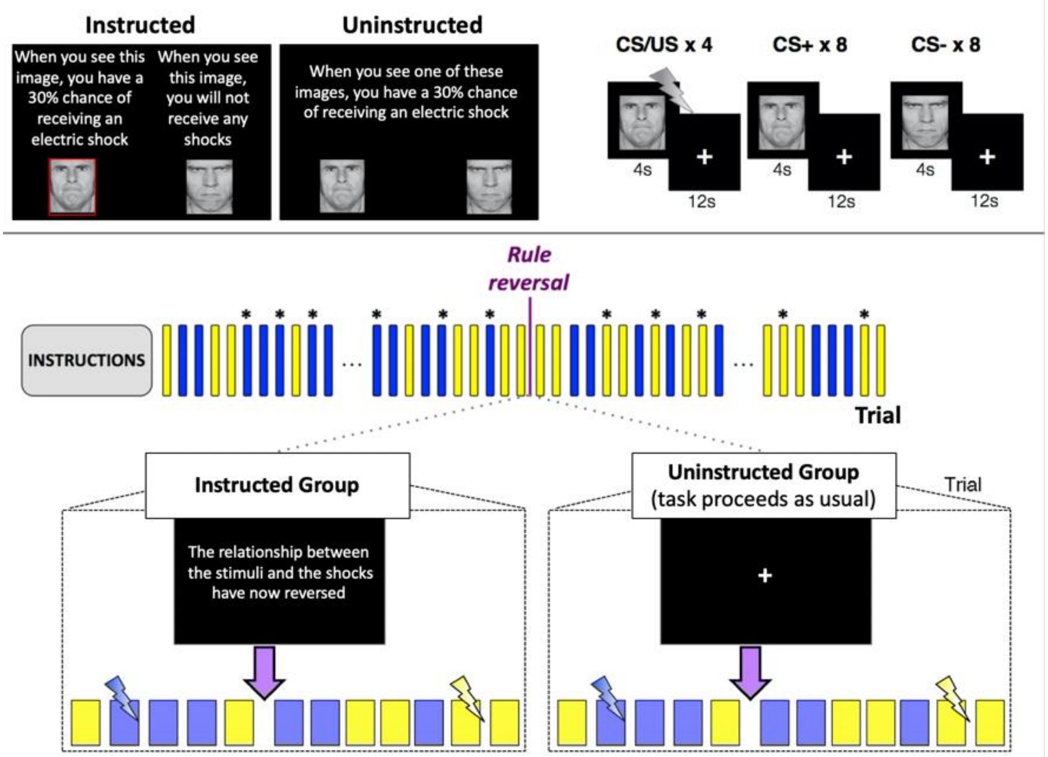

The task incorporates a reversal fear learning procedure as described in (Atlas, Doll, Li, Daw, & Phelps, 2016). Two images of male faces with angry facial expressions will be used as conditioned stimuli in order to potentiate the conditioning effect (Pischek-Simpson, Boschen, Neumann, & Waters, 2009) . A mild electric shock will be used as an unconditioned stimulus (aversive but tolerable electric stimulation) coupled with one of the faces with a 30% chance. Each block will contain 4 trials of the conditioned stimulus coupled with electric shock (CS/US), 8 trials of the same stimulus, but without the shock (CS+) and, finally 8 trials of stimulus that is not associated with electric shocks (CS-). Contingencies will reverse 3 times through the task and the subjects will be explicitly informed about contingencies prior to the task and upon the rule reversal. On each trial, subjects' fear reactions will be measured with a galvanic skin response (GSR) and pupillometry and fMRI imaging. Subjective likability ratings will be collected before and after the procedure.

P-SHIFT
(Perspective-Shifting)

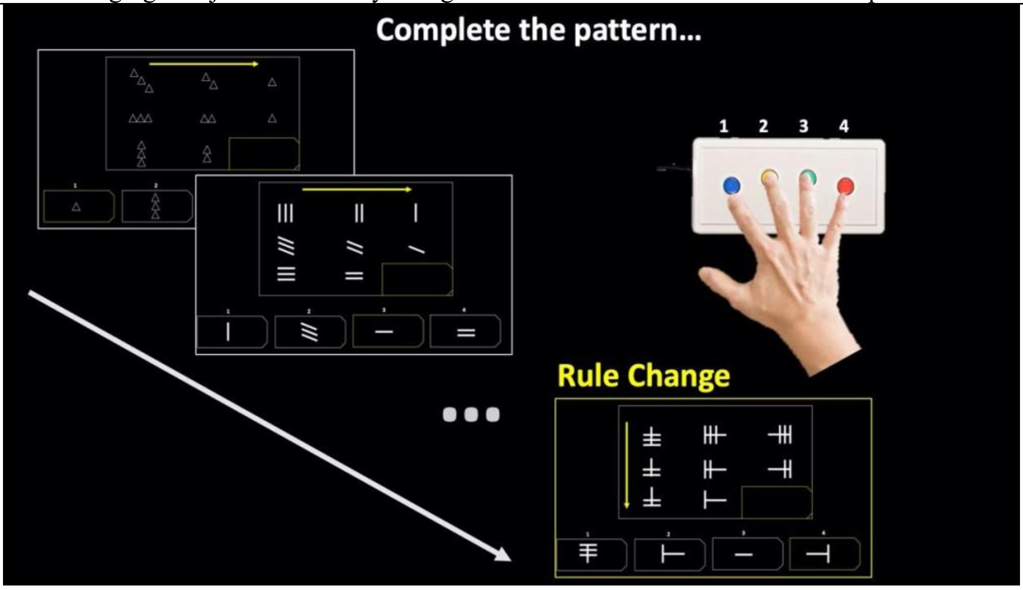

The subjects are presented with a series of puzzles similar to the ones from Raven's progressive matrices. The task is to choose the answer that completes the pattern. Two types of blocks are presented: simple and difficult analytical. The task is self-paced with fixed block duration of 2 min. The subjects are not aware of the rules, which they have to figure out themselves. The rules change several times within blocks.

**EDPU** (Emotion detection under perceptual uncertaintly)	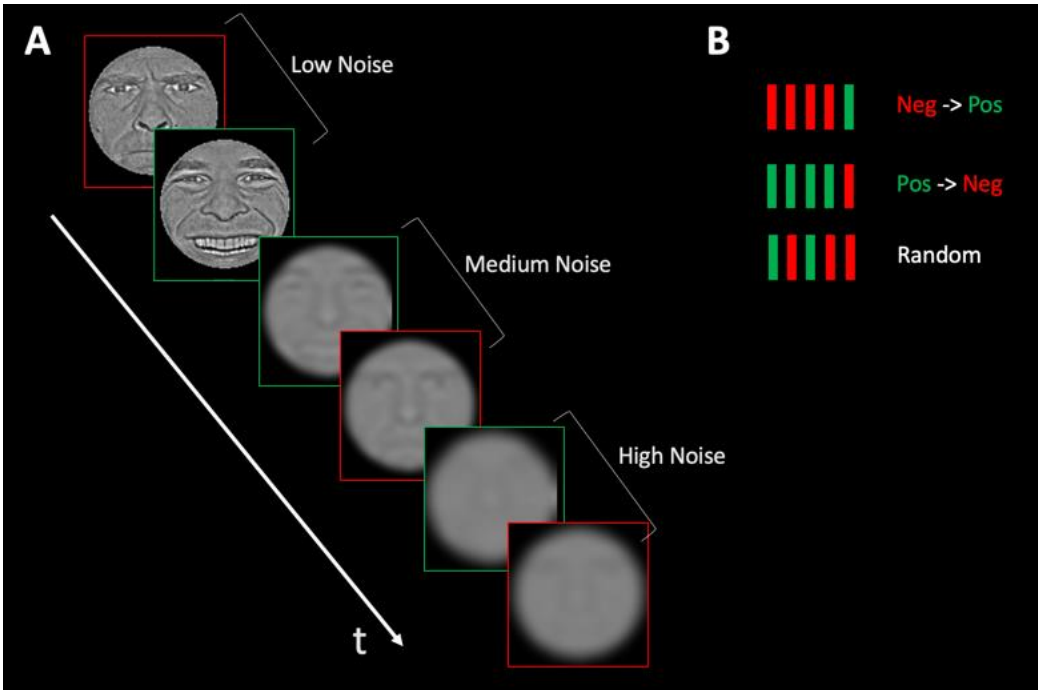 The task incorporates binary choice paradigm, in which a subject has to make a decision whether he/she sees a smiling or angry face. Experimental manipulations will address effects of perceptual uncertainty and emotional valence of preceding sequences.

15.2 MRI safety

All subjects will undergo an MRI procedure described above. Additional exclusion criteria for this part are metal body implants and pacemakers, claustrophobia, weight over 120 kg. In order to prevent acoustic trauma, hearing protection is used during all MRI sessions with estimated incoming noise intensity at 90 dB, which is considered to be safe.
In addition, the subjects will be provided with a squeeze-ball (an emergency response), using which they can request researchers to terminate the scanning session.
It is known that the sound produced by an MRI machine may be disturbing for some individuals. Therefore, all participants will be instructed that they can stop their MR scanning sessions anytime on demand.

16. STATISTICAL ANALYSIS

16.1 Power Analysis and Sample Size Determination

The difference between psilocybin and placebo in change from baseline to Day 8 in MADRS total score (primary endpoint) will be analyzed for the Full Analysis Set using Analysis of Covariance including treatment as main effect and baseline as covariate.

Based on prior controlled studies of psilocybin in cancer- related depression and anxiety[1], a decrease in depression symptoms from baseline to one-week post-dose of at least 30% is expected in the placebo arm. In an open-label study of psilocybin[2] the change in MADRS total score from baseline to 1 week was -23.3. Several clinical studies in the literature report a standard deviation in change in MADRS total score around 10.

A sample size of 15 in each group will have 80% power to detect a difference in means of 11 (the difference between a psilocybin mean, μ_1, of -23 and a placebo mean, μ_2, of -12) assuming that the common standard deviation is 10 using a two group t-test with a 5% two-sided significance level.

Considering the short duration between treatment and primary endpoint measurement day 8 the risk of withdrawal is considered low. The total sample size is set to 30 patients completing day 15. In case of withdrawals before day 15 additional patients may be randomized to ensure 30 patients included in the primary and secondary analyses. Estimating a maximum of 20% withdrawals, a maximum of 6 additional subjects can be included.

16.2 Populations for Analyses

Efficacy analyses of primary, secondary and exploratory outcomes will be conducted using the FAS population and the Safety set will be used in all safety analyses.

Full Analysis Set (FAS)
All participants randomized to receive a study intervention (psilocybin vs. active placebo) will be included in FAS.

Per Protocol Set (PP)
All participants randomized to receive a study intervention (psilocybin vs. active placebo) with no relevant protocol deviations will be included in PP. Relevant protocol deviations will be defined in the Statistical Analysis Plan and finalized before breaking the blind. The Per Protocol set will be used in sensitivity analyses for the primary and secondary objectives.

Safety set
All participants who have received study treatment will comprise the overall safety set.

16.3 Statistical Hypothesis

The purpose of this study is to compare psilocybin with placebo in treating depression in the population of interest. The null-hypothesis to be tested is:

H_0: Psilocybin is equal to placebo with respect to the treatment of depression

against

H_1: Psilocybin is not equal to placebo with respect to the treatment of depression

16.4 Statistical Analyses

Descriptive statistics (proportions, means, standard deviation, quartiles) will be used to summarize all demographic data as well as efficacy and safety endpoints. Distributional features

of all measurements will be evaluated, and data transformation (e.g., logarithm, rank transform) will be performed when applicable.

16.4.1 Efficacy Analyses

The difference between psilocybin and placebo in change from baseline to Day 8 in MADRS total score (primary endpoint) will be analyzed for the Full Analysis Set using Analysis of Covariance including treatment as main effect and baseline as covariate. The same method will be applied for all other single measure endpoints. Treatment differences of multiple measurement endpoints will be analyzed by the mixed model repeated measurements analysis (MMRM) method.

Response and remission rate will be analyzed using logistic regression with the baseline measure as a covariate. The adjusted percentage with 95% CI, Odds Ratio with 95% CI and nominal p-values for the treatment comparison will be presented.

Time to initiation of antidepressant will be defined as the time (in days) from day 43 to the date when any antidepressant has been prescribed. Kaplan-Meier plots of time to initiation of antidepressant will be displayed by treatment groups. Time to initiation of antidepressant will be censored at study discontinuation or completion.

If the assumption of normality does not hold for a particular endpoint on the original scale of measurement, nonparametric methods (e.g. Mann Whitney U test, Fisher's exact) or common transformations (e.g., ln, sqrt) of the original data may be used.

P-values less than 0.05 will be considered statistically significant. As default all tests will be two-sided and two-sided 95% confidence intervals will be computed for the treatment difference. However, any hypothesis that includes a direction of change, eg *increased* [11C]UCB-J binding after dosing, will be tested using one-sided test using a significance level of 0.025.

16.4.2 Multiple comparisons

Considering the exploratory nature of the study endpoints no adjustments for multiplicity will be made. However, due to the large number of exploratory endpoints and many timepoints for evaluations, results for these endpoints will be interpreted with caution.

16.4.3 Handling of missing data

Missing values will in general not be imputed in the analyses. Participants who withdraw from the study for any reason after receiving a study intervention but prior to the Day 8 assessment will be asked to complete an exit assessment of the primary study endpoint (change in MADRS total score from Baseline to Day 8 post-dose). It is anticipated that the temporal proximity of the primary endpoint to the study intervention, combined with efforts to obtain an exit assessment for participants who drop prior to the primary endpoint, will ensure that missing data (either missing at random or missing not at random) will be negligible.

Moreover, obtaining a MADRS total score at several occasions post dosing (Table 1) will provide additional data points prior to and after the Day 8 primary endpoint. Participants who receive a study intervention but miss the Day 8 post-dose assessment for any reason other than study discontinuation will be invited to continue in the study and all other

subsequent assessment points will be collected to allow the participant's data to be included in analyses of secondary endpoints, as well as the safety data.

16.4.4 Interim analyses

The primary analysis of this study will be conducted when all patients have completed the Day 42 assessment. The patients will continue in the study until Day 365 when the follow-up analysis will be done.

16.4.5 Subgroup Analyses

No subgroup analyses will be conducted.

16.4.6 Safety Analyses

All recorded AEs will be coded using MedDRA and presented by SOC and preferred term with number of subjects, percentages and number of events overall and by study period. The worst severity will be used for each event. TEAEs, solicited AEs, SAEs and AEs related to treatment will be summarized similarly. AEs leading to discontinuation from the study will be listed and tabulated.

All concomitant medications will be listed. Safety data collected for the period from Screening through the Baseline assessment and during the Preparation Phase will be presented separately from post-dose safety data (treatment emergent).

All solicited AEs observed in randomized participants who received psilocybin or niacin placebo will be reported.

17. SAFETY MANAGEMENT

Below are the risks associated with participation in this study as well as the risk mitigation strategy associated with each risk. Safety measures will be applied, as described below, to minimize risks associated with participation in this study. This study proposes a combination of careful screening, preparation, supervision, and follow-up designed to minimize any risks.

Presented below are several domains that relate to adverse effects of psilocybin administration, which are further detailed in the IB. Following the section on psilocybin, the risks associated with the non-drug aspects of the study are described. Participation in this study may include risks that are currently unknown.

17.1 Risks Associated with Psilocybin

17.1.1 Physiological and Psychological Adverse Effects

The clinical safety of psilocybin has been extensively studied, both as a single agent and as adjunctive treatment in adult populations. Psilocybin is administered orally, and has been studied in open-label, and double-blind, controlled trials. Dosing regimens have ranged from 0.014 mg/kg to 0.6 mg/kg, administered as either a single dose, or multiple doses weeks apart.

Adverse event data from previous clinical trials has been used to evaluate the physiological profile of psilocybin. The IB for psilocybin summarizes the adverse event data collected from controlled clinical trials utilizing psilocybin in conjunction with cognitive enhancement therapy across multiple subpopulations.

The most likely potential acute adverse effects of psilocybin has been shown to be anxiety, as well as panic, delusion, and cognitive impairments, particularly at higher doses (> 25 mg oral psilocybin) during the period of acute drug action. Such transient episodes of fear or anxiety respond well to verbal reassurance and have not required pharmacological intervention. In previous clinical experience, acute psychological events were resolved by the end of the dosing day.

Overall, the most commonly reported physiological adverse events associated with psilocybin are:

- cardiovascular changes (including increased blood pressure and heart rate)
- nausea
- headache

These adverse events were generally classified as mild to moderate and were found to be transient in nature. No psilocybin-related serious adverse events were reported.

17.1.2 Visual Perceptual Effects

Some people who have used serotonergic hallucinogens, such as psilocybin, experience persistent, distressing alterations in mostly visual perception that last from weeks to years after use[105]. This condition is now diagnosed as hallucinogen persistent perception disorder (HPPD).

To date, however, no cases of HPPD have occurred in volunteers given psilocybin in contemporary research studies[106]. The risk of HPPD occurring after psilocybin administration can be reduced by screening participants for potential risk factors such as substance dependence and by excluding people reporting HPPD or other significant adverse events after prior use of hallucinogens.

Visual perceptual effects will be assessed as a solicited AE during Periods 3 and 4.

17.1.3 Drug Interactions

See Appendix A for a list of prohibited medications. Additional information is provided in the IB.

17.1.4 Risk of Worsening MDD

An exacerbation of depressive symptoms including increased suicidal ideation could occur prior to or during the study. Participants in the study will be foregoing established treatments for depression while they are enrolled in the study. This risk of having a depressive exacerbation may be greater for participants in the niacin placebo group, as they will undergo a longer period of time without receiving a known active treatment. Although placebo interventions often have significant therapeutic benefits in patients with MDD, participants who receive niacin placebo may be at increased risk for worsening of depressive symptoms or development of active suicidal ideation with plan during the course of the study.

Study clinicians at each site will assume the role of mental health provider when a participant enrolls in the study through the final primary outcome time-point at post-dose Day 42. Monitoring for suicidality will be ongoing. Study team members will inform a study clinician of any substantial worsening of depression or suicidality, or of any other concerns regarding a participant's health. Study clinicians will then assess the health and safety of this participant. If a study clinician decides that an antidepressant medication is warranted, or if the participant desires conventional treatment during or after the study, the study clinician will develop an appropriate treatment plan including referring the participant to an outpatient provider or to the clinician treating the participant prior to study entry.

17.2 Risks Associated with Collection of Potentially Sensitive Information

Sensitive information may be revealed to study personnel during the screening process and/or during the course of the study. Identification of disease may occur during the screening process and may impact future insurability of the participant. In addition, some diagnoses can create stigmatization or self-stigmatization for the participant. Both the PI and participants may be at risk for a violation of privacy and/or loss of confidentiality. This risk will increase as the study progresses as ongoing health information will be collected.

Best practices will be followed at all stages of the study to ensure maximum protection in the collection, storage and usage of potentially sensitive information. All collected data will be stored on a secure electronic database coded by a study ID number and stored separately from any documents with personally identifiable information and/or a key that would link such information to study data. All research staff will complete GCP training to promote the proper conduct of scientific research in humans

17.3 Risks Associated with Recordings of Sessions

To evaluate the Facilitators' adherence to the study manual, and the role of Facilitator's and participant's in-session behaviors for treatment outcome, the preparation session and all three integration sessions will be recorded using a digital voice recorder. The dosing session will be documented using a digital film camera. There is a risk that these recordings of sessions will make participants feel monitored, controlled and/or in any other way uncomfortable. However, recording of treatment sessions is a standard procedure in graduate and advanced therapist training, and as long as clients understand the purpose of the confidential recordings, they are often happy to consent[107,108]. Clients who agree to being recorded are also generally happy to do so, and the recording instruments are quickly forgotten[107,108]. In this study, upon requests from the participant to further examine the details and small nuances of the dosing day experience, (parts of) the filmed dose session will be used during the integration sessions.

Study participants will be informed of the recordings of sessions (i.e., why the sessions are being recorded and what they are used for) at the In-Person Screening visit, before signing the informed consent. The recordings will be kept securely in a locked cabinet at Mottagningen för hjärnstimulering. Collected data will then be stored on a secure electronic database coded by a study ID number; separately from any documents with personally identifiable information and the key for linking such information to study data.

17.4 Risks Associated with Psychiatric Questionnaires

The study psychiatric assessments may uncover strong and potentially disturbing feelings about the participant's past or present emotional state. While it is expected the risk for SAEs resulting from psychiatric assessments is very low, the likelihood of these risks occurring will be minimized by procedures described below.

Care will be taken to avoid bringing about undue psychological distress during the psychiatric interviews. This will be accomplished by using trained central and on-site raters and by collecting the "minimum necessary" information required for study purposes. In the event that a participant becomes unduly distressed, a study clinician will be immediately contacted, and an appropriate clinical intervention plan will be developed.

17.5 Risks Associated with venepuncture and arterial cannulation

The risks of drawing blood include discomfort, bruising, infection, bleeding and fainting. The amount of blood drawn during the study (approximately 250 mls, at three different occasions, 2-3 weeks apart) will not have any adverse physiological effects, nor will it lead to any long-term distress. Risks for blood borne pathogens from accidental needle stick and during sample processing exist. Not uncommonly, a bruise may form at the puncture site. Arterial cannulation will be performed in local anesthesia after Allen's test in order to verify palmar collateral circulation.

To reduce risk of infection and bleeding standard sterile procedures for drawing blood will be used by certified personnel with extensive phlebotomy experience. To reduce the risk of fainting all participants will be asked about tendency to faint prior to venipuncture and will be placed in a recumbent position if they answer in the affirmative.

17.6 Risks Associated with lumbar puncture

Lumbar puncture (LP) for CSF collection will be performed at two occasions some 2-4 weeks apart (all together ca 10 mL of CSF) and after MR-imaging of the head, which will enable identifying signs of increased intracranial pressure. LP will be performed in the L4-L5 or adjacent lumbar intra columnar spaces, with the participant either lying down on the side, or sitting, with maximal kyphosis in the lumbar segment, and in local anaesthesia. Still there is a risk of post LP headache. The first line of treatment for post-LP headache is recumbence, hydration and analgesics as needed. However, if post-LP headache persists after a day of such treatment, administration of an epidural blood patch will be considered.

17.7 Risks Associated with Positron Emission Tomography (PET)

A tracer dose of [$_{11}$C]UCB-J is to be administered iv at two occasions. This is associated with a risk of infection and/or thrombophlebitis at the place of intravenous administration. The exposure to radiation is theoretically associated with a risk of DNA-damage that, in the case of faulty DNA-reparation, may result in non-functional protein synthesis. This is considered a potential mechanism for development of dysplastic events.

17.8 Contacting Emergency Services

Generally, if at any time during the study, including during your dosing session, study staff feel a participant is in danger of hurting him/herself or someone else then the study physician, PI and/or Lead Facilitator will be contacted immediately for further evaluation and follow up. In certain circumstances 112 may be called or the participant may be taken to the emergency room, including the following:

- If at any time during the study physician, PI and/or Lead Facilitator feels your situation is an emergency that requires immediate attention they will call 112 or the participant will be taken to the emergency room.
- If the participant becomes violent or aggressive during the dosing session and he/she does not respond to medications or calming by your session Facilitators, then the study physician, PI and/or Lead Facilitator may call 112, or the participant may be escorted to the emergency room.
- If the participant insists on leaving the research site before the dosing session is complete (they must remain on site for a minimum of 7 hours) and the study physician, PI and/or Lead Facilitator feels he/she is a threat to him/herself or others then they will call 112.
- If the study participant insists on leaving the research site before the dosing session is complete and the study physician, PI and/or Lead Facilitator do not feel he/she a threat to him/herself or others then they will call the support person previously identified to take the participant home.

If the study physician, PI and/or Lead Facilitator feels the participant may have a serious, but not urgent, psychiatric condition then he/she will be referred to his/her previously identified mental health provider, if one was identified, or to mental health services in the community.

17.9 Abuse Liability

Like other psychoactive drugs, psilocybin is sometimes used in a manner that jeopardizes the safety or well-being of the individual or others (e.g., driving while impaired; a pattern of use that interferes with work, school, or relationships). Under such circumstances, psilocybin would be said to be *abused*. However, psychedelic medicines such as psilocybin are not typically considered drugs of *dependence* in that they do not engender compulsive drug seeking behavior[109], consistent with the observation that they are not reliably self-administered in nonhuman animals[110–112]. Further, they are not associated with a known withdrawal syndrome[113]. Therefore, there is little risk that exposing human volunteers to psilocybin will leave participants physically or psychologically dependent on the compounds. In previous[47,48] and ongoing studies with psilocybin, exposing individuals with either no history of hallucinogen use or a history of minimal use (e.g., less than 10 times total and not within the last 5 years) in the context of a supervised and controlled research setting has not resulted in reported instances of subsequent illicit hallucinogen abuse. In the meta-analysis conducted by Studerus and colleagues[106], the large majority of participants in psilocybin studies (approximately 90%) reported "no change" in their psilocybin use following their laboratory sessions, as well as "no change" in their overall drug consumption habits (e.g., use of alcohol, nicotine, cannabis, MDMA). Those who did report changes often described decreased consumption (see Table 5 in Studerus et al., 2011). Specifically, in terms of psilocybin use, more participants reported using it *less* often after their laboratory sessions (5.6% of all participants) than more often (3.3% of all participants).

17.9.1 Abuse Monitoring

Based on current information, it does not appear that psilocybin demonstrates signals associated with known abuse liability patterns when administered in a therapeutic setting under continuous observation in a single-dose session, for which further evidence will be collected in this phase 2 study. Any abuse potential is further limited since the drug is not supplied to the participant to take home and is administered under careful clinical supervision in a restrictive setting. Participants will never have the study drug in their possession or have access to it outside the closely supervised clinical setting, which removes the possibility of drug diversion (e.g., missing medication, loss of drug) or unrecognized non-compliance with medication ingestion. While there is still a possibility of site drug diversion, this risk is mitigated through Drug Accountability process, documentation and monitoring.

The following measures will be employed to monitor the abuse liability of psilocybin:

- Cases of noncompliance, protocol violations, participants lost to follow-up, and any other reasons why participants dropped out of the study will be assessed for signals of abuse
- Qualitative urine drug test data will be collected at Screening, Baseline and all post-dose assessments. Any positive findings that cannot be attributed to pre-approved concomitant medications or diet will be reviewed by the site medical clinician for evaluation and management.
- Prior lifetime illicit and non-prescription drug use will be collected at Screening via the MINI interview and reassessed by self-report from Baseline to Randomization. Self-reported behaviors of alcohol and illicit and non-prescribed drug use will be collected via the AUDIT and DUDIT at web screening. Additionally, urine drug screens, as described above, will be used to identify drug use during the study period. Behaviors will be compared before and after intervention in exploratory analyses. These measures will be used to provide objective data to assess the impact of single-dose psilocybin on addictive behaviors, not just in relation to non-study psilocybin use but also in relation to other addictive substances/behaviors.
- Assessment of solicited AEs to identify drug and alcohol abuse/misuse as defined by the MINI, identification of substance and alcohol use disorders as defined by the MINI, and overdose with suicidal intent.

Evidence of any substance or alcohol use will be captured through the above measures and will not be reported as an AE. Should any substance or alcohol use be considered a SAE, it will be reported on a SAE form in addition to being captured through the assessments described above.

18. ADVERSE EVENTS

18.1 Definitions

18.1.1 Adverse Event

An AE is any untoward medical occurrence in a research participant, whether or not considered drug related which occurs during the conduct of a clinical trial. Any change in

clinical status, ECGs, routine labs, x-rays, physical examinations, etc., that is considered clinically significant by the PI is considered an AE.

Blood pressure (BP) and heart rate (HR) must meet the following criteria to be considered an AE for the purposes of this study:

- Systolic blood pressure (SBP) >140 mmHg or diastolic blood pressure (DBP) >90 mmHg on three separate readings
- Clinically significant decrease in BP
- Heart rate (HR) >100 beats per minute (BPM)
- Clinically significant decrease in HR

The AE terms listed below are MedDRA Preferred Terms to be used in classifying abuse liability AEs when deemed appropriate by the PI (or designee):

- Euphoria-related terms: euphoric mood, elevated mood, feeling abnormal, feeling drunk, feeling of relaxation, dizziness, thinking abnormal, hallucination, inappropriate affect
- Terms of impaired attention, cognition, and mood: somnolence, mood disorders, and disturbances
- Dissociative/psychotic terms: pychosis, aggression, confusion, and disorientation

18.1.2 Treatment Emergent Adverse Events

Treatment emergent adverse events are undesirable events not present prior to medical treatment, or an already present event that worsens either in intensity or frequency following the treatment. Events with an onset after study drug administration will be considered TEAEs.

18.1.3 Solicited Adverse Events

The following solicited AEs will be collected:

Visual perceptual effects (Periods 3-4) will be solicited by asking the following questions: 1) *Since your dosing session have you experienced any uncontrolled or disturbing return of study drug effects?*, and 2) *Since your dosing session have you experienced any visual distortions (e.g. geometric hallucinations, false perceptions of movement in the peripheral visual fields, flashes of color, intensified color, trails for images of moving objects, positive after-images, halos around objects)*? If a participant reports "Yes" to any of the above questions a study psychologist or psychiatrist will follow up for further assessment/diagnosis.

Additional solicited AEs include:

- Active suicidal ideation identified through MADRS and verified by clinical assessment.
- Headache; and
- Nausea and
- Elevated blood pressure (BP) as defined by systolic blood pressure (SBP) >140 mmHg or diastolic blood pressure (DBP) >90 mmHg on three separate readings and requiring medication; and

- Elevated heart rate (HR) as defined as >100 beats per minute (BPM) and requiring medication; and
- Drug and alcohol abuse/misuse as defined by the MINI; substance and alcohol use disorders as defined by the MINI; or drug overdose with suicidal intent.

Monitoring for suicidality will be ongoing throughout the duration of the study using MADRS. If at any time during an evaluation or follow up there is concern a participant is suicidal study staff will follow standard of care procedures for managing suicidality, eg as described in https://psykiatristod.se/regionala-vardprogram/depression.

To assess for headache after psilocybin administration, participants will be asked if they have or experienced a headache during Period 3, and these will be classified as a migraine, tension headache, or other headache. On the dosing day, participants reporting headache will be encouraged to take non-steroidal anti-inflammatory agents or acetaminophen at home, if needed.

Visual perceptual effects, suicidal ideation verified by clinical assessment, headache, nausea, and overdose with suicidal intent will be reported through normal AE/SAE mechanisms.

18.1.4 Serious Adverse Events

An SAE as determined by the PI or the Sponsor is any event that results in any of the following outcomes:

1. Death
2. Life-threatening AE (Life-threatening means that the study participant was, in the opinion of the PI or Sponsor, at immediate risk of death from the reaction as it occurred.)
3. Inpatient hospitalization or prolongation of existing hospitalization
4. Persistent or significant incapacity or substantial disruption of the ability to conduct normal life functions
5. Congenital abnormality or birth defect
6. Important medical event that may not result in one of the above outcomes but may jeopardize the health of the study participant or require medical or surgical intervention to prevent one of the outcomes listed in the above definition of serious event.

18.1.5 Definition of Terms

Life threatening: A life threatening AE is defined as an event in which the participant is at risk of death at the time of the event. It does not refer to an event that hypothetically might have caused death if it was more severe.

Disability: A disabling AE is defined as a substantial disruption of a person's ability to conduct normal life functions.

Important Medical Event: Examples of such events include invasive or malignant cancers, intensive treatment in an emergency room or at home for allergic bronchospasm, blood dyscrasias or convulsions that do not result in hospitalization, or development of drug dependency or drug abuse.

18.2 Guidelines for Assessing Intensity of an Adverse Event

The PI should use the following definitions when assessing Intensity of an AE:

- MILD: Participant is aware of symptoms or has minor findings, but tolerates them well and no or minimal intervention required
- MODERATE: Participant experiences enough symptoms or findings to require intervention
- SEVERE: Participant experiences symptoms or findings that require significant intervention

18.3 Guidelines for Determining Causality of an Adverse Event

The PI and Medical Monitor will use the following question when assessing causality of an AE due to the study drug or as a result of study participation: Is there a reasonable possibility that the study drug or procedure caused the event?

A reasonable possibility implies there is evidence that the specific event was caused by the study drug or as a result of participation in the study. An affirmative answer designates the event as a suspected adverse reaction, and the AE is therefore considered "related." If the answer is no, then the AE is considered "unrelated."

18.4 Actions to be Undertaken

The PI is responsible for the appropriate medical management of all AEs and for the personal safety and well-being of participants. In case of an AE, the PI will initiate appropriate treatment according to his/her medical judgment and will decide whether to withdraw the participant from the study.

18.5 AE Collection Period and Follow up

All AEs and SAEs will be monitored continuously during the study from the time of signing the ICF until the End of Study. SAEs will be followed until resolution, including post Day 365/End of Study if warranted.

18.6 Regulatory Reporting

All SAEs will be reported by PI or designee, whether or not considered causally related to psilocybin, placebo or to the radioligand/ study procedure(s). SAEs will be collected in a list, which will be sent to the Swedish Medical Products Agency (MPA) and the IRB once a year until the study is on-going. In the case of a SAE, an assessment of the security of the subjects participating in the study will also be included. For fatal or life-threatening AEs where important or relevant information is missing, active follow-up will be undertaken immediately by the investigator by interview and/or physical examination and follow-up as deemed necessary by the investigator. In order to enable specific treatment of drug related AEs unblinding will be possible via the investigator (24 hrs phone accessability). Unblinding will be only performed if it is necessary for the investigator to have this knowledge when making a decision on how to treat an AE. In case the knowledge will not affect treatment choice the blind will not be opened.

The following information is mandatory for the initial report:

- Subject study ID
- Study treatment
- Start date (time, if relevant) of the study treatment
- Brief description of the event (diagnosis)
- Start date (time, if relevant) of the event
- Seriousness criteria
- Causality assessment

The investigator has to inform sponsor of all SAEs within 24 hours of the investigator's awareness using a Safety-approved report form (e.g., specified SAE form or local regulatory form) and perform follow-up activities. These will include interview and physical examinations as deemed relevant and continue as long as is deemed clinically relevant by the investigator.

SLSO is responsible for the recording and reporting of any suspected unexpected serious adverse reactions (SUSARs) to the regional ethics committee and to the Eudravigilance system, clinical trials module. Life-threatening or fatal SUSARs will be reported as soon as possible but at latest within 7 days after the sponsor has become aware of it. Relevant follow-up information will be reported within 8 additional days. Other SUSARs will be reported as soon as possible but at latest within 15 days after the sponsor has become aware of it.

18.6.1 Definition of reason to stop the trial

In case of an imminent risk for any of the study participants as defined by the investigator, the trial will be stopped until the risk has been identified and corrected. The trial will not start before approval from MPA. Patients suffering from any such risk shall be given medical care as needed within Stockholm County.

18.6.2 End of Trial Notification

A final report and end of trial notification shall be reported to LMV within 90 days as required by LVFS 2011;19, 9 kap.

19. STUDY MONITORING, AUDITING AND DOCUMENTATION

The PI and the study team will be trained prior to the start of the study. The study sites will be monitored on site. The site will be monitored as appropriate for the rate of enrollment in order to comply with GCP guidelines and to ensure validity of the study data. During each monitoring visit, consent forms will be reviewed, and source data verification will be performed to ensure compliance, including accurate and complete recording of data on CRFs, Source Records, and drug accountability records.

During or after the study, the regulatory authorities, the IRB, and/or representatives of the Sponsor may request access to all source documents, CRFs, and other protocol documentation for on-site audit or inspection.

All activities and responsibilities related to monitoring will be outlined in the study Monitoring Plan.

20. HUMAN SUBJECTS PROTECTIONS

20.1 Study Conduct

This study will be conducted in accordance with the protocol and with the following:

- Consensus ethical principles derived from international guidelines including the Declaration of Helsinki and Council for International Organizations of Medical Sciences (CIOMS) International Ethical Guidelines
- Applicable ICH GCP Guidelines
- Applicable laws and regulations
- The protocol, protocol amendments, ICFs, IB, and other relevant documents (e.g., advertisements) must be submitted to the IRB by the PI and reviewed and approved by the IRB before the study is initiated.
- Any amendments to the protocol will require IRB approval before implementation of changes made to the study design, except for changes necessary to eliminate an immediate hazard to study participants.

20.2 Principal Investigator Responsibilities

The PI will be responsible for the following:

- Providing written summaries of the status of the study to the IRB annually or more frequently in accordance with the requirements, policies, and procedures established by the IRB
- Notifying the IRB/ of SAEs, Unanticipated Problems or other significant safety findings as required by IRB procedures
- Providing oversight of the conduct of the study at the site and adherence to requirements of 21 CFR, ICH guidelines, the IRB, and all other applicable local regulations

20.3 Human Subjects Training

All study staff, to include any individual interacting with a participant or who has access to participant data, will be required to complete mandatory human subjects training prior to interacting with study participants.

20.4 Voluntary Participation

Participants will be informed, via the ICFs and the consent process, that participation is voluntary, that refusal to participate will involve no penalty or loss of benefits to which the participant would otherwise be entitled, and that the participant may discontinue participation at any time.

20.5 Benefits of Participation

There is no guarantee participants will receive a direct benefit from participating in this study. Study participation will include receiving a psychiatric and medical evaluation, including standard blood and urine-based safety laboratory tests. In addition, eligible participants will have a 50% chance of receiving an intervention (psilocybin) that has been reported in past studies to provide benefit for the treatment of symptoms. In addition, many individuals' depressive symptoms improve with placebo treatment.

Participants will have the chance to contribute to a scientific investigation, which may be of benefit to future patients, and may provide a sense of personal satisfaction in this regard. Benefits to others may include gaining significant knowledge regarding the potential utility of psilocybin for patients with MDD.

20.6 Alternatives to Participation

Potential participants may choose not to participate in this study. Individuals suffering with major depressive disorder (MDD), have at their potential disposal a wide range of treatments and/or procedures that are viable alternatives to participation in the current study. These alternatives are described in the regional medical guidelines for the treatment of MDD, available on www.psykiatristod.se

20.7 Study and Site Closure

The Sponsor designee reserves the right to close the study site or terminate the study at any time for any reason at the sole discretion of the Sponsor.

Study sites will be closed upon study completion. A study site is considered closed when all required documents and study supplies have been collected and a study-site closure visit has been performed.

The PI may initiate study-site closure at any time, provided reasonable cause and sufficient notice is given in advance of the intended termination.

Reasons for the early closure of a study site by the Sponsor or PI may include but are not limited to:

- Failure of the PI to comply with the protocol, the requirements of the IRB or local health authorities, the Sponsor's procedures, or GCP guidelines
- Inadequate participant recruitment
- Discontinuation of further study intervention development

20.8 Vulnerable Populations

Prisoners, pregnant women and mentally impaired persons or any other individuals considered a Vulnerable Population will not be enrolled in this study.

20.9 Cost to Participants

Aside from travel to and from the study site, there will be no costs to research participants for participating in this research study. As part of their study participation, study participants will receive study visits with labs/assessments, including medical and

psychiatric screening, preparatory sessions prior to dosing, dosing with psilocybin or active placebo, and integration sessions following dosing at no cost.

20.10 Participant Compensation

Table 6 provides an itemized list of payment for study participation. Compensation will be prorated based on the extent of participation completed.

Table 6: Participant Compensation

Compensation is to be given within two months from end of trial for the particular subject.

Activity	Compensation
Screening: Informed Consent; remaining Screening activities; Baseline assessment; preparation sessions	SEK 500
MRI1, PET1, Dosing session, MRI2, PET2, follow up assessments to day 42	SEK 2000
LP1 and 2	SEK 1000
Assesments day 43-365	SEK 1500
**TOTAL COMPENSATION**	SEK 5000

20.11 Treatment/ Compensation for Study Related Injury

The study participants are covered by Patientförsäkringen.

20.12 Protocol Deviations and Violations

All protocol violations and deviations must be addressed in study source and electronic documents, reported to the Sponsor, and must be sent to the site IRB per their guidelines. The site PI/study staff is responsible for knowing and adhering to their IRB requirements.

20.13 Record Retention

The PI must retain all study records required by the Sponsor and applicable ICH-GCP and MPA regulations in a secure and safe facility. The PI must consult a representative of the Sponsor before disposal of any study records. Essential documents are defined as documents that individually and collectively permit evaluation of the conduct of a trial and the quality of the data produced. These documents will be filed according to ICH-GCP regulations in the Investigator Site File (ISF).

20.14 Publication Policy

The Sponsor recognizes the importance of communicating medical study data and therefore encourages publications in reputable scientific journals and presentations at seminars or conferences.

- The results of this study may be published or presented at scientific meetings
- The Sponsor will comply with the requirements for publication of study results. In accordance with standard editorial and ethical practice, the Sponsor will generally

support publication of multicenter studies only in their entirety and not as individual site data. In this case, a coordinating PI will be designated by mutual agreement.

- Authorship will be determined by mutual agreement and in line with ICMJE authorship requirements.

21. CONFIDENTIALITY AND DATA SECURITY

21.1 Confidentiality and Data Security at Research Site

All participants will be seen in private rooms within a clinical or professional setting. Only the research coordinator, the Facilitators, and the participant will be present during the dosing session, with the exception of site PI and/or physician, as required.

Hard copies of study data will be kept securely, in a locked cabinet with limited and authorized access only.. Documents with identifiable information (including the informed consent) and blinding key will be filed separately from coded study data.

21.2 eCRF

The electronic Case Report File (eCRF) platform used for this study will be Microsoft Access using a two-factor authentication Office 365 cloud solution. Part of the data will be directly entered into the eCRF whereas other data will be initially captured on paper. The source data table will define what data are initially collected on a paper/eCRF.

21.3 Take Care and 1177

All information needed to comply with patientdatalagen will be documented in the Region Stockholm patient file system, Take Care (TC). Patient self-ratings will be performed via TC/1177.se and an *ansökan för uthämtning av personuppgifter* for extraction of study related data from TC will be performed before study start.

22. PREGNANCY

22.1 Definitions: Women of Childbearing Potential (WOCBP)

All individuals who were assigned biological sex of female at birth, and who have had no change in biological sex regardless of gender identification, will be considered Women of Childbearing Potential and will be required to have documented method of birth control and undergo urine pregnancy testing. For the purposes of this protocol, they will herein be referred to as “women”.

A woman is considered fertile following menarche and until becoming post-menopausal unless permanently sterile (see below).

Women in the following categories are not considered WOCBP:

1. Premenarchal

2. Premenopausal female with 1 of the following:
   - Documented hysterectomy
   - Documented bilateral salpingectomy
   - Documented bilateral oophorectomy

   Note: Documentation will be via self-report
3. Postmenopausal female
   - A postmenopausal state is defined as no menses for 12 months without an alternative medical cause. A high follicle stimulating hormone (FSH) level in the postmenopausal range may be used to confirm a postmenopausal state in women not using hormonal contraception or hormonal replacement therapy (HRT). However, in the absence of 12 months of amenorrhea, a single FSH measurement is insufficient.
   - Females on HRT and whose menopausal status is in doubt will be required to use one of the non-estrogen hormonal highly effective contraception methods if they wish to continue their HRT during the study. Otherwise, they must discontinue HRT to allow confirmation of postmenopausal status before study enrollment.

22.2 Contraception Guidance

22.2.1 Male participants

There are no restrictions on birth control choices/methods for male study participants.

22.2.2 Female participants

Women of childbearing potential are eligible to participate if they agree to use a highly effective method of contraception consistently and correctly as described in Table 7. Documentation of birth control method will be obtained at Screening and confirmed at all follow up visits. Changes in birth control method will be documented. If there was a lapse in birth control coverage, additional pregnancy testing may be warranted.

Table 7: Highly Effective Contraceptive Methods

**Highly Effective Contraceptive Methods That Are User Dependent**<br>*Failure rate of <1% per year when used consistently and correctly.*
Combined (estrogen and progestogen containing) hormonal contraception associated with inhibition of ovulation<br>• Oral<br>• Intravaginal<br>• Transdermal
Progestogen only hormonal contraception associated with inhibition of ovulation<br>• Oral<br>• Injectable
**Highly Effective Methods That Are User Independent** [a]
Implantable progestogen only hormonal contraception associated with inhibition of ovulation<br>• Intrauterine device (IUD)<br>• Intrauterine hormone-releasing system (IUS)<br>• Bilateral tubal occlusion

**Vasectomized partner**<br>*A vasectomized partner is a highly effective contraception method provided that the partner is the sole male sexual partner of the WOCBP and the absence of sperm has been confirmed. If not, an additional highly effective method of contraception should be used.*
**Sexual abstinence**<br>*Sexual abstinence is considered a highly effective method only if defined as refraining from heterosexual intercourse during the entire period of risk associated with the study intervention. The reliability of sexual abstinence needs to be evaluated in relation to the duration of the study and the preferred and usual lifestyle of the participant.*

22.3 Pregnancy Testing

- All WOCBP who were assigned biological sex of female at birth and have had no change in biological sex regardless of gender identification, will be required to undergo urine pregnancy testing.
- WOCBP should only be included after a negative highly sensitive urine pregnancy test.
- Pregnancy testing will be performed at Screening, Baseline, morning of dosing, Day 8, Day 42 and at any other time if it is suspected the participant may be pregnant.
- Pregnancy testing will be conducted using a commercial urine dipstick.

23. PREVIOUS EXPERIENCE

Johan Lundberg is head of the section for affective disorders, Norra Stockholms Psykiatri since 2013. He has since the PhD in the PET group 2006 developed his own line of research focusing on MDD, serotonin and glutamate, within the PET group where he is member of the board since 2014. He has supervised one PhD student to dissertation, and is currently main supervisor for three PhD students, co-supervisor for one and supervises two post-doc. Lundberg became associate professor of psychiatry at Karolinska Institutet in 2015 and research group leader 2017. Lundberg is PI for PETKET, a randomized double blind placebo controlled clinical trial of ketamine for the treatment of SSRI resistant MDD, and the effect on [11C]AZ10419369 PET quantification, and a series of potential response markers in peripheral blood (EudraCT 2017-001256-58), and has been PI for several previous PET studies on the effect of antidepressant treatment on markers for the mono amine system.

Co-PI Mikael Tiger, MD PhD, is an experienced psychiatrist, at post doc level with substantial experience within the field of MDD, antidepressant treatment and PET.

Co-PI Maria Beckman, lic psychologist, PhD, is an experienced psychologist, at post doc level with substantial experience in quantitative evaluation of behavioural interventions in psychiatry.

Co-investigator Predrag Petrovic, MD, PhD (2002), is specialist in psychiatry and associate professor in psychiatry and cognitive neuroscience. His background is in cognitive neuroscience and brain imaging including structural and functional fMRI. His research field is cognitive neuropsychiatry, i.e. the application of cognitive neuroscience to inform us on the mechanisms underlying psychiatric disorders. His main focus is on top-down regulation (including ADHD, emotional instability and bipolar disorder) as well as how our priors shape

our experience (including psychotic symptoms such as delusions and the placebo effect). His research group includes two PhD-students, one post-doc and one assistant professor (see below). He is also a co-supervisor for four PhD-students. He has previously been the main supervisor for two PhD- students and co-supervisor for eight PhD-students.

Co-investigator Alexander Lebedev, MD, PhD, is an assistant professor in cognitive neuroscience working in close collaboration with Predrag Petrovic as well as a trained psychiatrist in Russia. He has previously performed experimental research on higher order cognition related to aging. He has also published on the effects of psilocybin in the brain. His main research focus at present is psychosis related mechanisms in the brain as well as the cognitive neuroscience of psilocybin.

24. SUBSTANTIAL AMENDMENTS

Any substantial amendments in the protocol must be approved by the regional ethics committee and the regulatory authority (Läkemedelsverket) before implementation.

25. APPENDIXES

Appendix A - Full list of prohibited medications

26. REFERENCES

1. Ross, S. *et al.* Rapid and sustained symptom reduction following psilocybin treatment for anxiety and depression in patients with life-threatening cancer: a randomized controlled trial. *J. Psychopharmacol. Oxf. Engl.* **30**, 1165–1180 (2016).
2. Carhart-Harris, R. L. *et al.* Psilocybin with psychological support for treatment-resistant depression: an open-label feasibility study. *Lancet Psychiatry* **3**, 619–627 (2016).
3. The changing rate of major depression. Cross-national comparisons. Cross-National Collaborative Group. *JAMA* **268**, 3098–3105 (1992).
4. Chengappa, K. N. R. *et al.* Relationship of birth cohort and early age at onset of illness in a bipolar disorder case registry. *Am. J. Psychiatry* **160**, 1636–1642 (2003).
5. Global Burden of Disease Study 2013 Collaborators. Global, regional, and national incidence, prevalence, and years lived with disability for 301 acute and chronic diseases and injuries in 188 countries, 1990-2013: a systematic analysis for the Global Burden of Disease Study 2013. *Lancet Lond. Engl.* **386**, 743–800 (2015).
6. Frank, E. *et al.* Three-year outcomes for maintenance therapies in recurrent depression. *Arch. Gen. Psychiatry* **47**, 1093–1099 (1990).
7. Greden, J. F. The burden of disease for treatment-resistant depression. *J. Clin. Psychiatry* **62 Suppl 16**, 26–31 (2001).
8. Judd, L. L. *et al.* A prospective 12-year study of subsyndromal and syndromal depressive symptoms in unipolar major depressive disorders. *Arch. Gen. Psychiatry* **55**, 694–700 (1998).
9. Wulsin, L. R., Vaillant, G. E. & Wells, V. E. A systematic review of the mortality of depression. *Psychosom. Med.* **61**, 6–17 (1999).

10. Joynt, K. E., Whellan, D. J. & O'Connor, C. M. Depression and cardiovascular disease: mechanisms of interaction. *Biol. Psychiatry* **54**, 248–261 (2003).
11. Davidson, K., Jonas, B. S., Dixon, K. E. & Markovitz, J. H. Do depression symptoms predict early hypertension incidence in young adults in the CARDIA study? Coronary Artery Risk Development in Young Adults. *Arch. Intern. Med.* **160**, 1495–1500 (2000).
12. Eaton, W. W., Armenian, H., Gallo, J., Pratt, L. & Ford, D. E. Depression and risk for onset of type II diabetes. A prospective population-based study. *Diabetes Care* **19**, 1097–1102 (1996).
13. Barefoot, J. C. *et al.* Symptoms of depression and changes in body weight from adolescence to mid-life. *Int. J. Obes. Relat. Metab. Disord. J. Int. Assoc. Study Obes.* **22**, 688–694 (1998).
14. Spiegel, D. & Giese-Davis, J. Depression and cancer: mechanisms and disease progression. *Biol. Psychiatry* **54**, 269–282 (2003).
15. Miller, I. W. *et al.* The treatment of chronic depression, part 3: psychosocial functioning before and after treatment with sertraline or imipramine. *J Clin Psychiatry* **59**, 608–19 (1998).
16. Simon, G. E. Long-term prognosis of depression in primary care. *Bull. World Health Organ.* **78**, 439–445 (2000).
17. Judd, L. L. *et al.* Does incomplete recovery from first lifetime major depressive episode herald a chronic course of illness? *Am. J. Psychiatry* **157**, 1501–1504 (2000).
18. Svenningsson, P. *et al.* Alterations in 5-HT1B receptor function by p11 in depression-like states. *Science* **311**, 77–80 (2006).
19. Svenningsson, P. *et al.* Preliminary evidence that early reduction in p11 levels in natural killer cells and monocytes predicts the likelihood of antidepressant response to chronic citalopram. *Mol. Psychiatry* **19**, 962–964 (2014).
20. Elfving, B. *et al.* Inverse correlation of brain and blood BDNF levels in a genetic rat model of depression. *Int. J. Neuropsychopharmacol.* **13**, 563–572 (2010).
21. Deyama, S. & Duman, R. S. Neurotrophic mechanisms underlying the rapid and sustained antidepressant actions of ketamine. *Pharmacol. Biochem. Behav.* **188**, 172837 (2020).
22. Maffioletti, E. *et al.* Genetic determinants of circulating VEGF levels in major depressive disorder and electroconvulsive therapy response. *Drug Dev. Res.* (2020) doi:10.1002/ddr.21658.
23. Duman, R. S. & Monteggia, L. M. A neurotrophic model for stress-related mood disorders. *Biol. Psychiatry* **59**, 1116–1127 (2006).
24. Hansson, A. C., Rimondini, R., Heilig, M., Mathé, A. A. & Sommer, W. H. Dissociation of antidepressant-like activity of escitalopram and nortriptyline on behaviour and hippocampal BDNF expression in female rats. *J. Psychopharmacol. Oxf. Engl.* **25**, 1378–1387 (2011).
25. Price, J. B. *et al.* Bioenergetics and synaptic plasticity as potential targets for individualizing treatment for depression. *Neurosci. Biobehav. Rev.* **90**, 212–220 (2018).
26. Liu, R.-J. & Aghajanian, G. K. Stress blunts serotonin- and hypocretin-evoked EPSCs in prefrontal cortex: role of corticosterone-mediated apical dendritic atrophy. *Proc. Natl. Acad. Sci. U. S. A.* **105**, 359–364 (2008).
27. Rajkowska, G. *et al.* Morphometric evidence for neuronal and glial prefrontal cell pathology in major depression. *Biol. Psychiatry* **45**, 1085–1098 (1999).
28. Kang, H. J. *et al.* Decreased expression of synapse-related genes and loss of synapses in major depressive disorder. *Nat. Med.* **18**, 1413–1417 (2012).

29. Treadway, M. T. *et al.* Illness progression, recent stress, and morphometry of hippocampal subfields and medial prefrontal cortex in major depression. *Biol. Psychiatry* **77**, 285–294 (2015).
30. Li, N. *et al.* mTOR-dependent synapse formation underlies the rapid antidepressant effects of NMDA antagonists. *Science* **329**, 959–964 (2010).
31. Bajjalieh, S. M., Peterson, K., Shinghal, R. & Scheller, R. H. SV2, a brain synaptic vesicle protein homologous to bacterial transporters. *Science* **257**, 1271–1273 (1992).
32. Vogl, C. *et al.* Synaptic vesicle glycoprotein 2A modulates vesicular release and calcium channel function at peripheral sympathetic synapses. *Eur. J. Neurosci.* **41**, 398–409 (2015).
33. Gillard, M., Chatelain, P. & Fuks, B. Binding characteristics of levetiracetam to synaptic vesicle protein 2A (SV2A) in human brain and in CHO cells expressing the human recombinant protein. *Eur. J. Pharmacol.* **536**, 102–108 (2006).
34. Kaufman, A. C. *et al.* Fyn inhibition rescues established memory and synapse loss in Alzheimer mice. *Ann. Neurol.* **77**, 953–971 (2015).
35. Husum, H., Bolwig, T. G., Sánchez, C., Mathé, A. A. & Hansen, S. L. Levetiracetam prevents changes in levels of brain-derived neurotrophic factor and neuropeptide Y mRNA and of Y1- and Y5-like receptors in the hippocampus of rats undergoing amygdala kindling: implications for antiepileptogenic and mood-stabilizing properties. *Epilepsy Behav. EB* **5**, 204–215 (2004).
36. Nabulsi, N. B. *et al.* Synthesis and Preclinical Evaluation of 11C-UCB-J as a PET Tracer for Imaging the Synaptic Vesicle Glycoprotein 2A in the Brain. *J. Nucl. Med. Off. Publ. Soc. Nucl. Med.* **57**, 777–784 (2016).
37. Finnema, S. J. *et al.* Imaging synaptic density in the living human brain. *Sci. Transl. Med.* **8**, 348ra96 (2016).
38. Holmes, S. E. *et al.* Lower synaptic density is associated with depression severity and network alterations. *Nat. Commun.* **10**, 1529 (2019).
39. Carhart-Harris, R. L. *et al.* The entropic brain: a theory of conscious states informed by neuroimaging research with psychedelic drugs. *Front. Hum. Neurosci.* **8**, 20 (2014).
40. Nichols, D. E. Hallucinogens. *Pharmacol. Ther.* **101**, 131–181 (2004).
41. Passie, T., Seifert, J., Schneider, U. & Emrich, H. M. The pharmacology of psilocybin. *Addict. Biol.* **7**, 357–364 (2002).
42. Carhart-Harris, R. L. & Nutt, D. J. Experienced drug users assess the relative harms and benefits of drugs: a web-based survey. *J. Psychoactive Drugs* **45**, 322–328 (2013).
43. Nutt, D. J., King, L. A., Phillips, L. D. & Independent Scientific Committee on Drugs. Drug harms in the UK: a multicriteria decision analysis. *Lancet Lond. Engl.* **376**, 1558–1565 (2010).
44. Madsen, M. K. *et al.* Psychedelic effects of psilocybin correlate with serotonin 2A receptor occupancy and plasma psilocin levels. *Neuropsychopharmacol. Off. Publ. Am. Coll. Neuropsychopharmacol.* **44**, 1328–1334 (2019).
45. Ly, C. *et al.* Psychedelics Promote Structural and Functional Neural Plasticity. *Cell Rep.* **23**, 3170–3182 (2018).
46. Griffiths, R. R. *et al.* Psilocybin produces substantial and sustained decreases in depression and anxiety in patients with life-threatening cancer: A randomized double-blind trial. *J. Psychopharmacol. Oxf. Engl.* **30**, 1181–1197 (2016).
47. Griffiths, R. R. *et al.* Psilocybin occasioned mystical-type experiences: immediate and persisting dose-related effects. *Psychopharmacology (Berl.)* **218**, 649–665 (2011).
48. Griffiths, R. R., Richards, W. A., McCann, U. & Jesse, R. Psilocybin can occasion mystical-type experiences having substantial and sustained personal meaning and

spiritual significance. *Psychopharmacology (Berl.)* **187**, 268–283; discussion 284-292 (2006).
49. Studerus, E., Gamma, A., Kometer, M. & Vollenweider, F. X. Prediction of psilocybin response in healthy volunteers. *PloS One* **7**, e30800 (2012).
50. Carmody, T. J. *et al.* The Montgomery Asberg and the Hamilton ratings of depression: a comparison of measures. *Eur. Neuropsychopharmacol. J. Eur. Coll. Neuropsychopharmacol.* **16**, 601–611 (2006).
51. European Medicines Agency. Clinical investigation of medicinal products in the treatment of depression. (2013).
52. Montgomery, S. A. & Asberg, M. A new depression scale designed to be sensitive to change. *Br J Psychiatry* **134**, 382–9. (1979).
53. Daly, E. J. *et al.* Efficacy and Safety of Intranasal Esketamine Adjunctive to Oral Antidepressant Therapy in Treatment-Resistant Depression: A Randomized Clinical Trial. *JAMA Psychiatry* **75**, 139–148 (2018).
54. Swedish Council on Health Technology Assessment. *Instruments for Suicide Risk Assessment*. (Swedish Council on Health Technology Assessment (SBU), 2015).
55. Svanborg, P. & Asberg, M. A comparison between the Beck Depression Inventory (BDI) and the self-rating version of the Montgomery Asberg Depression Rating Scale (MADRS). *J. Affect. Disord.* **64**, 203–216 (2001).
56. Sheehan, K. H. & Sheehan, D. V. Assessing treatment effects in clinical trials with the discan metric of the Sheehan Disability Scale. *Int. Clin. Psychopharmacol.* **23**, 70–83 (2008).
57. 16717171.
58. Dear, B. F. *et al.* Psychometric comparison of the generalized anxiety disorder scale-7 and the Penn State Worry Questionnaire for measuring response during treatment of generalised anxiety disorder. *Cogn. Behav. Ther.* **40**, 216–27 (2011).
59. Spitzer, R. L., Kroenke, K., Williams, J. B. & Lowe, B. A brief measure for assessing generalized anxiety disorder: the GAD-7. *Arch. Intern. Med.* **166**, 1092–7 (2006).
60. Williams, N. The GAD-7 questionnaire. *Occup. Med.* **64**, 224–224 (2014).
61. Guy, W. *ECDEU assessment manual for psychopharmacology publication*. (Department of health, education and welfare, 1976).
62. Sobocki, P. *et al.* Health-related quality of life measured with EQ-5D in patients treated for depression in primary care. *Value Health J. Int. Soc. Pharmacoeconomics Outcomes Res.* **10**, 153–160 (2007).
63. Barrett, F. S., Bradstreet, M. P., Leoutsakos, J. S., Johnson, M. W. & Griffiths, R. R. The Challenging Experience Questionnaire: Characterization of challenging experiences with psilocybin mushrooms. *J Psychopharmacol* **30**, 1279–1295 (2016).
64. Stace, W. T. *Mysticism and Philosophy.* (MacMillan Press, 1960).
65. Bogenschutz, M. P. *et al.* Psilocybin-assisted treatment for alcohol dependence: a proof-of-concept study. *J Psychopharmacol* **29**, 289–99 (2015).
66. Albert Garcia-Romeu, Roland R. Griffiths & Matthew W. Johnson. Psilocybin-occasioned Mystical Experiences in the Treatment of Tobacco Addiction. *Curr Drug Abuse Rev* **7**, 157–164 (2015).
67. Griffiths, R., Richards, W., Johnson, M., McCann, U. & Jesse, R. Mystical-type experiences occasioned by psilocybin mediate the attribution of personal meaning and spiritual significance 14 months later. *J Psychopharmacol* **22**, 621–32 (2008).
68. Johnson, M. W., Garcia-Romeu, A., Cosimano, M. P. & Griffiths, R. R. Pilot study of the 5-HT2AR agonist psilocybin in the treatment of tobacco addiction. *J Psychopharmacol* **28**, 983–92 (2014).

69. Maclean, K. A., Leoutsakos, J. M., Johnson, M. W. & Griffiths, R. R. Factor Analysis of the Mystical Experience Questionnaire: A Study of Experiences Occasioned by the Hallucinogen Psilocybin. *J Sci Study Relig* **51**, 721–737 (2012).
70. Richards, W. A. Counseling, Peak Experiences and the Human Encounter with Death: An Empirical Study of the Efficacy of DPT-Assitance Counseling in Enhancing Quality of Life of Persons with Terminal Cancer and Their Closest Family Members. (1975).
71. Bond, F. W. *et al.* Preliminary psychometric properties of the Acceptance and Action Questionnaire-II: a revised measure of psychological inflexibility and experiential avoidance. *Behav. Ther.* **42**, 676–88 (2011).
72. Hayes, S. C., Strosahl, K. D. & Wilson, K. G. *Acceptance and commitment therapy: The process and practice of mindful change*. (Guilford Press, 2012).
73. Fledderus, M., Oude Voshaar, M. A., Ten Klooster, P. M. & Bohlmeijer, E. T. Further evaluation of the psychometric properties of the Acceptance and Action Questionnaire-II. *Psychol. Assess.* **24**, 925–36 (2012).
74. Wolgast, M. What does the Acceptance and Action Questionnaire (AAQ-II) really measure? *Behav. Ther.* **45**, 831–9 (2014).
75. Close, J. B., Hajien, E. C., Watts, R., Roseman, L. & Carhart-Harris, R. L. Psychedelics and psychological flexibility – Results of a prospective web-survey using the Acceptance in Action Questionnaire II. *J. Context. Behav. Sci.* (2020) doi:10.1016/j.jcbs.2020.01.005.
76. Davis, A. K., Barrett, F. S. & Griffiths, R. R. Psychological flexibility mediates the relations between acute psychedelic effects and subjective decreases in depression and anxiety. *J. Context. Behav. Sci.* **15**, 39–45 (2020).
77. Luoma, J. B., Davis, A. K., Watts, R. & McCracken, L. M. Integrating contextual behavioral science with research on psychedelic assisted therapy: Introduction to the special section. *J. Context. Behav. Sci.* **15**, 207–209 (2020).
78. Steger, M. F., Frazier, P., Oishi, S. & Kaler, M. The meaning in life questionnaire: Assessing the presence of and search for meaning in life. *J. Couns. Psychol.* **53**, 80–93 (2006).
79. Diener, E., Emmons, R. A., Larsen, R. J. & Griffin, S. The Satisfaction With Life Scale. *J. Pers. Assess.* **49**, 71–5 (1985).
80. Horvath, A. O. & Greenberg, L. S. Development and validation of the Working Alliance Inventory. *J. Couns. Psychol.* **36**, 223–233 (1989).
81. Tracey, T. J. & Kokotovic, A. M. Factor structure of the Working Alliance Inventory. *Psychol. Assess. J. Consult. Clin. Psychol.* **1**, 207–210 (1989).
82. Bordin, E. The generalizability of the psychoanalytic concept of the working alliance. *Psychother. Theory Res. Pract.* **16**, 252–260 (1979).
83. Moyers, Martin, Manuel, Hendrickson & Miller. Assessing competence in the use of motivational interviewing. *J. Subst. Abuse Treat.* **28**, 19–26 (2005).
84. Glynn, L. H. & Moyers, T. B. Manual for the client language easy rating (CLEAR) coding system: Formely "Motivational Interviewing Skill Code (MISC) 1.1" Retrieved from http://casaa.unm.edu/codinginst.html. (2012).
85. Magill, M. *et al.* A meta-analysis of motivational interviewing process: Technical, relational, and conditional process models of change. *J. Consult. Clin. Psychol.* **86**, 140–157 (2018).
86. Villarosa-Hurlocker, M. C., O'Sickey, A. J., Houck, J. M. & Moyers, T. B. Examining the influence of active ingredients of motivational interviewing on client change talk. *J. Subst. Abuse Treat.* **96**, 39–45 (2019).

87. Sheehan, D. V. *et al.* The Mini-International Neuropsychiatric Interview (M.I.N.I.): the development and validation of a structured diagnostic psychiatric interview for DSM-IV and ICD-10. *J Clin Psychiatry* **59 Suppl 20**, 22-33;quiz 34-57 (1998).
88. Andrews, P. W., Kornstein, S. G., Halberstadt, L. J., Gardner, C. O. & Neale, M. C. Blue again: perturbational effects of antidepressants suggest monoaminergic homeostasis in major depression. *Front. Psychol.* **2**, 159 (2011).
89. Caddy, C. *et al.* Ketamine and other glutamate receptor modulators for depression in adults. in *Cochrane Database of Systematic Reviews* (John Wiley & Sons, Ltd, 2015).
90. Moreno, C. *et al.* Depression in bipolar disorder versus major depressive disorder: results from the National Epidemiologic Survey on Alcohol and Related Conditions. *Bipolar Disord.* **14**, 271–282 (2012).
91. Alexopoulos, G. S., Young, R. C. & Meyers, B. S. Geriatric depression: age of onset and dementia. *Biol. Psychiatry* **34**, 141–145 (1993).
92. Hsieh, M.-H. *et al.* Hippocampal volume and antidepressant response in geriatric depression. *Int. J. Geriatr. Psychiatry* **17**, 519–525 (2002).
93. Kalayam, B. & Alexopoulos, G. S. Prefrontal dysfunction and treatment response in geriatric depression. *Arch. Gen. Psychiatry* **56**, 713–718 (1999).
94. Rucker, J. J. H., Iliff, J. & Nutt, D. J. Psychiatry & the psychedelic drugs. Past, present & future. *Neuropharmacology* **142**, 200–218 (2018).
95. Raison, C. L. *et al.* Depressive symptoms and viral clearance in patients receiving interferon-alpha and ribavirin for hepatitis C. *Brain. Behav. Immun.* **19**, 23–27 (2005).
96. Whelton, P. K. *et al.* 2017 ACC/AHA/AAPA/ABC/ACPM/AGS/APhA/ASH/ASPC/NMA/PCNA Guideline for the Prevention, Detection, Evaluation, and Management of High Blood Pressure in Adults: A Report of the American College of Cardiology/American Heart Association Task Force on Clinical Practice Guidelines. *J. Am. Coll. Cardiol.* **71**, e127–e248 (2018).
97. Bergstrom, M. *et al.* Head fixation device for reproducible position alignment in transmission CT and positron emission tomography. *J Comput Assist Tomogr* **5**, 136–41. (1981).
98. van der Aart, J., Hallett, W. A., Rabiner, E. A., Passchier, J. & Comley, R. A. Radiation dose estimates for carbon-11-labelled PET tracers. *Nucl. Med. Biol.* **39**, 305–314 (2012).
99. Varrone, A. *et al.* Advancement in PET quantification using 3D-OP-OSEM point spread function reconstruction with the HRRT. *Eur J Nucl Med Mol Imaging* **36**, 1639–50 (2009).
100. Schain, M. *et al.* Quantification of serotonin transporter availability with [11C]MADAM--a comparison between the ECAT HRRT and HR systems. *NeuroImage* **60**, 800–807 (2012).
101. Savitz, J. B. & Drevets, W. C. Neuroreceptor imaging in depression. *Neurobiol. Dis.* **52**, 49–65 (2013).
102. Nasca, C. *et al.* L-acetylcarnitine causes rapid antidepressant effects through the epigenetic induction of mGlu2 receptors. *Proc. Natl. Acad. Sci.* **110**, 4804–4809 (2013).
103. Stroth, N. & Svenningsson, P. S100B interacts with the serotonin 5-HT7 receptor to regulate a depressive-like behavior. *Eur. Neuropsychopharmacol. J. Eur. Coll. Neuropsychopharmacol.* **25**, 2372–2380 (2015).
104. Tiger, M. *et al.* No correlation between serotonin and its metabolite 5-HIAA in the cerebrospinal fluid and [(11) C]AZ10419369 binding measured with PET in healthy volunteers. *Synap. N. Y. N* **68**, 480–483 (2014).

105. Espiard, M.-L., Lecardeur, L., Abadie, P., Halbecq, I. & Dollfus, S. Hallucinogen persisting perception disorder after psilocybin consumption: a case study. *Eur. Psychiatry J. Assoc. Eur. Psychiatr.* **20**, 458–460 (2005).
106. Studerus, E., Kometer, M., Hasler, F. & Vollenweider, F. X. Acute, subacute and long-term subjective effects of psilocybin in healthy humans: a pooled analysis of experimental studies. *J. Psychopharmacol. Oxf. Engl.* **25**, 1434–1452 (2011).
107. Brown, E., Moller, N. & Ramsey-Wade, C. Recording therapy sessions: What do clients and therapists really think? *Couns. Psychother. Res.* **13**, 254–262 (2013).
108. Ellis, M. V. Bridging the Science and Practice of Clinical Supervision: Some Discoveries, Some Misconceptions. *Clin. Superv.* **29**, 95–116 (2010).
109. Johnson, M. W., Griffiths, R. R., Hendricks, P. S. & Henningfield, J. E. The abuse potential of medical psilocybin according to the 8 factors of the Controlled Substances Act. *Neuropharmacology* **142**, 143–166 (2018).
110. Fantegrossi, W. E., Woods, J. H. & Winger, G. Transient reinforcing effects of phenylisopropylamine and indolealkylamine hallucinogens in rhesus monkeys. *Behav. Pharmacol.* **15**, 149–157 (2004).
111. Griffiths, R. R. Common factors in human and infrahuman drug self-administration. *Psychopharmacol. Bull.* **16**, 45–47 (1980).
112. Poling, A. & Bryceland, J. Voluntary drug self-administration by nonhumans: a review. *J. Psychedelic Drugs* **11**, 185–190 (1979).
113. O'Brien, C. Addiction and dependence in DSM-V. *Addict. Abingdon Engl.* **106**, 866–867 (2011).

Statistical Analysis Plan (SAP)

The Effect of Psilocybin on MDD Symptom Severity and Synaptic Density – A Single Dose Randomized, Double Blind, Placebo- Controlled Phase 2 Positron Emission Tomography Study

September 9, 2020

Version 1.2 Final Version

STATISTIK AKADEMIN

APPROVAL

This document has been reviewed and approved by:		
Anders Berglund, PhD		7/9-2020
Biostatistician	Signature	Date
Johan Lundberg, Associate professor, MD, PhD		
Principle Investigator	Signature	Date

Table of Contents

1. Introduction 4
2. Study objectives 4
2.1. Primary Objectives 5
2.2. Secondary Objectives 5
2.3. Exploratory Objectives 5
2.4. Safety Objectives 5
3. Study Endpoints 5
3.1. Primary Endpoint 5
3.2. Secondary Endpoints 5
3.3. Exploratory Endpoints 6
3.4. Safety Endpoints 7
4. Study Design 8
5. Statistical analysis 8
5.1. Power analysis and Sample Size Determination 8
5.2. General statistical considerations 9
5.3. Descriptive statistics 10
5.4. Primary analysis 10
5.5. Sensitivity analysis 11
5.6. Exploratory analysis 11
5.7. Safety analysis 11

1. Introduction

The overarching purpose of this study is to conduct an initial examination of the safety, efficacy, tolerability and MDD relevant biological effects of single-dose psilocybin (3-[2- (dimethylamino)ethyl]-1H-indol-4-yl] dihydrogen phosphate) in otherwise medically healthy patients with recurrent MDD. The overall objectives of this study are to examine the efficacy of a single, fixed, 25 mg oral dose of psilocybin in reducing depressive symptoms and improving functional disability and quality of life in medically healthy patients with recurrent MDD when compared to active placebo; to compare the effect of biological markers of MDD in blood, CSF and using PET and MRI; to collect safety and tolerability data on single-dose psilocybin intervention in medically healthy patients with MDD, and to evaluate Facilitators' and participants' in-session behaviors on treatment outcome.

MDD is currently the leading cause of disability in the world. This highlights the urgent need to identify and test novel pharmacological agents that might benefit depressed patients who have not achieved symptom remission with currently approved antidepressant modalities. Recent studies suggest that psilocybin produces a substantial and sustained improvement in depressive symptoms following a single administration. It also highlights the need to identify the biological underpinning related to response to these novel treatments, in order to identify response markers and pave the way for rational drug development of novel antidepressant drugs.

2. Study objectives

The primary objective of this study is to evaluate the efficacy of a single 25 mg oral dose of psilocybin for recurrent major depressive disorder (MDD) compared to an active placebo (niacin) in otherwise medically-healthy participants ages 20 to 65, assessed as the difference between groups in changes in depressive symptoms.

2.1. Primary Objectives

The change in blinded rater Montgomery-Asberg Depression Rating Scale (MADRS) total score from Baseline to Day 8.

2.2. Secondary Objectives

The secondary objective is to evaluate the effect of psilocybin on synaptic density in prefrontal cortex and hippocampus, by comparing the change in [11C]UCB-J binding after psilocybin and placebo dosing.

2.3. Exploratory Objectives

The exploratory objectives of this study are to evaluate the efficacy of a single 25 mg dose of psilocybin for MDD compared to an active placebo in medically-healthy participants ages 20 to 65.

2.4. Safety Objectives

The overall safety objective of this study is to evaluate a single 25 mg oral dose of psilocybin compared to an active placebo in incidence, severity and frequency of Adverse Events (AEs), Treatment Emergent AEs (TEAEs), Solicited AEs, and Serious Adverse Events (SAEs) before, during and after the dosing session and at all follow-up visits.

3. Study Endpoints

3.1. Primary Endpoint

Between-group difference in change of blinded rater Montgomery-Asberg Depression Rating Scale (MADRS) total score from Baseline to post-dose Day 8.

3.2. Secondary Endpoints

The following secondary endpoints will be evaluated:

- Change in MADRS total score from Baseline to post-dose Day 15, 42, 180 and 365 (end of study) respectively
- Change in MADRS total score from Baseline to the treatment period (mean of Day 8-365)
- Change in MADRS-S score from Baseline to Day 365 (end of study)
- Change in MADRS-S score from Baseline to the treatment period (mean of Day 8-365)
- Response rate at all time points post dose (MADRS and MADRS-S)
- Remission rate at all time points post dose (MADRS and MADRS-S)
- Change in Sheehan Disability Scale (SDS) score from Baseline to Day 365 (end of study)
- Change in SDS score from Baseline to the treatment period (mean of Day 8-365)
- Change in Clinical Global Impression (CGI) score from Baseline to Day 8, 42, 180 and 365 (end of study) respectively
- Change in CGI score from Baseline to the treatment period (mean of Day 8-365)
- Time to initiation of antidepressant treatment

3.3. Exploratory Endpoints

Exploratory analysis will be between-group differences in:

- Change in p11 concentration in PBMC subsets from Baseline to day 15 Post dose
- Change in BDNF and VEGF concentration in plasma from Baseline to day 15 Post Dose
- Change in kynurenic acid in CSF from Baseline to day 15 Post dose
- Change in monoamine and monoamine metabolites in CSF from Baseline to day 15 Post dose
- Change in cytokine concentration (exact markers to be determined based on current literature at time of analysis) in CSF from Baseline to day 15 Post dose

- Change in metrics of whole-brain connectomics and hub characteristics of medial and ventrolateral prefrontal cortex as well as orbitofrontal cortex in the psilocybin treated group as compared to the placebo group, and in relation to clinical outcome (MADRS) as well as PET-outcome (resting state fMRI).
- Change in influence of top-down priors in an instructed fear learning paradigm and its relation to a change in activity of the ventromedial prefrontal cortex and lateral orbitofrontal cortex in the psilocybin treated group as compared to the placebo group, and in relation to clinical outcome (MADRS) as well as PET-outcome (tasked based fMRI).
- Change in Emotion detection under perceptual uncertainty and perspective-shifting ability in relation to large-scale network activity patterns in the psilocybin treated group as compared to the placebo group, and in relation to clinical outcome (MADRS) as well as PET-outcome.
- Change in anxiety symptoms from Baseline to post-dose Day 8, 15, 42 and 365
- Change in functional disability to post-dose Day 8, 15, 42 and 365
- Change in health-related meaning of life and satisfaction with life from Baseline to post-dose Day 8, 15, 42 and 365.
- Change in psychological flexibility from Baseline to post-dose Day 8, 15, 42 and 365
- Correlations between biomarkers and scales

3.4. Safety Endpoints

Differences between the psilocybin and active placebo groups in:

- Incidence of AEs by severity
- Incidence of AEs leading to termination from the study
- Incidence of TEAEs
- Incidence of TEAEs by severity
- Incidence of SAEs

4. Study Design

Thirty patients (males and females) ages 20 to 65 who, at Screening, meet ICD-10 criteria for recurrent MDD with a current depressive episode of at least a 30-day duration, an on-site Screening MADRS score 22 (consistent with moderate or greater depressive symptom severity), 30% symptom decrease from web-screen, and who meet all other inclusion/exclusion criteria at Baseline will be enrolled into the study and randomized with a 1-to-1 allocation under double-blind conditions to receive a single 25 mg oral dose of psilocybin or a single 100 mg oral dose of niacin. Expecting a maximum of 20% drop out rate a maximum of 6 more subjects may be randomized, in order to reach 30 patients completed (performed all study related activities up until and including PET#2 day 15). Niacin will serve as an active placebo that provides an acute physiological response (flushing) that may aid in blinding of intervention allocation. All randomized participants will be included in the Full Analysis Set (FAS) that will be used for analyzing primary and secondary study endpoints.

Randomization will occur for participants who have been determined to be eligible for the study and will occur the morning prior to dosing. Following this, participants will be randomized to one of two intervention groups. Participants are randomized in blocks in a 1:1 ratio in a blinded fashion to the psilocybin group or the active control group. The block size will not be shared. Randomization will be done at the site by strictly following a randomization list in sequential order. If a patient is replaced the new patient will be allocated a randomization number with the same treatment while keeping the double blinded allocation. This will be performed by having back-up blinded treatment allocations for each subject in sealed envelopes. These will only be used and opened following withdrawal. Importantly, the allocated treatment will remain blinded for both the withdrawn and replacement subject as well as for the study team.

5. Statistical analysis

5.1. Power analysis and Sample Size Determination

The difference between psilocybin and placebo in change from baseline to Day 8 in MADRS total score (primary endpoint) will be analyzed for the Full Analysis Set using Analysis of Covariance including treatment as main effect and baseline as covariate.

Based on prior controlled studies of psilocybin in cancer- related depression and anxiety, a decrease in depression symptoms from baseline to one-week post-dose of at least 30% is expected in the placebo

arm. In an open-label study of psilocybin the change in MADRS total score from baseline to 1 week was -23.3. Several clinical studies in the literature report a standard deviation in change in MADRS total score around 10.

A sample size of 15 in each group will have 80% power to detect a difference in means of 11 (the difference between a psilocybin mean, of -23 and a placebo mean, of -12) assuming that the common standard deviation is 10 using a two group t-test with a 5% two sided significance level.

Considering the short duration between treatment and primary endpoint measurement day 8 the risk of withdrawal is considered low. The total sample size is set to 30 patients completing day 15. In case of withdrawals before day 15 additional patients may be randomized to ensure 30 patients included in the primary and secondary analyses. Estimating a maximum of 20% withdrawals, a maximum of 6 additional subjects can be included.

5.2. General statistical considerations

Efficacy analyses of primary, secondary, and exploratory outcomes will be conducted using the Full Analysis Set (FAS) population and the Safety set will be used in all safety analyses. All participants randomized to receive a study intervention (psilocybin vs. active placebo) will be included in FAS. All participants randomized to receive a study intervention (psilocybin vs. active placebo) with no relevant protocol deviations will be included in per protocol (PP). The Per Protocol set will be used in sensitivity analyses for the primary and secondary objectives. All participants who have received study treatment will comprise the overall safety set.

All reported p values will be two-sided, and $p<0.05$ will be considered statistically significant. However, any hypothesis that includes a direction of change will be tested using one-sided test using a significance level of 0.025. The analysis will not be adjusted for multiple comparisons and the analysis should be described and interpreted as exploratory analysis. All data management and analyses will be performed using SAS 9.4 and R version 3.6.1.

5.3. Descriptive statistics

Demographic, diagnostic, baseline, line of therapy and safety data will be presented using descriptive statistics. Continuous variables will be described by the number of patients with a recorded value, mean with standard deviation and when appropriate also 95% confidence interval (CI), median, upper and lower quartiles and maximum and minimum values. Categorical variables will be reported as number and percentages with 95% CIs. Differences between subgroups will be compared using chi-square tests, and fisher's test if few cases, for categorical variables and using t-test, and non-parametric statistics, for continuous variables. Distributional features of all measurements will be evaluated, and data transformation (e.g., logarithm, rank transform) will be performed when applicable. Missing data will be described in all summaries.

5.4. Primary analysis

The primary endpoint is change from baseline to Day 8 in MADRS total score. The primary analysis will be a mixed-model repeated measures (MMRM) model, fitting treatment, timepoint, and a treatment*timepoint interaction. An unstructured covariance matrix will be used to estimate the within subject variation and the Kenward-Roger approximation will be used to estimate denominator degrees of freedom. If this analysis fails to converge, the following structures will be used in order: heterogeneous Toeplitz (TOEPH), Antedependence (ANTE1), heterogeneous autoregressive (ARH1), Autoregressive (AR1) and Compound symmetry (CS). The first covariance structure yielding convergence will be used. If a structured covariance is used, the empirical "sandwich" estimator of the standard error (SE) of the fixed effects parameters will be used to deal with possible model misspecification of the covariance matrix. The "least squares" mean (LS mean) estimates of treatment differences and their 95% CIs will be presented for each time point. In addition, a p-value will be presented for the primary comparisons. The LS mean change from baseline to each post-baseline will be plotted by treatment group. To examine the pattern of missing data, the number and percentage of subjects with a mean MADRS score will be presented by treatment group. Summary statistics for MADRS score and change from baseline will also be presented, based on observed scores (no imputation of missing data) calculated from all available data. These summaries will also be produced for the subset of subjects who have a non-missing MADRS score.

5.5. Sensitivity analysis

The secondary endpoints evaluating changes in (MADRS, MADR-S, SDS, and CGI) score during different/several time points will be conducing by using MMRM models, fitting treatment, timepoint, and a treatment*timepoint interaction. The models will be built as of the primary endpoint (see 5.4 Primary analysis). Paired t-test will be performed for between-groups comparisons at all time points, and between all time points over time against baseline. Response will be defined as ≥ 50% decrease (MADRS and MADR-S) compared to the last measurement before dosing, and remission will be defined as a (MADRS and MADR-S) score ≤10. In addition, response will also be defined as > 10 points decrease (MADRS and MADR-S) compares to the last measurement before dosing. Response and remission rate at all time points post dose will be analyzed by using Fisher's exact test and univariable logistic regression with odds ratios (OR) and their 95% confidence intervals (CI) and nominal p-values. An additional secondary endpoint will be time to initiation of antidepressant treatment. Time to initiation of antidepressant will be defined as the time (in days) from day 43 to the date when any antidepressant has been prescribed. Subjects will be censored at study discontinuation or completion. The Kaplan-Meier approach will be assessed with the log-rank test.

5.6. Exploratory analysis

The Exploratory analysis will be descriptive between-group differences to support the primary and the secondary analysis as described in section 5.3. In addition, independent predictors will be evaluated using linear (continuous outcome), mixed-linear (repeated outcome), logistic (binary outcome) or Cox (time to event outcome) regression models based on the nature of the data.

5.7. Safety analysis

The number and percentage of subjects experiencing one or more treatment-emergent AEs in the following categories will be summarized. No statistical testing will be performed on the safety data.

- Incidence of AEs by severity
- Incidence of AEs leading to termination from the study
- Incidence of TEAEs
- Incidence of TEAEs by severity
- Incidence of SAEs
