# Supplementary material for: Short-Term and Late-Term Effects of Psilocybin on Symptoms in Major Depression: A Randomized Clinical Trial
Source: JAMA Netw Open. 2026 May 15;9(5):e2612589. doi: 10.1001/jamanetworkopen.2026.12589 (PMC13179547; doi:10.1001/jamanetworkopen.2026.12589)
Supplement: Supplement 2. — eAppendix 1. Psychotherapeutic Support in the PSIPET Study eAppendix 2. Supplemental Results eTable 1. MADRS (Clinician-Rated) and MADRS-S (Self-Report) (Days 1–365) eTable 2. Treatment-Emergent Adverse Events (TEAEs) and List of Treatment Emergent SAEs Reported Throughout the Complete Follow-Up eTable 3. Full-Model Estimates of the Between-Group Difference in Change From Baseline in MADRS Total Score to All Timepoints eTable 4. Full-model Estimates of the Between-Group Difference in Change From Baseline in MADRS-S Total Score to All Timepoints eTable 5. Full-model Estimates of the Between-Group Difference in Change From Baseline in GAD-7 Total Score to All Timepoints eTable 6. Full-model Estimates of the Between-Group Difference in Change from Baseline in SDS Total Score to All Timepoints eTable 7. Full-Model Estimates of the Between-Group Difference in Change From Baseline in EQ5D-5L (Index) to All Timepoints eTable 8. Full-Model Estimates of the Between-Group Difference in Change From Baseline in CGI-S to All Timepoints eTable 9. Full-Model Estimates of the Between-Group Difference in Change From Baseline in CGI-I to All Timepoints eTable 10. MADRS Response Rates eTable 11. MADRS Remission Rates eTable 12. MADRS-S Response Rates eTable 13. MADRS-S Remission Rates [file jamanetwopen-e2612589-s002.pdf]

Supplemental Online Content

Yngwe H, Plavén-Sigray P, Ekman CJ, et al Short-term and late-term effects of psilocybin on symptoms in major depression: a randomized clinical trial. *JAMA Netw Open*. 2026;9(5):e2612589. doi:10.1001/jamanetworkopen.2026.12589

eAppendix 1. Psychotherapeutic Support in the PSIPET Study

eAppendix 2. Supplemental Results

eTable 1. MADRS (Clinician-Rated) and MADRS-S (Self-Report) (Days 1–365)

eTable 2. Treatment-Emergent Adverse Events (TEAEs) and List of Treatment Emergent SAEs Reported Throughout the Complete Follow-Up

eTable 3. Full-Model Estimates of the Between-Group Difference in Change From Baseline in MADRS Total Score to All Timepoints

eTable 4. Full-model Estimates of the Between-Group Difference in Change From Baseline in MADRS-S Total Score to All Timepoints

eTable 5. Full-model Estimates of the Between-Group Difference in Change From Baseline in GAD-7 Total Score to All Timepoints

eTable 6. Full-model Estimates of the Between-Group Difference in Change from Baseline in SDS Total Score to All Timepoints

eTable 7. Full-Model Estimates of the Between-Group Difference in Change From Baseline in EQ5D-5L (Index) to All Timepoints

eTable 8. Full-Model Estimates of the Between-Group Difference in Change From Baseline in CGI-S to All Timepoints

eTable 9. Full-Model Estimates of the Between-Group Difference in Change From Baseline in CGI-I to All Timepoints

eTable 10. MADRS Response Rates

eTable 11. MADRS Remission Rates

eTable 12. MADRS-S Response Rates

eTable 13. MADRS-S Remission Rates

This supplemental material has been provided by the authors to give readers additional information about their work.

© 2026 Yngwe H et al. *JAMA Network Open*.

eAppendix 1. Psychotherapeutic Support in the PSIPET Study

Background of Clinical Facilitators

Facilitators were licensed clinical psychologists with experience in treating major depressive disorder (MDD) and mindfulness approaches.

Each participant was assigned a Lead Facilitator and a Co-Facilitator. Their role was to create a safe and supportive environment during the intervention.

Training Requirements

Clinical Facilitators completed PSIPET-specific training including self-study in the manual based on the Usona treatment manual and a three-day web course provided by the Usona Institute. The training included an overview of the study protocol, inclusion and exclusion criteria, adverse event management, and data handling procedures. It also outlined the Set and Setting (SaS) methodology for guiding psychedelic sessions. Additionally, the protocol detailed techniques for psychotherapeutic support, such as grounding techniques and strategies for handling challenging experiences. The Clinical Facilitators received monthly tutoring in groups throughout the study.

Psychotherapeutic Support Sessions.

Dosing and support sessions took place in a dedicated room equipped with a bed, seating, adaptive light sources and music equipment. Both psychologists were present during dosing and support visits. The psychotherapeutic support was delivered through a structured framework consisting of three distinct session types (see below). The psychotherapeutic approach was non-directive and supportive, based on mindfulness principles. Facilitators were to gain participant trust through empathy, openness, and acceptance, encouraging inner exploration without shaping the dosing experience. They were to validate both positive and challenging experiences to promote psychological growth.

Preparation Session (Approx. two hours, single session day before dosing)

Conducted before dosing, aimed to establish rapport and trust, to assess the participant's history of depression and readiness, and to provide psychoeducation on the potential effects of psilocybin and niacin. It also encouraged openness and curiosity while identifying any vulnerabilities that could impact the session.

Dosing Session (Approx. 7-10 hours, single session)

Dosing occurred in a controlled, supportive environment, with facilitators providing non-directive psychotherapeutic support. Participants were encouraged to stay with their experiences, while facilitators monitored for distress and adverse reactions. Verbal interactions were minimal, focusing on presence and subtle emotional guidance. Grounding techniques could be used to handle anxiety and agitation.

Integration Sessions (Three sessions, approximately 1-2 hours each, at day 1, 8 and 15 post dose)

Held to help participants process their experience. Facilitators encouraged self-reflection without interpreting the experience, guiding participants to explore insights and emotions related to their depression. The goal was to help participants integrate their experience into daily life and promote enduring change.

© 2026 Yngwe H et al. *JAMA Network Open*.

eAppendix 2. Supplemental Results

For response, the odds ratio (OR) on MADRS at day 8 was 2.00 (n = 8 in the psilocybin group vs. 4 in the niacin group; 95% CI, 0.52–8.68). At day 15, the OR increased to 5.50 (n = 11 vs. 2; 95% CI, 1.24–39.13), which remained stable at day 42 (OR = 5.50 [n = 11 vs. 2]; 95% CI, 1.24–39.13). At day 365, the OR was 1.38 (n = 11 vs. 8; 95% CI, 0.45–4.37).

For CGI-S scale, the between-group difference in change from baseline at day 8 was −1.10 points (*P*=.008), remaining significant at day 15 (−1.57; *P*=.001) and day 42 (−1.22; *P*=.003). No significant difference was observed at day 365.

For GAD-7, no significant between-group difference was observed at day 8 (−3.39 [95% CI, −7.04 to 0.26]; *P*=.07). However, differences were significant at day 15 (−4.04 [−7.67 to −0.42]; *P*=.03) and day 42 (−4.08 [−7.67 to −0.49]; *P*=.03), but not at later timepoints (Figure 3D).

For Sheehan Disability Scale (SDS), greater improvement was seen in the psilocybin group starting from day 8 (mean difference, −8.00 [95% CI, −13.04 to −2.97]; *P*=.002). This between-group difference remained significant at all follow-up points, except days 162 and 313, and persisted through day 365 (mean difference, −4.99 [−9.94 to −0.04]; *P*=.05) (Figure 3B).

EQ-5D-5L Index, also improved more in the psilocybin group, with a significant mean difference at day 8 (0.15 [95% CI, 0.02 to 0.27]; *P*=.02), which remained significant through day 102, with additional isolated significant differences later in follow-up (Figure 3C)

eTable 1.

Estimated differences in MADRS and MADRS-S scores (PSI – NIC) across all timepoints from day 1 to day 365. Values represent estimated difference with 95% confidence interval and corresponding *p* value for each analysis: intention-to-treat (ITT), antidepressant censoring (AD Cens.), and psychedelic censoring (Psyc. Cens.).

1a. MADRS (Clinician-Rated)

Day	AD Cens.	ITT	Psyc. Cens
8	-7.40 [-13.05 to -1.75], *P*=.01	-7.27 [-12.89 to -1.65], *P*=.01	-7.26 [-12.88 to -1.65], *P*=.01
15	-11.16 [-16.81 to -5.51], *P*<.001	-11.03 [-16.65 to -5.42], *P*<.001	-11.03 [-16.65 to -5.41], *P*<.001
42	-8.46 [-14.11 to -2.81], *P*=.004	-8.33 [-13.94 to -2.71], *P*=.004	-8.34 [-14.00 to -2.69], *P*=.004
365	-3.95 [-10.22 to 2.31], *P*=.21	-3.68 [-9.30 to 1.94], *P*=.20	-5.06 [-10.77 to 0.65], *P*=.08

1b. MADRS-S (Self-report) (Days 1–365)

Day	AD cens.	ITT	Psyc. cens.
1	-5.60 [-12.22 to 1.03], *P*=.10	-5.53 [-12.01 to 0.95], *P*=.09	-5.57 [-12.05 to 0.91], *P*=.09
2	-9.66 [-16.28 to -3.03], *P*=.005	-9.58 [-16.05 to -3.11], *P*=.004	-9.62 [-16.09 to -3.14], *P*=.004
3	-8.76 [-15.34 to -2.17], *P*=.010	-8.68 [-15.11 to -2.26], *P*=.008	-8.72 [-15.15 to -2.29], *P*=.008

© 2026 Yngwe H et al. *JAMA Network Open*.

4	-8.72 [-15.32 to -2.11], *P*=.01	-8.65 [-15.10 to -2.20], *P*=.009	-8.69 [-15.14 to -2.24], *P*=.009
5	-6.67 [-13.32 to -0.03], *P*=.05	-6.60 [-13.09 to -0.10], *P*=.05	-6.63 [-13.13 to -0.13], *P*=.05
6	-8.59 [-15.24 to -1.94], *P*=.01	-8.50 [-15.01 to -2.00], *P*=.01	-8.54 [-15.05 to -2.04], *P*=.01
7	-7.12 [-13.81 to -0.43], *P*=.04	-7.06 [-13.62 to -0.51], *P*=.03	-7.10 [-13.66 to -0.55], *P*=.03
8	-9.41 [-16.09 to -2.74], *P*=.006	-9.35 [-15.88 to -2.82], *P*=.005	-9.39 [-15.92 to -2.85], *P*=.005
15	-11.14 [-17.79 to -4.50], *P*=.001	-11.08 [-17.58 to -4.58], *P<.001*	-11.11 [-17.61 to -4.61], *P<.001*
42	-12.32 [-18.89 to -5.75], *P<.001*	-12.25 [-18.65 to -5.84], *P<.001*	-12.44 [-18.88 to -6.00], *P<.001*
72	-8.20 [-14.99 to -1.42], *P*=.02	-9.78 [-16.23 to -3.33], *P*=.003	-10.58 [-17.08 to -4.07], *P*=.002
102	-5.64 [-12.47 to 1.20], *P*=.11	-6.60 [-13.01 to -0.19], *P*=.04	-7.48 [-13.95 to -1.01], *P*=.02
132	2.38 [-4.62 to 9.37], *P*=.50	-1.29 [-7.74 to 5.15], *P*=.69	-1.74 [-8.26 to 4.79], *P*=.60
162	-2.73 [-9.90 to 4.43], *P*=.45	-4.65 [-11.10 to 1.80], *P*=.16	-5.08 [-11.67 to 1.50], *P*=.13
192	-3.49 [-10.79 to 3.81], *P*=.35	-5.36 [-11.77 to 1.04], *P*=.10	-6.30 [-12.86 to 0.27], *P*=.06
223	-3.40 [-11.22 to 4.41], *P*=.39	-4.01 [-10.59 to 2.56], *P*=.23	-4.92 [-11.67 to 1.82], *P*=.15
253	-3.02 [-10.75 to 4.71], *P*=.44	-4.56 [-11.01 to 1.89], *P*=.16	-4.99 [-11.64 to 1.66], *P*=.14
283	-7.67 [-15.83 to 0.48], *P*=.07	-7.24 [-13.79 to -0.70], *P*=.03	-8.13 [-14.87 to -1.39], *P*=.02
313	-1.41 [-9.47 to 6.66], *P*=.73	-3.71 [-10.25 to 2.84], *P*=.27	-5.51 [-12.29 to 1.27], *P*=.11
343	-6.79 [-15.40 to 1.81], *P*=.12	-7.07 [-13.88 to -0.26], *P*=.04	-8.49 [-15.47 to -1.51], *P*=.02
365	-1.08 [-9.27 to 7.11], *P*=.79	-1.83 [-8.26 to 4.61], *P*=.58	-3.13 [-9.79 to 3.53], *P*=.35

© 2026 Yngwe H et al. *JAMA Network Open*.

eTable 2a. Treatment-Emergent Adverse Events (TEAEs)

Treatment-emergent adverse events (TEAEs) reported at dosing, day 15, day 42, and day 365, stratified by treatment group. Values indicate number of participants unless otherwise specified. Percentages are reported for adverse events occurring in more than 10% of participants in any group at the respective timepoint. TEAEs and serious adverse events (SAEs) were categorized as related to study medication or study procedures by investigator judgment.

Adverse Events	Psilocybin 25 mg (n=17)	Niacin 100 mg (n=18)
**Overall (all timepoints)**		
Total TEAEs	71	35
Related to study medication, No.	48	15
Related to study procedures, No.	8	8
Total serious adverse events (SAEs), No.	5	5
Related SAEs – study medication	0	0
Related SAEs – study procedures	3	3
Total severe adverse events, No.	5	3
Related severe adverse events – study medication	3	2
Related severe adverse events, study procedures	1	0
**Dosing**		
Any adverse event, No.	37	10
Headache, No. (%)	8 (47.1)	4 (22.2)
Hallucination, No. (%)	5 (29.4)	–
Agitation, No. (%)	3 (17.6)	–
Anxiety, No. (%)	3 (17.6)	1 (5.6) *
Hypertension, No. (%)	3 (17.6)	–
Nausea, No. (%)	2 (11.8)	1 (5.6) *
Paresthesia, No. (%)	2 (11.8)	1 (5.6) *
Serious adverse events, No.	0	0
Severe adverse events, No.	1	0
**Day 15**		
Any adverse event, No.	11	17
Headache, No. (%)	1(5.9) *	4 (22.2)
Cerebrospinal fluid leakage, No. (%)	2 (11.8)	3 (16.7)
Back pain, No. (%)	1(5.9) *	2 (11.1)
Serious adverse events, No.	2	3
Severe adverse events, No.	0	2
**Day 42**		
Any adverse event, No.	15	5
Anxiety, No. (%)	3 (17.6)	1(5.6) *
Serious adverse events, No.	1	0
Severe adverse events, No.	3	0
**Day 365**		
Any adverse event, No.	8	3
Events in >10% of participants	None	None
Serious adverse events, No.	2	2
Severe adverse events, No.	1	1

© 2026 Yngwe H et al. *JAMA Network Open*.

*Below the 10% threshold. If an AE is reported for one group, the number in the other group is reported for comparison, even if below threshold.
Abbreviations: TEAE, treatment-emergent adverse event; SAE, serious adverse event.

A total of ten SAEs were reported, none were considered related to the study drugs (main text).

Six SAEs were clearly related to study procedures such as lumbar puncture (post dural puncture headache *n*=4, back pain *n*=1) and arterial cannulation (pain in extremity *n*=1) causing significant or persistent incapacity or inpatient hospitalization.

Four SAEs were considered unlikely related to study medication because of onset several months after dosing; appendicitis (*n*=1) and suicidal ideation (*n*=1) in PSI, and colitis (*n*=1) and ileus (*n*=1) in NIC. The participant with suicidal ideation required psychiatric outpatient care and this was thus considered an important medical event, the remaining SAEs all caused inpatient hospitalization.

eTable 2b. List of treatment emergent SAEs reported throughout the complete follow-up

Categorized as related to study medication

SAE	Psilocybin	Niacin	Causality	Procedure
None				

Categorized as unlikely related to study medication or related to other study procedures

SAE	Psilocybin	Niacin	Causality	Procedure
Appendicitis	1	0	Unlikely	Study medication
Back pain	0	1	Probable/likely	Lumbar Puncture
Cerebrospinal Fluid Leakage	2	2	Probable/likely	Lumbar Puncture
Colitis	0	1	Unlikely	Study medication
Ileus	0	1	Unlikely	Study medication
Pain in extremity	1	0	Certain	Arterial cannulation
Suicidal Ideation	1	0	Unlikely	Study medication

© 2026 Yngwe H et al. *JAMA Network Open*.

eTable 3. Full-Model Estimates of the Between-Group Difference in Change from Baseline in MADRS Total Score to all Timepoints

Day	Mean difference (95% CI)[2]	SE	P value
8[1]	-7.27 (-12.89 to -1.65)	2.81	P=.01
15	-11.03 (-16.65 to -5.42)	2.81	P<.001
42	-8.33 (-13.94 to -2.71)	2.81	P=.004
365	-3.68 (-9.30 to 1.94)	2.81	P=.19

Abbreviations: CI, confidence interval; SE, standard error

1. Primary outcome

Estimates are active minus niacin; negative values indicate greater improvement with active treatment.

eTable 4. Full-model Estimates of the Between-Group Difference in Change from Baseline in MADRS-S Total Score to all Timepoints.

Day	Mean difference (95% CI)[1]	SE	P value
1	-5.53 (-12.01 to 0.95)	3.28	P=.09
2	-9.58 (-16.05 to -3.11)	3.28	P=.004
3	-8.68 (-15.11 to -2.26)	3.26	P=.008
4	-8.65 (-15.10 to -2.20)	3.27	P=.009
5	-6.60 (-13.09 to -0.10)	3.29	P=.05
6	-8.50 (-15.01 to -2.00)	3.30	P=.01
7	-7.06 (-13.62 to -0.51)	3.32	P=.03
8	-9.35 (-15.88 to -2.82)	3.31	P=.005
15	-11.08 (-17.58 to -4.58)	3.29	P<.001
42	-12.25 (-18.65 to -5.84)	3.25	P<.001
72	-9.78 (-16.23 to -3.33)	3.27	P=.003
102	-6.60 (-13.01 to -0.19)	3.25	P=.04
132	-1.29 (-7.74 to 5.15)	3.27	P=.69
162	-4.65 (-11.10 to 1.80)	3.27	P=.16
192	-5.36 (-11.77 to 1.04)	3.25	P=.10
223	-4.01 (-10.59 to 2.56)	3.33	P=.23
253	-4.56 (-11.01 to 1.89)	3.27	P=.16
283	-7.24 (-13.79 to -0.70)	3.32	P=.03
313	-3.71 (-10.25 to 2.84)	3.32	P=.27
343	-7.07 (-13.88 to -0.26)	3.46	P=.04
365	-1.83 (-8.26 to 4.61)	3.26	P=.58

Abbreviations: CI, confidence interval; SE, standard error

Estimates are active minus niacin; negative values indicate greater improvement with active treatment.

© 2026 Yngwe H et al. *JAMA Network Open*.

eTable 5. Full-model Estimates of the Between-Group Difference in Change from Baseline in GAD-7 Total Score to all Timepoints.

Day	Mean difference (95% CI)[1]	SE	P value
8	-3.39 (-7.04 to 0.26)	1.85	P=.07
15	-4.04 (-7.67 to -0.42)	1.83	P=.03
42	-4.08 (-7.67 to -0.49)	1.82	P=.03
72	-3.39 (-7.02 to 0.23)	1.83	P=.07
102	-2.50 (-6.08 to 1.07)	1.81	P=.17
132	-0.04 (-3.65 to 3.56)	1.82	P=.98
162	-0.80 (-4.37 to 2.77)	1.81	P=.66
192	-1.98 (-5.55 to 1.60)	1.81	P=.28
223	-3.50 (-7.21 to 0.20)	1.88	P=.06
253	-2.21 (-5.81 to 1.40)	1.82	P=.23
283	-2.91 (-6.60 to 0.77)	1.87	P=.12
313	-0.49 (-4.17 to 3.20)	1.87	P=.79
343	-3.80 (-7.70 to 0.09)	1.97	P=.06
365	-2.28 (-5.90 to 1.34)	1.83	P=.22

Abbreviations: CI, confidence interval; SE, standard error

Estimates are active minus niacin; negative values indicate greater improvement with active treatment.

eTable 6. Full-model Estimates of the Between-Group Difference in Change from Baseline in SDS Total Score to all Timepoints.

Day	Mean difference (95% CI)[1]	SE	P value
8	-8.00 (-13.04 to -2.97)	2.54	P=.002
15	-8.13 (-13.10 to -3.16)	2.51	P=.002
42	-8.92 (-13.81 to -4.02)	2.47	P<.001
72	-5.77 (-10.70 to -0.85)	2.49	P=.02
102	-7.39 (-12.28 to -2.50)	2.47	P=.003
132	-5.10 (-10.03 to -0.18)	2.49	P=.04
162	-4.89 (-9.82 to 0.04)	2.49	P=.05
192	-6.15 (-11.05 to -1.26)	2.47	P=.01
223	-6.14 (-11.17 to -1.12)	2.54	P=.02
253	-7.02 (-11.95 to -2.09)	2.49	P=.006
283	-6.61 (-11.61 to -1.60)	2.53	P=.01
313	-4.48 (-9.49 to 0.53)	2.53	P=.08
343	-6.19 (-11.41 to -0.98)	2.64	P=.02
365	-4.99 (-9.94 to -0.04)	2.50	P=.05

Abbreviations: CI, confidence interval; SE, standard error

Estimates are active minus niacin; negative values indicate greater improvement with active treatment.

© 2026 Yngwe H et al. *JAMA Network Open*.

eTable 7. Full-model Estimates of the Between-Group Difference in Change from Baseline in EQ5D-5L (Index) to all Timepoints.

Day	Mean difference (95% CI)[1]	SE	P value
8	0.15 (0.02 to 0.27)	0.06	P=.02
15	0.22 (0.09 to 0.34)	0.06	P<.001
42	0.22 (0.10 to 0.34)	0.06	P<.001
72	0.14 (0.02 to 0.27)	0.06	P=.02
102	0.22 (0.10 to 0.34)	0.06	P<.001
132	0.07 (-0.06 to 0.19)	0.06	P=.30
162	0.12 (-0.01 to 0.24)	0.06	P=.06
192	0.12 (-0.00 to 0.24)	0.06	P=.06
223	0.14 (0.01 to 0.27)	0.07	P=.04
253	0.17 (0.04 to 0.29)	0.06	P=.008
283	0.18 (0.06 to 0.31)	0.07	P=.005
313	0.09 (-0.04 to 0.22)	0.06	P=.17
343	0.14 (0.00 to 0.28)	0.07	P=.05
365	0.14 (0.02 to 0.27)	0.06	P=.02

Abbreviations: CI, confidence interval; SE, standard error

Estimates are active minus niacin; positive values indicate greater improvement with active treatment.

eTable 8. Full-model Estimates of the Between-Group Difference in Change from Baseline in CGI-S to all Timepoints.

Day	Mean difference (95% CI)[1]	SE	P value
8	-1.10 (-1.90 to -0.30)	0.40	P=.008
15	-1.57 (-2.49 to -0.64)	0.47	P=.001
42	-1.22 (-2.01 to -0.42)	0.40	P=.003
365	-0.57 (-1.37 to 0.23)	0.40	P=.16

Abbreviations: CI, confidence interval; SE, standard error

Estimates are active minus niacin; negative values indicate greater improvement with active treatment.

eTable 9. Full-model Estimates of the Between-Group Difference in Change from Baseline in CGI-I to all Timepoints.

Day	Mean difference (95% CI)[1]	SE	P value
8	-1.29 (-2.13 to -0.46)	0.42	P=.003
15	-1.86 (-2.84 to -0.88)	0.49	P<.001
42	-1.35 (-2.19 to -0.52)	0.42	P=.002
365	-1.18 (-2.01 to -0.34)	0.42	P=.006

Abbreviations: CI, confidence interval; SE, standard error

Estimates are active minus niacin; negative values indicate greater improvement with active treatment.

© 2026 Yngwe H et al. *JAMA Network Open*.

eTable 10. MADRS Response Rates

Day	Active, n/N (%)	Placebo, n/N (%)	Absolute difference, percentage points (95% CI)	OR (95% CI)	P value
8	8/17 (47.1%)	4/17 (23.5%)	23.5 (-13.5 to 60.5)	2.00 (0.52 to 8.68)	P=.28
15	11/17 (64.7%)	2/17 (11.8%)	52.9 (19.7 to 86.2)	5.50 (1.24 to 39.13)	P=.005
42	11/17 (64.7%)	2/17 (11.8%)	52.9 (19.7 to 86.2)	5.50 (1.24 to 39.13)	P=.005
365	11/17 (64.7%)	8/17 (47.1%)	17.6 (-21.1 to 56.4)	1.38 (0.45 to 4.37)	P=.49

Abbreviations: CI, confidence interval; MADRS, Montgomery-Asberg Depression Rating Scale; OR, odds ratio.

eTable 11. MADRS Remission Rates

Day	Active, n/N (%)	Placebo, n/N (%)	Absolute difference, percentage points (95% CI)	OR (95% CI)	P value
8	8/17 (47.1%)	2/17 (11.8%)	35.3 (1.2 to 69.4)	4.00 (0.85 to 29.17)	P=.06
15	10/17 (58.8%)	1/17 (5.9%)	52.9 (21.1 to 84.8)	10.00 (1.64 to 193.97)	P=.003
42	9/17 (52.9%)	1/17 (5.9%)	47.1 (14.9 to 79.2)	9.00 (1.45 to 175.44)	P=.008
365	9/17 (52.9%)	7/17 (41.2%)	11.8 (-27.4 to 51.0)	1.29 (0.39 to 4.36)	P=.73

Abbreviations: CI, confidence interval; MADRS, Montgomery-Asberg Depression Rating Scale; OR, odds ratio.

© 2026 Yngwe H et al. *JAMA Network Open*.

eTable 12. MADRS-S Response Rates

Day	Active, n/N (%)	Placebo, n/N (%)	Absolute difference, percentage points (95% CI)	OR (95% CI)	P value
1	6/16 (37.5%)	2/16 (12.5%)	25.0 (-10.0 to 60.0)	3.00 (0.59 to 22.65)	P=.22
2	7/15 (46.7%)	2/16 (12.5%)	34.2 (-2.3 to 70.6)	3.73 (0.76 to 27.83)	P=.09
3	9/17 (52.9%)	2/16 (12.5%)	40.4 (5.6 to 75.2)	4.24 (0.92 to 30.66)	P=.04
4	9/15 (60.0%)	2/17 (11.8%)	48.2 (12.8 to 83.7)	5.10 (1.10 to 37.07)	P=.01
5	8/15 (53.3%)	3/15 (20.0%)	33.3 (-5.7 to 72.4)	2.67 (0.63 to 14.02)	P=.13
6	8/16 (50.0%)	2/14 (14.3%)	35.7 (-1.6 to 73.0)	3.50 (0.73 to 25.84)	P=.09
7	8/14 (57.1%)	2/14 (14.3%)	42.9 (4.0 to 81.7)	4.00 (0.82 to 29.76)	P=.05
8	10/15 (66.7%)	3/14 (21.4%)	45.2 (6.2 to 84.3)	3.11 (0.77 to 16.07)	P=.04
15	11/15 (73.3%)	3/15 (20.0%)	53.3 (16.5 to 90.2)	3.67 (0.92 to 18.70)	P=.01
42	12/17 (70.6%)	4/17 (23.5%)	47.1 (11.6 to 82.5)	3.00 (0.85 to 12.47)	P=.02
72	11/17 (64.7%)	6/15 (40.0%)	24.7 (-15.2 to 64.6)	1.62 (0.49 to 5.70)	P=.30
102	9/17 (52.9%)	8/17 (47.1%)	5.9 (-33.6 to 45.3)	1.12 (0.35 to 3.67)	P>.99
132	9/16 (56.2%)	9/16 (56.2%)	0.0 (-34.4 to 34.4)	1.00 (0.31 to 3.21)	P>.99
162	8/17 (47.1%)	7/15 (46.7%)	0.4 (-34.6 to 35.4)	1.01 (0.29 to 3.52)	P>.99
192	9/17 (52.9%)	9/17 (52.9%)	0.0 (-33.6 to 33.6)	1.00 (0.32 to 3.17)	P>.99
223	11/14 (78.6%)	8/13 (61.5%)	17.0 (-24.5 to 58.5)	1.28 (0.39 to 4.26)	P=.58
253	10/16 (62.5%)	8/16 (50.0%)	12.5 (-27.9 to 52.9)	1.25 (0.39 to 4.07)	P=.72
283	10/15 (66.7%)	4/13 (30.8%)	35.9 (-5.9 to 77.7)	2.17 (0.57 to 9.45)	P=.13
313	9/14 (64.3%)	8/14 (57.1%)	7.1 (-36.1 to 50.4)	1.12 (0.33 to 3.82)	P>.99
343	8/11 (72.7%)	5/10 (50.0%)	22.7 (-27.5 to 72.9)	1.45 (0.36 to 6.24)	P=.53
365	11/16 (68.8%)	13/17 (76.5%)	-7.7 (-44.2 to 28.7)	0.90 (0.31 to 2.59)	P=.92

Abbreviations: CI, confidence interval; MADRS, Montgomery-Asberg Depression Rating Scale-Self-rated; OR, odds ratio.

© 2026 Yngwe H et al. *JAMA Network Open*.

eTable 13. MADRS-S Remission Rates

Day	Active, n/N (%)	Placebo, n/N (%)	Absolute difference, percentage points (95% CI)	OR (95% CI)	P value
1	2/16 (12.5%)	0/16 (0.0%)	12.5 (-10.0 to 35.0)	NE[1]	NE
2	4/15 (26.7%)	0/16 (0.0%)	26.7 (-2.2 to 55.5)	NE	NE
3	5/17 (29.4%)	1/16 (6.2%)	23.2 (-7.6 to 53.9)	4.71 (0.66 to 95.44)	P=.20
4	6/15 (40.0%)	1/17 (5.9%)	34.1 (0.6 to 67.6)	6.80 (1.00 to 136.30)	P=.06
5	5/15 (33.3%)	2/15 (13.3%)	20.0 (-16.1 to 56.1)	2.50 (0.46 to 19.42)	P=.39
6	6/16 (37.5%)	1/14 (7.1%)	30.4 (-3.6 to 64.3)	5.25 (0.77 to 105.61)	P=.13
7	6/14 (42.9%)	2/14 (14.3%)	28.6 (-10.3 to 67.5)	3.00 (0.58 to 22.97)	P=.21
8	8/15 (53.3%)	2/14 (14.3%)	39.0 (0.9 to 77.2)	3.73 (0.77 to 27.66)	P=.07
15	8/15 (53.3%)	2/15 (13.3%)	40.0 (2.8 to 77.2)	4.00 (0.83 to 29.54)	P=.05
42	8/17 (47.1%)	1/17 (5.9%)	41.2 (9.1 to 73.3)	8.00 (1.27 to 156.90)	P=.02
72	8/17 (47.1%)	1/15 (6.7%)	40.4 (7.2 to 73.5)	7.06 (1.11 to 138.94)	P=.03
102	8/17 (47.1%)	1/17 (5.9%)	41.2 (9.1 to 73.3)	8.00 (1.27 to 156.90)	P=.02
132	6/16 (37.5%)	4/16 (25.0%)	12.5 (-25.6 to 50.6)	1.50 (0.36 to 6.84)	P=.70
162	5/17 (29.4%)	2/15 (13.3%)	16.1 (-17.9 to 50.0)	2.21 (0.41 to 17.02)	P=.50
192	6/17 (35.3%)	1/17 (5.9%)	29.4 (-1.8 to 60.6)	6.00 (0.89 to 119.81)	P=.09
223	7/14 (50.0%)	3/13 (23.1%)	26.9 (-15.3 to 69.1)	2.17 (0.49 to 11.75)	P=.29
253	7/16 (43.8%)	3/16 (18.8%)	25.0 (-12.2 to 62.2)	2.33 (0.54 to 12.35)	P=.25
283	7/15 (46.7%)	3/13 (23.1%)	23.6 (-17.7 to 64.9)	2.02 (0.46 to 10.90)	P=.37
313	6/14 (42.9%)	3/14 (21.4%)	21.4 (-19.4 to 62.2)	2.00 (0.43 to 11.01)	P=.42
343	4/11 (36.4%)	2/10 (20.0%)	16.4 (-30.9 to 63.6)	1.82 (0.29 to 15.25)	P=.73
365	7/16 (43.8%)	5/17 (29.4%)	14.3 (-24.3 to 53.0)	1.49 (0.39 to 5.95)	P=.62

Abbreviations: CI, confidence interval; MADRS, Montgomery-Asberg Depression Rating Scale-Self-rated; OR, odds ratio.

1. Not estimable (zero cell counts)

© 2026 Yngwe H et al. *JAMA Network Open*.
