# Supplementary material for: Short-Term and Late-Term Effects of Psilocybin on Symptoms in Major Depression: A Randomized Clinical Trial
Source: JAMA Netw Open. 2026 May 15;9(5):e2612589. doi: 10.1001/jamanetworkopen.2026.12589 (PMC13179547; doi:10.1001/jamanetworkopen.2026.12589)
Supplement: Supplement 3. — Data Sharing Statement [file jamanetwopen-e2612589-s003.pdf]

Data Sharing Statement

Yngwe. Short-Term and Late-Term Effects of Psilocybin on Symptoms in Major Depression. *JAMA Netw Open*. Published May 15, 2026. doi:10.1001/jamanetworkopen.2026.12589

Data

Additional Information: ClinicalTrials.gov Identifier: NCT04630964 EudraCT-number: 2020-002790-94

Data available: Deidentified individual participant data underlying the results reported in this article may be made available from the corresponding author upon reasonable request, provided that no legal, regulatory, or ethical barriers to data sharing apply.
